# Divergent Synthesis of Ultrabright and Dendritic Xanthenes for Enhanced Click‐Chemistry‐Based Bioimaging

**DOI:** 10.1002/chem.202202633

**Published:** 2022-12-07

**Authors:** Luis Montiel, Fabio Spada, Antony Crisp, Sascha Serdjukow, Thomas Carell, Thomas Frischmuth

**Affiliations:** ^1^ Baseclick GmbH Floriansbogen 2–4 82061 Neuried (Munich) Germany; ^2^ Department of Chemistry Institut für Chemische Epigenetik München (ICEM) Ludwig-Maximilians-Universität München (LMU) Butenandtstr. 5–13 81377 Munich Germany

**Keywords:** click chemistry, dendrons, ethynyldeoxyuridines, fluorescein, rhodamines

## Abstract

Biorthogonal labelling with fluorescent small molecules is an indispensable tool for diagnostic and biomedical applications. In dye‐based 5‐ethynyl‐2′‐deoxyuridine (EdU) cell proliferation assays, augmentation of the fluorescent signal entails an overall enhancement in the sensitivity and quality of the method. To this end, a rapid, divergent synthetic procedure that provides *ready‐to‐click* pH‐insensitive rhodamine dyes exhibiting outstanding brightness was established. Compared to the shortest available synthesis of related high quantum‐yielding rhodamines, two fewer synthetic steps are required. In a head‐to‐head imaging comparison involving copper(I)‐catalyzed azide alkyne cycloaddition reactions with *in vitro* administered EdU, our new 3,3‐difluoroazetidine rhodamine azide outperformed the popular 5‐TAMRA‐azide, making it among the best available choices when it comes to fluorescent imaging of DNA. In a further exploration of the fluorescence properties of these dyes, a set of bis‐MPA dendrons carrying multiple fluorescein or rhodamine units was prepared by branching click chemistry. Fluorescence self‐quenching of fluorescein‐ and rhodamine‐functionalized dendrons limited the suitability of the dyes as labels in EdU‐based experiments but provided new insights into these effects.

## Graphical abstract

**A rapid and divergent synthetic route** that provides ready‐to‐click pH‐insensitive rhodamine dyes exhibiting outstanding brightness was developed. In EdU cell proliferation assays, our 3,3‐difluoroazetidine rhodamine azide outperformed the popular 5‐TAMRA‐azide. A set of bis‐MPA dendrons carrying multiple fluorescein or our rhodamine revealed self‐quenching effects, suggesting potential application as FRET quenchers.

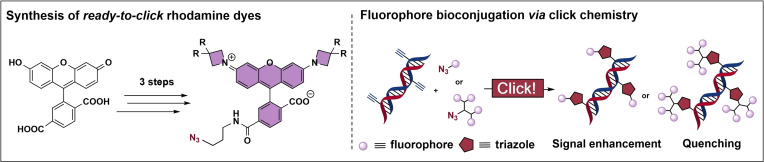

## Introduction

In the field of bioimaging and diagnostics, fluorescent labelling has emerged as a potent tool for the elucidation of structures, dynamics, interactions and functions of biomolecules such as proteins,^[1]^ nucleic acids,^[2]^ polysaccharides^[3]^ and lipids.^[4]^ Furthermore, efficient fluorescence resonance energy transfer (FRET)‐compatible fluorophores and quenchers are increasingly sought after for their use in real‐time PCR applications, including as components of molecular beacons,^[5]^ TaqMan probes,^[6]^ and Scorpion primers.^[7]^ In the context of cell proliferation detection, fluorescence labelling of nucleic acids helps to assess genotoxicity of new pharmaceuticals and to evaluate anticancer drugs.^[8]^ In order to obtain an effective image and produce high signal‐to‐noise ratios, probes must exhibit strong fluorescent signals. A known disadvantage of fluorescent labels over traditional radioactive labels is their moderately lower sensitivity.^[9]^ This difference can lead to undesirable results, especially in the framework of oncology, where slowly proliferating cancerogenic cells have been reported to escape detection.^[10]^ Upon chemical functionalization with multiple dyes to enhance fluorescence signal, proteins and other biomolecules can become inactivated due to their large structural alteration.^[11]^ Fluorescence brightness, that is, the product of a fluorophore's extinction coefficient and fluorescence quantum yield (ϵ ⋅ φ), is used to compare the fluorescent properties of different dyes.^[12]^ Therefore, to overcome concerns related to fluorescence intensity and biomolecule alteration, fluorophores with enhanced brightness values are highly desirable.

Click chemistry remains the gold standard labelling strategy used in imaging experiments to attach fluorophores to the biomolecule of interest,^[13]^ with EdU‐based assays being particularly useful for cell proliferation detection, and where the clickable thymidine analogue EdU is metabolically incorporated during active DNA synthesis.[^14^, ^15^] Unlike halogenated thymidine analogues such as 5‐bromo‐2’‐deoxyuridine (BrdU), EdU assays do not require harsh DNA denaturing conditions, thus preserving cellular and tissue integrity.[^14^, ^16^]

In this study, we disclose a facile, three‐step synthesis of highly bright *ready‐to‐click* rhodamine dyes and systematically explore their photophysical properties. Moreover, we demonstrate the applicability of these dyes in the framework of EdU‐based assays. In a further exploration of their synthetic utility and fluorescent properties, we prepare dendrons containing multiple branched fluorophores with the aim of augmenting fluorescence signal without increasing the number of labelling‐sites within a given alkyne‐modified DNA molecule.^[17]^ The use of related, but not identical constructs has been demonstrated to enable a wide variety of applications, particularly in drug and gene delivery,^[18]^ cancer therapy^[19]^ and tissue engineering.^[20]^ Controlled synthesis of large dendritic scaffolds remains a considerable challenge, particularly where structural characterization is concerned, thus prompting the need for new and improved synthetic strategies. In the context of fluorescent scaffolds, self‐quenching has also proven problematic.[^9^, ^21^, ^22^] In certain instances, fluorescence intensity in antibody‐ and DNA‐conjugates has nonetheless been successfully enhanced, prompting us to investigate this approach ourselves.[^9^, ^23^, ^24^]

To such an end, we demonstrate the synthesis by means of click chemistry, of a set of bis‐MPA dendrons carrying either multiple fluorescein units, or our best‐performing rhodamine. After subsequent characterization of the photophysical properties of these fluorogenic compounds, we evaluate their suitability as fluorescent labels for EdU‐based experiments as well as other potential applications.

## Results and Discussion

Rhodamines, first described in the 1880s,^[25]^ are a family of xanthene dyes which exhibit excellent brightness, exceptional photostability and low pH‐sensitivity.[^26^, ^27^] Traditionally, rhodamine dyes (**3**) have been synthesized by means of an acid‐catalyzed condensation reaction between a phthalic anhydride (**1**) and an aminophenol (**2**) (Scheme 1.1). This reaction is, however, characterized by harsh conditions, low yields, mixtures of regioisomers and incompatibilities with several functional groups.[^26^, ^28^, ^29^, ^30^] With the aim to omit this step, Lavis and co‐workers reported a synthesis based on the use of fluorescein ditriflates (**5**) as key intermediates and a Pd‐catalyzed cross coupling for the formation of C−N bonds (Scheme 1.2).[^28^, ^31^] Following this strategy, milder reaction conditions, higher yields and the use of a wide range of nitrogen nucleophiles were successfully carried out. In this work, the latter methodology has been modified for the synthesis of *ready‐to‐click* rhodamine dyes in a facile and straightforward manner for bioconjugation with DNA via click chemistry. This new route only comprises 3 synthetic steps and a late‐stage formation of fluorescein azide ditriflate **9**, which enables a divergent synthesis of different rhodamine dyes through Buchwald–Hartwig cross‐coupling (Scheme 1.3).

**Scheme 1:**
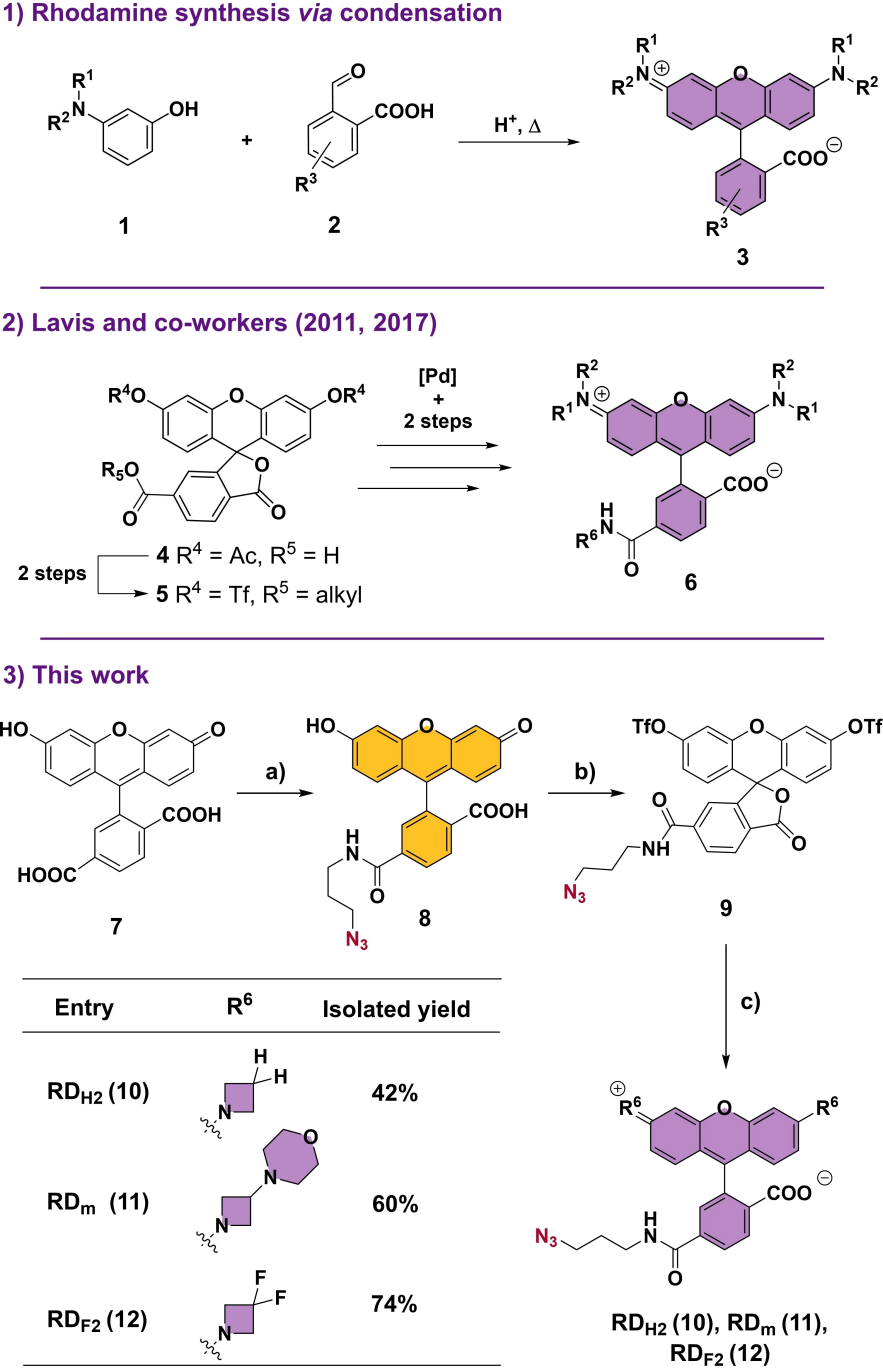
Different strategies for the synthesis of rhodamine dyes. a) DSC, DMAP, Et_3_N, DMF, 1 h, rt, then 3‐azidopropan‐1‐amine, 2 h, rt, 70 %, b) Py, Tf_2_O, CH_2_Cl_2_, 16 h, 0 °C→rt, 81 %, c) amine⋅HCl, Pd_2_(dba)_3_, XPhos, Cs_2_CO_3_, dioxane, 4 h, 100 °C, 42–74 %. DSC=*N,N′* disuccinimidyl carbonate. DMAP=4‐dimethylaminopyridine. DMF=*N,N‐*dimethylformamide. Py=pyridine. Tf_2_O=trifluoromethanesulfonic anhydride. dba=dibenzylideneacetone.

The synthetic pathway starts with 6‐carboxyfluorescein (**7**) as an inexpensive, isomerically pure starting material. The 6‐carboxyl group was converted *in situ* in an *n‐*hydroxysuccinimide (NHS) ester using a catalytic amount of 4‐(Dimethylamino)pyridine (DMAP), triethylamine and *N,N′‐*disuccinimidyl carbonate (DSC) as a coupling reagent. Upon activation as an NHS ester, the bifunctional linker azidopropan‐1‐amine was added to provide **fluorescein azide** (**8**) as a bright orange solid in 70 % yield. The next step involved conversion of the phenolic groups into triflates using trifluoromethanesulfonic anhydride (Tf_2_O) and pyridine to yield fluorescein azide ditriflate **9** in 81 % yield as the key intermediate of the synthesis. Subsequent Buchwald‐Hartwig cross‐couplings were performed using Pd_2_(dba)_3_/XPhos as the catalytic system and Cs_2_CO_3_ as base. In a similar approach as previously reported in the literature,[^30^, ^32^] catalyst and ligand loadings were increased to suppress triflate hydrolysis, which represented the main side‐reaction of this step. Different 3‐substituted azetidines were used as nitrogen nucleophiles in the cross‐coupling reaction since rhodamine dyes containing azetidine rings were found to exhibit considerable quantum yields.[^28^, ^31^] In this manner, dyes **RD_H2_** (**10**), **RD_m_** (**11**), and **RD_F2_** (**12**) were successfully synthesized as pink‐to‐purple solids in 42 %, 60 % and 74 % yield, respectively. A study of the photophysical properties of these dyes was performed by UV‐Vis and fluorescence spectroscopic techniques at different pH values (Table 1). In order to observe the properties of the zwitterionic and cationic forms of the rhodamines,^[26]^ measurements were taken at close‐to physiological (pH=7.3) and acidic (pH=1.9) pH values, respectively (see Supporting Information, Figures S19–21 for the calculated p*k*_a_ values). As for the absorption and emission maxima (λ_abs_, λ_em_), the synthesized dyes absorb green and green‐to‐yellow light (λ_abs_=530–556 nm) and emit green‐to‐yellow and yellow light (λ_em_=554–580 nm). A larger hyposochromic 5(blue) shift in both λ_abs_ and λ_em_ was observed when using azetidine derivatives containing more electron‐withdrawing groups. Thus, λ_abs_ and λ_em_ of these dyes obey the following tendency: **RD_H2_** >**RD_m_** >**RD_F2_**. This set of rhodamine dyes showed high ϵ_max_ values (ϵ_max_=53000 to 68000 M^−1^ cm^−1^) with low pH sensitivity. Likewise, the rhodamines showed high to very high quantum yield values (φ=0.67 to 0.89) with minimal pH‐dependent effects, except for **RD_m_** (φ=0.67 at pH 7.3 and φ=0.86 at pH 1.9). This quenching behavior, as described in the literature,[^28^, ^34^, ^35^] is indicative of an intramolecular photoinduced electron transfer (PeT) due to the presence of unprotonated morpholino amines. Consequently, the quantum yield value of **RD_m_** increased dramatically at pH 1.9.

**Table 1:** Photophysical properties of the synthesized rhodamine dyes.

	pH 7.3^[a]^				pH 1.9^[b]^			
Fluorophore	λ_abs_/λ_em_ [nm]	ϵ_max_^[c]^ [M^−1^ cm^−1^]	φ^[d]^	ϵ_max_ ⋅ φ [M^−^1 cm^−1^]	λ_abs_/λ_em_ [nm]	ϵ_max_^[c]^ [M^−1^ cm^−1^]	φ^[d]^	ϵ_max_ ⋅ φ [M^−^1 cm^−1^]
**RD_H2_**	554/576	64000	0.74	47360	556/580	63000	0.67	42210
**RD_m_**	547/569	53000	0.67	35510	536/560	55000	0.86	47300
**RD_F2_**	530/554	60000	0.89	53400	533/558	68000	0.87	59160

[a] Measurements were taken in 10 mM HEPES, pH 7.3 buffer at room temperature. [b] Measurements were taken in 0.1 % TFA aqueous solution at room temperature. [c] Maximum extinction coefficients (ϵ_max_) were calculated by a linear regression analysis obeying the Beer‐Lambert law. [d] Quantum yields (φ) were determined using the comparative method and rhodamine 6G (φ=0.95 in EtOH)^[33]^ as reference.

**Figure 1:**
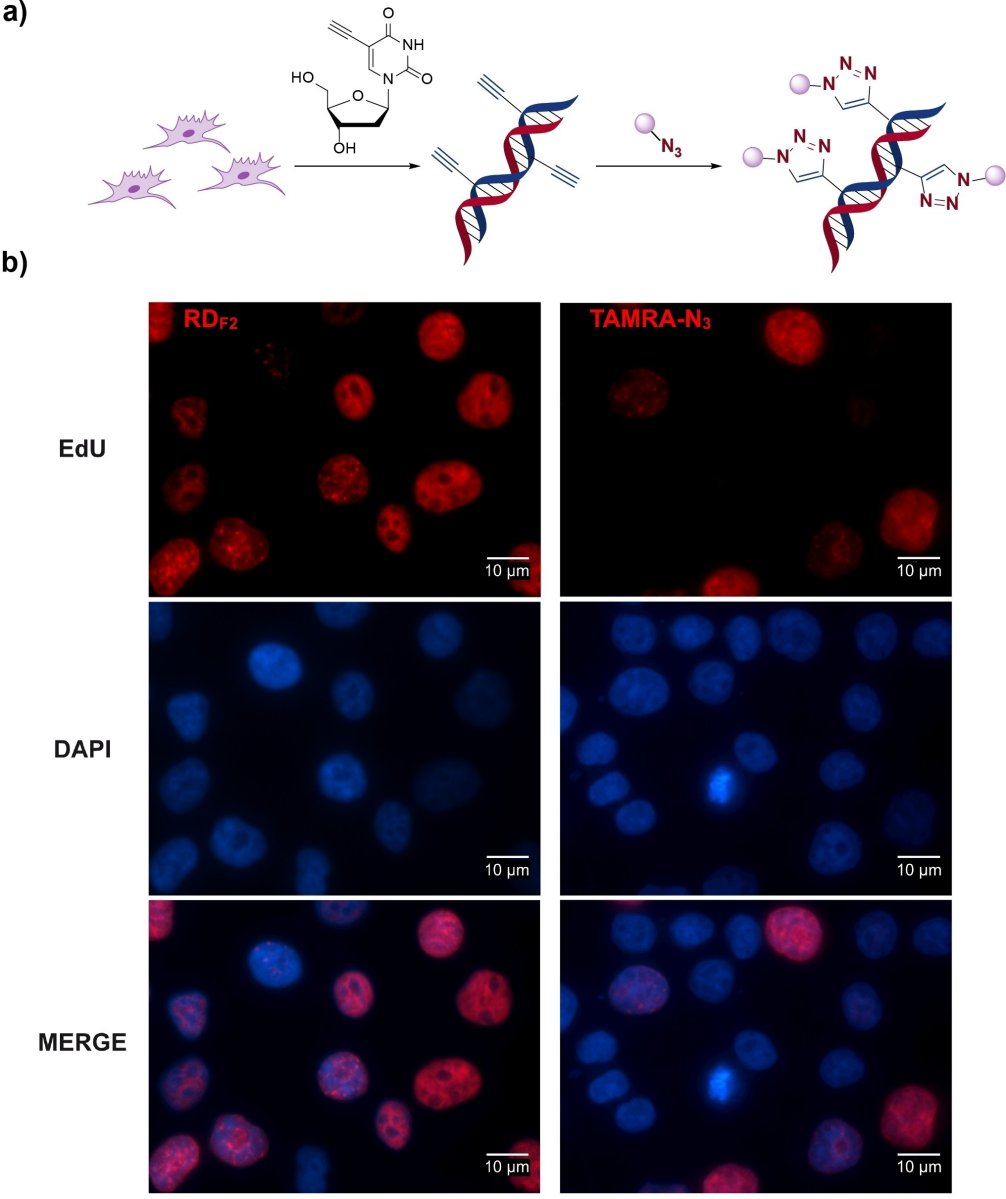
EdU cell proliferation assays with RD_F2_ and 5‐TAMRA‐PEG3‐Azide. a) Schematic workflow of the EdU cell proliferation assay. Cells were grown and incubated with EdU. After cell fixation, the alkyne‐modified DNA was bioconjugated with RD_F2_ or 5‐TAMRA‐PEG3‐azide via Cu‐catalyzed click reaction and detected by fluorescence microscopy. b) Fluorescence microscopy images of EdU‐pulsed HEK‐293T cells after click reactions with RD_F2_ (red, left panels) and 5‐TAMRA‐PEG3‐azide (red, right panels). Blue signals represent nuclear counterstaining with DAPI.

Given its remarkably high extinction coefficient, quantum yield and low pH‐dependence, **RD_F2_** dye (ϵ_max_=60000 M^−1^cm^1^ and φ=0.89 at pH 7.3) stood as the best‐performing dye in terms of fluorescence brightness. With this in mind, we assessed the applicability of **RD_F2_** to an EdU cell proliferation assay (Figure 1a). HEK‐293T cells were pulsed for 2 h with EdU, fixed, permeabilized and Cu‐catalyzed *in situ* click reactions were performed in parallel with **RD_F2_** and the well‐established 5‐TAMRA‐PEG3‐azide. After image acquisition by fluorescence microscopy (Figure 1b), both dyes provided images with characteristic nuclear patterns of DNA replication and similar fluorescence intensity. The emission filter used in this assay, however, exhibited an acquisition window overlapping more extensively the emission spectrum of TAMRA in detriment of that of **RD_F2_**. This indicates, therefore, that **RD_F2_** performs at least as robustly as the well‐established TAMRA dye upon click‐mediated *in situ* EdU labelling.

**Scheme 2:**
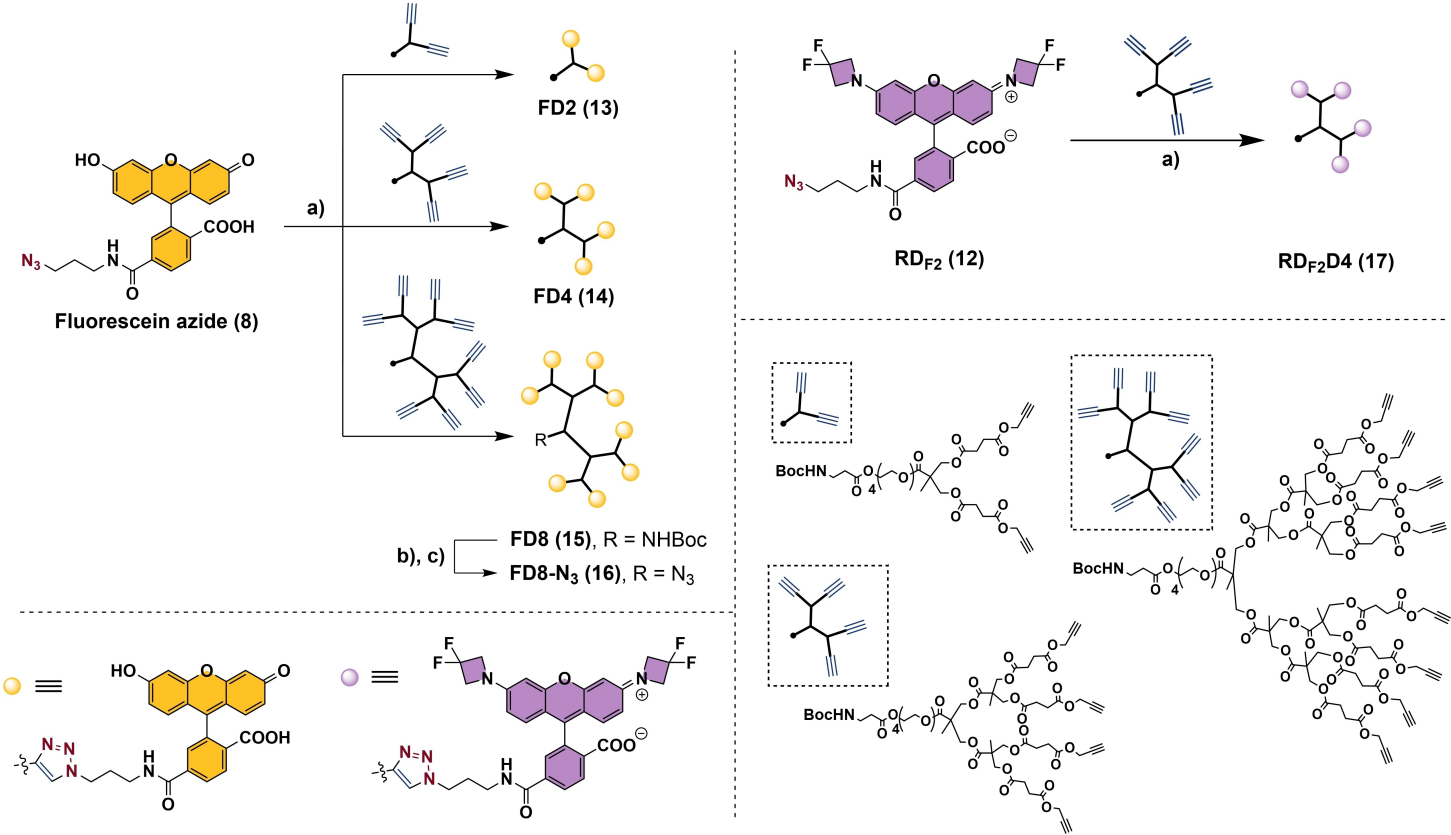
Synthesis and structure of fluorescein‐ and rhodamine‐based dendritic dyes. a) CuBr, PMDTA, DMF, 45 °C, 4 h, 61 % (**FD2**), 67 % (**FD4**), 40 % (**FD8**), 85 % (**RD_F2_D4**). b) TFA, 2 h, rt, quantitative yield. c) Azido‐PEG4‐NHS ester, Et_3_N, DMF, 1 h, 67 %. PMDTA=pentamethyldiethylenetriamine. DMF=*N,N‐*dimethylformamide. TFA=trifluoroacetic acid. Azido‐PEG4‐NHS=15‐Azido‐4,7,10,13‐tetraoxa‐pentadecanoic acid succinimidyl ester.

Encouraged by these results, we investigated the use of dye‐containing dendrons as fluorescent labels to further amplify the fluorescence signal without increasing the number of labelling‐sites on DNA (Scheme 2). We therefore synthesized and characterized the photophysical properties of a family of fluorescein‐based dendritic dyes carrying two (**FD2**, **12**), four (**FD4**, **13**) and eight (**FD8**, **14**) branched fluorescein substituents. In order to synthesize these multivalent fluorescent dyes, we chose a set of 2,2‐bis(methylol)propionic acid (bis‐MPA) polyester dendrons as functional, biodegradable and low‐cytotoxic polymeric scaffolds suitable for biological applications.^[17]^ The present strategy consisted of functionalizing the different dendritic structures with units of **fluorescein azide** via a copper‐catalyzed click reaction to yield **FD2**, **FD4** and **FD8** in 61 %, 67 % and 40 % yield, respectively. **FD8** was further functionalized to include an azide moiety into its focal point to enable a possible bioconjugation via click chemistry. Upon treatment of **FD8** with trifluoroacetic acid, the Boc‐protected amine was released in quantitative yields. The subsequent coupling reaction with the NHS ester of an azide‐containing linker afforded **FD8‐N_3_** (**16**) in 67 % yield.

**Figure 2:**
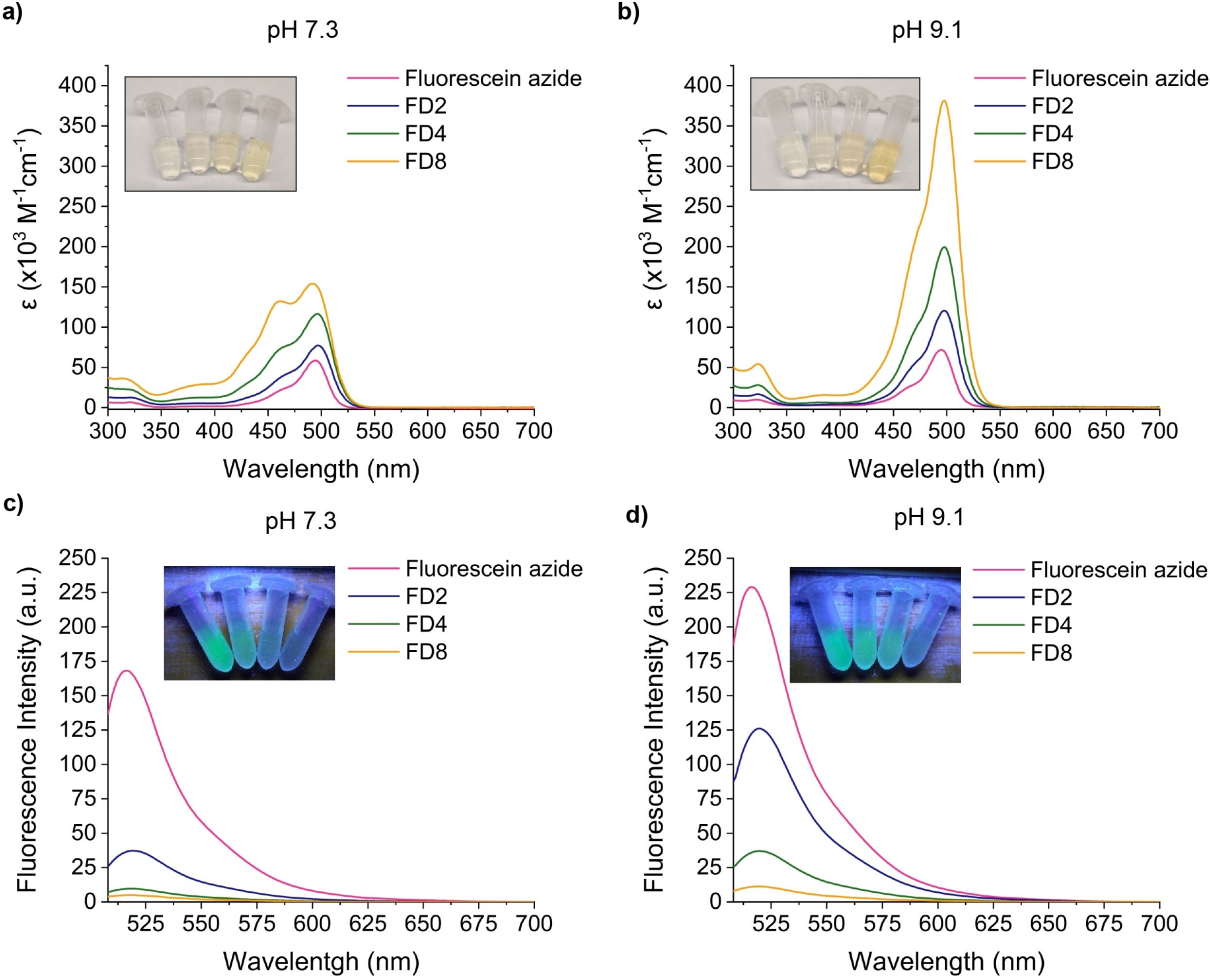
Absorption and emission spectra of fluorescein azide, FD2, FD4 and FD8 at different pH values. a) Absorption spectra measured in 10 mM HEPES, pH 7.3 buffer at a concentration of 2 μM. b) Absorption spectra measured in 10 mM sodium borate, pH 9.1 buffer at a concentration of 2 μM. c) Emission spectra measured in 10 mM HEPES, pH 7.3 buffer at a concentration of 0.2 μM. λ_ex_ =495 nm (fluorescein azide), 497 nm (FD2 and FD4) and 492 nm (FD8). d) Emission spectra measured in 10 mM sodium borate, pH 9.1 buffer at a concentration of 0.2 μM. λ_ex_ =495 nm (fluorescein azide) and 498 nm (FD2, FD4 and FD8). Inset: Solutions in the photographs correspond, from left to right, to fluorescein azide, FD2, FD4 and FD8 at 2 μM (absorbance) or 0.2 μM (emission). Absorbance and emission scans are averages (n=3). All measurements were taken at room temperature.

In an analogous approach to that of the synthesized rhodamine dyes, the photophysical properties of these dendrons were evaluated at different pH values (Table 2 and Figure 2). The spectral properties of fluorescein are known to be pH‐sensitive, especially due to the equilibrium between the mono‐ and dianion forms with a p*K*_a_ of 6.4,^[37]^ the dianion being the most fluorescent species.[^37^, ^38^] For this reason, measurements were taken at close‐to physiological (pH=7.3) and basic (pH=9.1) pH values to study the impact of this equilibrium in our family of fluorescein‐based fluorophores.

**Table 2:** Photophysical properties of fluorescein azide and fluorescein‐based dendritic fluorophores at different pH values.

	pH 7.3^[a]^				pH 9.1^[b]^			
Fluorophore	λ_abs_/λ_em_ [nm]	ϵ_max_^[c]^ [M^−1^ cm^−1^]	φ^[d]^	ϵ_max_ ⋅ φ [M^−1^cm^−1^]	λ_abs_/λ_em_ [nm]	ϵ_max_^[c]^ [M^−1^ cm^−1^]	φ^[d]^	ϵ_max_ ⋅ φ [M^−1^ cm^−1^]
**Fluorescein azide**	495/517	61000	0.77	46970	495/517	71000	0.91	64610
**FD2**	497/519	80000	0.13	10400	498/520	123000	0.26	31980
**FD4**	497/519	116000	0.04	4640	498 520	202000	0.05	10100
**FD8**	492/519	168000	<0.01	1176	498/519	375000	<0.01	2225
**RD_F2_D4**	532/555	33000	0.13	4290	537/558^[e]^	57000^[e]^	0.14^[e]^	7980^[e]^

[a] Measurements were taken in 10 mM HEPES, pH 7.3 buffer at room temperature. [b] Measurements were taken in 10 mM sodium borate, pH 9.1 buffer at room temperature. [c] Maximum molar extinction coefficients (ϵ_max_) were calculated by a linear regression analysis obeying the Beer‐Lambert law. [d] Quantum yields (φ) were determined using the comparative method and fluorescein (φ=0.91 in 0.1 M aqueous NaOH)^[36]^ as reference. [e] Measurements were taken in 0.1 % v/v TFA aqueous solution (pH=1.9) at room temperature. Quantum yields (φ) were determined using the comparative method and rhodamine 6G (φ=0.95 in EtOH)^[33]^ as reference.

As for the absorption properties, absorbance rose with the number of dyes. The maximum molar extinction coefficients (ϵ_max_) for **fluorescein azide**, **FD2**, **FD4** and **FD8** at pH 7.3 were 61000 (λ_max_=495 nm), 80000 (λ_max_=497 nm), 116000 (λ_max_=497 nm) and 168000 M^−1^ cm^−1^ (λ_max_=492 nm), respectively. Upon normalization, the ratio of the dyes’ respective ϵ_max_ values was therefore, 1/1.3/1.9/2.8. At pH 9.1, the fluorophores exhibited ϵ_max_ values of 71000 (λ_max_=495 nm), 123000 (λ_max_=498 nm), 202000 (λ_max_=498 nm) and 375000 M^−1^cm^−1^ (λ_max_=498 nm) for **fluorescein azide**, **FD2**, **FD4** and **FD8**, in that order. The ϵ_max_ ratio was, in this case, 1/1.8/2.9/5.4, showing an important pH‐dependent escalation of the absorption properties and approaching to the number of fluorescein units, that is 1 : 2 : 4 : 8.

Regarding the emission of the fluorophores, a decrease of the fluorescence quantum yield (φ) was observed along with the increasing number of fluorescein units (Table 2). More specifically, quantum yields at pH 7.3 for **fluorescein azide**, **FD2**, **FD4** and **FD8** were, in that order, 0.77, 0.13, 0.04 and <0.01. Analogously, the decay in quantum yield was observed at pH 9.1 although a wide variation as a function of pH was noted, particularly for **fluorescein azide** and **FD2**. Thus, the quantum yield values at pH 9.1 were 0.91, 0.26, 0.05 and <0.01 for **fluorescein azide**, **FD2**, **FD4** and **FD8**, respectively.

The fluorescence brightness (Table 2) diminished upon increasing the number of dyes per molecule at both pH values. Therefore, even though the absorption increased upon increasing the number of fluorescein moieties, the strong decay in quantum yield rendered a decrease in the fluorescence intensity. This behavior is confirmed by the fluorescence spectra shown in Figure 2. This decrease in fluorescence intensity was attributed to self‐quenching, presumably resulting from the relatively small Stokes shift of fluorescein and the proximity of the dyes within the dendritic structures.[^9^, ^38^, ^39^] It is noteworthy to mention the bimodal shape of the absorbance spectrum of **FD8** at pH 7.3. In comparison to the other analogous dendrons, **FD8** exhibited a hypsochromic shift in which a second absorption band arose at 462 nm. This was suggestive of the formation of H‐aggregates caused by π–π interactions between the different dye units.[^23^, ^40^, ^41^, ^42^]. These interactions were less prevalent at pH 9.1 with the more polar and water‐soluble dianion form of fluorescein.[^43^, ^44^] Hence, the absorption spectrum recovered the original pattern.

**Figure 3:**
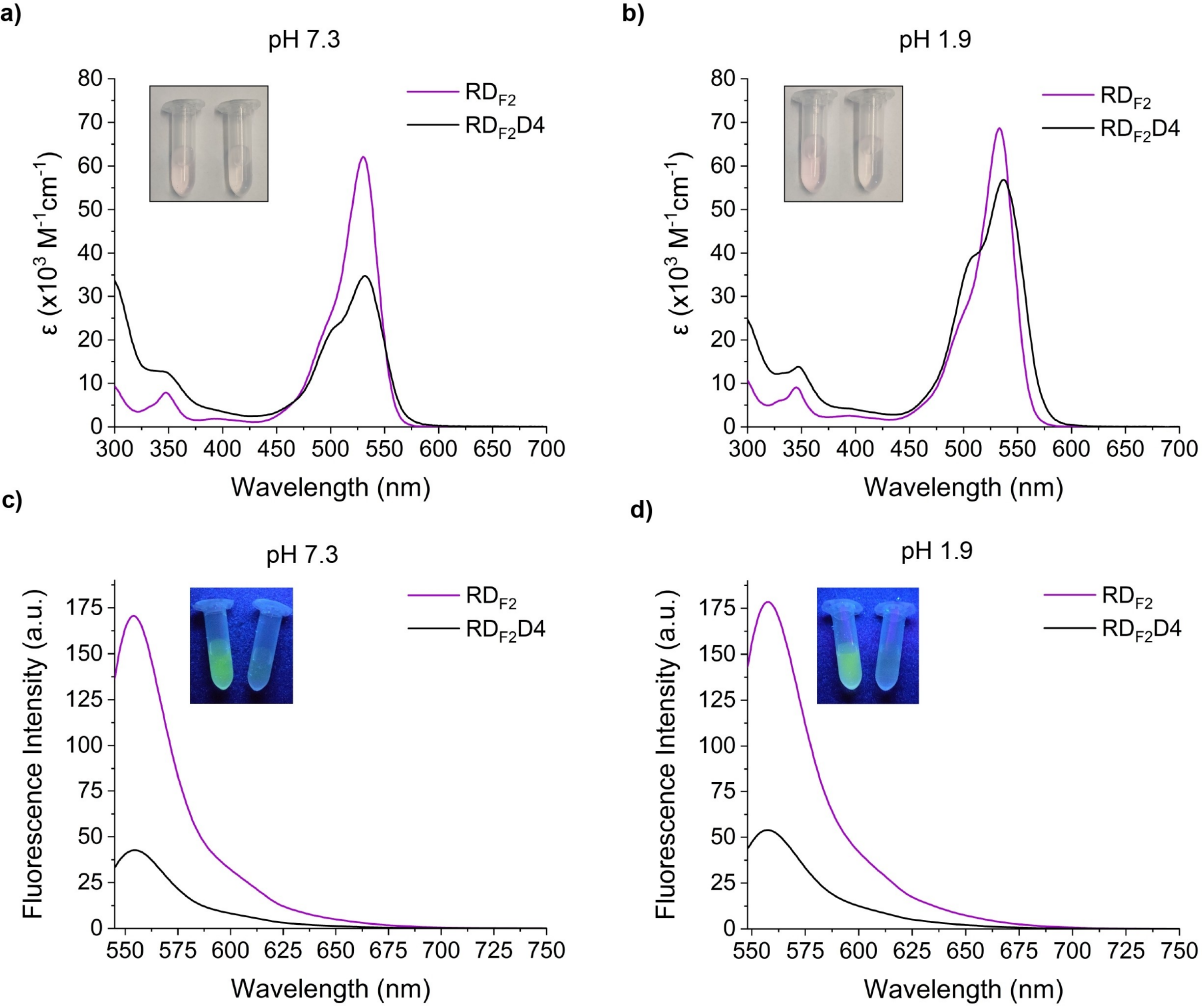
Absorption and emission spectra of RD_F2_ and RD_F2_D4 at different pH values. a) Absorption spectra measured in 10 mM HEPES, pH 7.3 buffer at a concentration of 2 μM. b) Absorption spectra measured in 0.1 % v/v TFA aqueous solution (pH=1.9) at a concentration of 2 μM. c) Emission spectra measured in 10 mM HEPES, pH 7.3 buffer at a concentration of 0.2 μM. λ_ex_=530 nm (RD_F2_) and 532 nm (RD_F2_D4). d) Emission spectra measured in 0.1 % v/v TFA aqueous solution (pH=1.9) at a concentration of 0.2 μM. λ_ex_=533 nm (RD_F2_) and 537 nm (RD_F2_D4). Inset: Solutions in the photographs correspond, from left to right, to RD_F2_ and RD_F2_D4 at 2 μM (absorbance) or 0.2 μM (emission). Absorbance and emission scans are averages (n=3). All measurements were taken at room temperature.

After evaluating the fluorescein‐based family of dendritic fluorophores, we concluded that the macromolecules are ill‐suited for use in nucleic acid detection compared with their individual monomeric substituents. The strong interactions between the different dye units led to self‐quenching, which was evidently undesirable for the synthesis of dendron‐based fluorescence amplifiers.

Raddaoui, Stazzoni et al.^[24]^ developed a TAMRA‐functionalized dendritic fluorophore which was bioconjugated to EdU‐labelled DNA via click chemistry and achieved fluorescence amplification. Inspired by this work, we focused our efforts on the preparation of a rhodamine‐based dendritic fluorophore using our model dye **RD_F2_**. In the event, after functionalizing an alkyne‐presenting bis‐MPA dendron via copper‐catalyzed click chemistry, **RD_F2_D4** was afforded as a bright, pink powder in 85 % yield (Scheme 2). Once the photophysical properties were measured (Table 2 and Figure 3), **RD_F2_D4** was found to exhibit absorption and emission wavelengths very similar to **RD_F2_** at both pH 7.3 and pH 1.9. As for the extinction coefficient, lower values than those of **RD_F2_** were noted as well as an important pH‐dependence (ϵ_max_=33000 M^−1^ cm^−1^ at pH 7.3 and ϵ_max_=57000 M^−1^cm^−1^ at pH 1.9). In terms of quantum yield, an important decay of the fluorescence emission was observed with a negligible effect of pH (φ=0.13 at pH 7.3 and φ=0.14 at pH 1.9). Therefore, as shown in Figure 3, the fluorescence intensity of **RD_F2_D4** did not surpass that of **RD_F2_**. The most plausible explanation of this behavior is, once again, the self‐quenching caused by the relatively small Stokes shift of **RD_F2_** as well as the relative proximity of the dye units within the dendron. A tendency of **RD_F2_D4** to form H‐aggregates in aqueous solution is also observed by the presence, at both pH values, of an additional shoulder at 505 nm (pH=7.3) or 510 nm (pH=1.9). The suitability of **RD_F2_D4** as a fluorescent amplifier is, thus, limited by its inherent self‐quenching.

**Figure 4:**
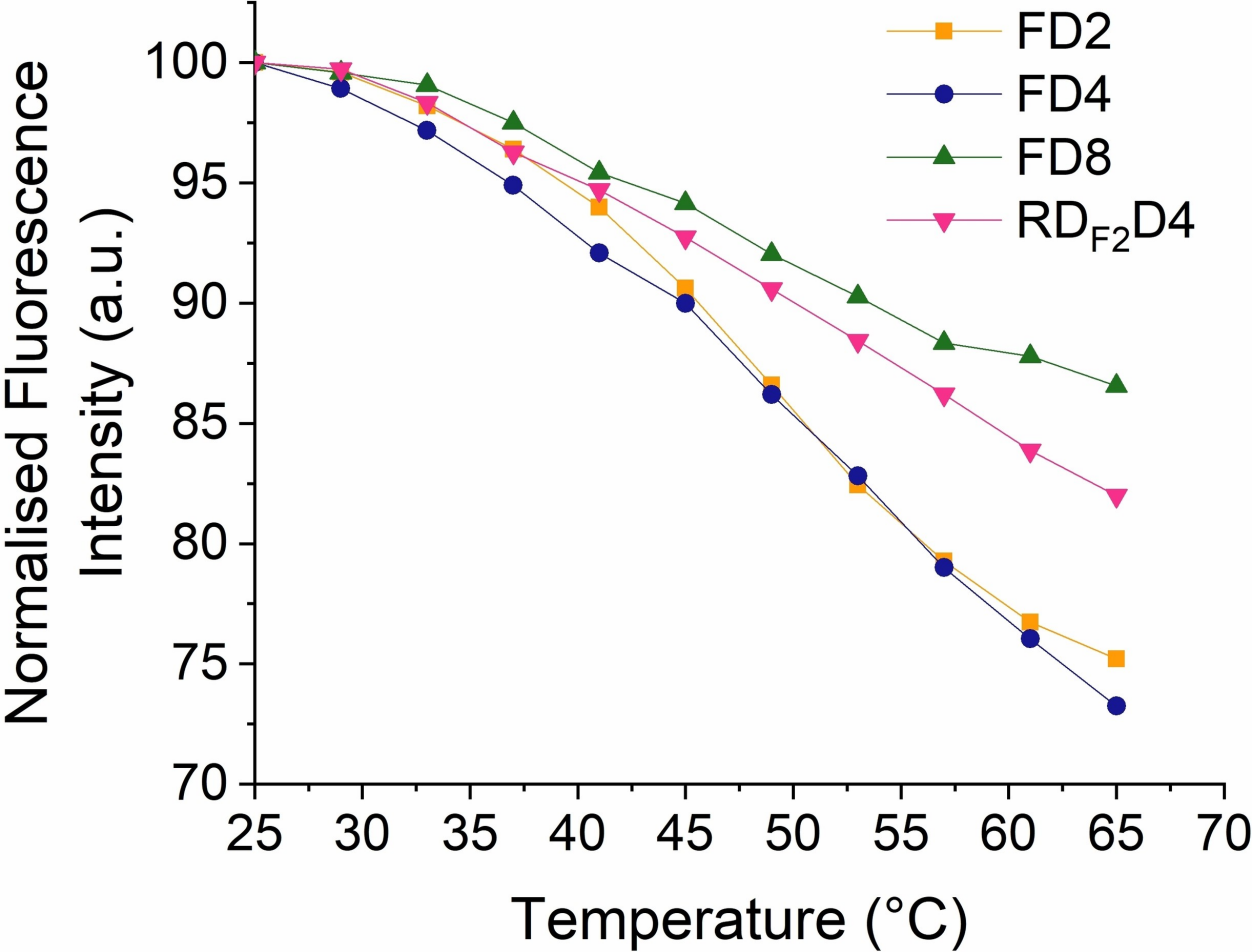
Effect of temperature on the fluorescence intensity of the synthesized dendritic fluorophores.

Previous work has demonstrated aggregates tend to diminish upon temperature elevation due to changes in the dynamic equilibrium, and thus resulting in an increase of fluorescence intensity.−–^[45−–47]^ Conversely, non‐radiative rate constants and collisional quenching also tend to be amplified upon heating, leading to a decrease of fluorescence.^[48]^ In order to evaluate which of the two effects predominate in our dendritic fluorophores, the fluorescence of these molecules was measured at varying temperature values between 25 °C and 65 °C, as shown in Figure 4. In the event, the fluorescein‐based dendrons **FD2** and **FD4** exhibited comparable fluorescence decay behavior. **FD8**, meanwhile, showed a diminished change in fluorescence intensity upon heating compared with **FD2** and **FD4**. This behavior could be indicative of a reconversion of aggregates to monomers in solution. For **RD_F2_D4**, a less pronounced decay of fluorescence is shown in comparison to its fluorescein analogue **FD4**, indicating that aggregation might occur at a lesser extent for the former compound at higher temperatures. Overall, it is apparent that non‐radiative processes and collisional quenching prevail for all fluorophores upon increase of temperature, leading to a general decay in their fluorescence intensity.

## Conclusion

The improvement and development of new dyes is of utmost importance for fluorescence‐based diagnostic advancements. In cell proliferation assays, high signal‐to‐noise ratios allow reliable detection of smaller number of proliferating cells. To achieve these requirements, fluorophores with enhanced fluorescent properties must be identified, synthesized, and introduced into biomolecules without jeopardizing their biological activity.

In this work, the successful development of a direct, divergent pathway for the synthesis of *ready‐to‐click* rhodamine dyes afforded three fluorophores, namely, **RD_H2_**, **RD_m_** and **RD_F2_**, showing remarkable photophysical properties, low pH‐dependence, and high potential as fluorescent probes in EdU cell proliferation assays. The development of this new synthetic pathway, therefore, proves as a promising strategy for the rapid development of new click chemistry‐conjugable fluorophores.

To further increase the fluorescence signal of our fluorophores, a family of three bis‐MPA dendritic scaffolds containing 2, 4 and 8 alkyne functionalities were decorated via click chemistry. In this case, **fluorescein azide** was used as a standard fluorophore to afford **FD2**, **FD4**, and **FD8**, respectively. As for the photophysical properties, which were found to be highly pH‐dependent, an increase of the maximum extinction coefficient along with the number of dye units was observed. In contrast, the fluorophores showed a decrease of quantum yield together with the increasing number of dye moieties which, in turn, caused a decay of the fluorescence intensity. We decided then to use our model rhodamine dye **RD_F2_** to prepare **RD_F2_D4**. This latter molecule showed lower values of both extinction coefficient and quantum yield in comparison to **RD_F2_** and, thus, a decay of the fluorescence intensity. Ultimately, the effect of temperature on fluorescence intensity of the dendritic fluorophores was evaluated, leading to a general decay of fluorescence. This behavior suggests that collisional quenching prevails over aggregate dissociation upon temperature increase.

Albeit the observed self‐quenching of fluorescence was not favorable in this study, comprehensive insights of the behavior of these dye‐functionalized dendrons were acquired. It is apparent that the development of these dendritic structures remains challenging[^9^, ^21^, ^22^] and further optimization must be carried out. Relatively high lipophilicity of the employed fluorophores appears to be the main reason for the observed self‐quenching. Introduction of water‐soluble functionalities into the dye moieties would reduce closeness between them, elude aggregation and possibly enable amplification of the fluorescent signal. Nevertheless, the strongly absorbing and nonfluorescent properties of these dendrons could serve in other applications as quenchers for Förster resonance energy transfer (FRET) experiments.^[49]^

## Experimental Section

**Materials and methods** for chemical synthesis and full characterization of all new compounds are found in the Supporting Information.

**UV‐vis and fluorescence spectroscopy**: Samples for spectroscopy were prepared as stock solutions in DMSO and diluted such that the DMSO concentration did not exceed 1 % (v/v). Absorption spectra were recorded on an Agilent Cary 50 spectrometer. Fluorescence spectra were recorded on a Shimadzu RF‐5301PC fluorescence spectrometer. All measurements were performed at ambient temperature using 1 cm path length, 3.5 mL quartz cuvettes from Hellma Analytics. Absorbance/extinction coefficient and emission scans are averages (n=3).

**Maximum extinction coefficient determination**: All reported maximum extinction coefficients (ϵ_max_) were calculated by a linear regression analysis obeying the Beer‐Lambert law. Measurements were carried out using moderately concentrated samples (*A*<0.8) to ensure linearity between absorbance and concentration. All absorbance values are averages (*n*=3). Plots are found in the Supporting Information.

**Quantum yield determination**: All reported fluorescence quantum yield values (φ) were determined using the comparative method.^[50]^ Fluorescein (φ=0.91 in 0.1 M aqueous NaOH)^[36]^ and rhodamine 6G (φ=0.95 in EtOH)^[28]^ were used as references for fluorescein‐ and rhodamine‐based fluorophores, respectively. Reported refractive index values were taken for the aqueous solutions and buffers[^51^, ^52^] (n=1.33) and EtOH^[53]^ (n=1.36). Measurements were carried out using dilute samples (*A*<0.1) to minimize inner filter effects.^[54]^ All absorbance and integrated fluorescence intensity (fluorescence area) values are averages (n=3). The slopes of the plots of fluorescence area vs. absorbance were used in the comparative method by means of the following equation:

$$\varphi_{s}=\varphi_{r}\cdot \frac{m_{s}}{m_{r}}\cdot {\left(\frac{n_{s}}{n_{r}}\right)}^{2}$$

in which φ=fluorescence quantum yield, m=slope of the plot of fluorescence area vs. absorbance, n=refractive index and *r* and *s* subscripts refer to the reference and unknown fluorophore, respectively. Plots are found in the Supporting Information.

**EdU cell proliferation assay**: The EdU Cell Proliferation Kit for imaging EdU‐Click 555 (baseclick GmbH) was used following the manufacturer's instruction with minor modifications. HEK‐293T cells were seeded on No. 1 glass coverslips and grown in DMEM containing 10 % FBS for 24 h before being pulsed for 2 h with 10 μM EdU in the same growth medium. After washing with PBS, the cells were fixed with 4 % formaldehyde in PBS for 15 min, permeabilized for 15 min with 0.1 % saponin in PBS containing 1 % BSA and incubated for 30 min in a cocktail containing 2 μM dye‐azide (either **RD_F2_** or the 5‐TAMRA‐PEG3‐Azide provided with the kit), 1x Reaction buffer, 1x Buffer additive and Catalyst solution. Coverslips were then washed 3 times for 5 min with PBS containing 0.1 % saponin and 1 % BSA with inclusion of DAPI (1 : 15 dilution of NucBlue Fixed Cell ReadyProbes Reagent, Life Technologies) in the first wash. Finally, the coverslips were mounted with Fluoroshield mounting medium (Sigma) on glass slides and imaged using an EVOS FL II fluorescent microscope equipped with an oil immersion 100x Plan Fluorite objective and EVOS LED Cubes RFP (531/40 nm excitation, 593/40 nm emission) and DAPI (357/44 excitation, 447/60 nm; all from Life Technologies). Identical illumination intensity and exposure time were used for imaging samples treated with the two dye‐azides. Images were processed using the ImageJ software.

## Conflict of interest

The authors declare no conflict of interest.

1

## Supporting information

As a service to our authors and readers, this journal provides supporting information supplied by the authors. Such materials are peer reviewed and may be re‐organized for online delivery, but are not copy‐edited or typeset. Technical support issues arising from supporting information (other than missing files) should be addressed to the authors.

Supporting Information

## Data Availability

The data that support the findings of this study are available from the corresponding author upon reasonable request.

## References

[1] N. Nath , B. Godat , M. Urh , J. Visualization 2016, 2016, 54545.

[2] R. W. Dirks , H. J. Tanke , BioTechniques 2006, 40, 489–496. 16629396 10.2144/000112121

[3] O. Roger , S. Colliec-Jouault , J. Ratiskol , C. Sinquin , J. Guezennec , A. M. Fischer , L. Chevolot , Carbohydr. Polym. 2002, 50, 273–278.

[4] C. Schultz , A. B. Neef , T. W. Gadella , J. Goedhart , Cold Spring Harb. Protoc. 2010, 5, 10.1101/pdb.prot5459. 20647362

[5] D. Maity , J. Jiang , M. Ehlers , J. Wu , C. Schmuck , Chem. Commun. 2016, 52, 6134–6137. 10.1039/c6cc02138g 27071707

[6] A. Nagy , E. Vitásková , L. Černíková , V. Křivda , H. Jiřincová , K. Sedlák , J. Horníčková , M. Havlíčková , Sci. Rep. 2017, 7, 1–10. 28120891 10.1038/srep41392 PMC5264587

[7] Y. Huang , D. Kong , Y. Yang , R. Niu , H. Shen , H. Mi , Biotechnol. Lett. 2004, 26, 891–895. 15269536 10.1023/b:bile.0000025898.15309.0e

[8] B. Oberleitner , A. Manetto , T. Frischmuth , BIOspektrum 2014, 20, 188–190.

[9] C. Wängler , G. Moldenhauer , R. Saffrich , E. M. Knapp , B. Beijer , M. Schnölzer , B. Wängler , M. Eisenhut , U. Haberkorn , W. Mier , Chem. Eur. J. 2008, 14, 8116–8130. 18752247 10.1002/chem.200800328

[10] S. Nik-Zainal , P. Van Loo , D. C. Wedge , L. B. Alexandrov , C. D. Greenman , K. W. Lau , K. Raine , D. Jones , J. Marshall , M. Ramakrishna , A. Shlien , S. L. Cooke , J. Hinton , A. Menzies , L. A. Stebbings , C. Leroy , M. Jia , R. Rance , L. J. Mudie , S. J. Gamble , P. J. Stephens , S. McLaren , P. S. Tarpey , E. Papaemmanuil , H. R. Davies , I. Varela , D. J. McBride , G. R. Bignell , K. Leung , A. P. Butler , J. W. Teague , S. Martin , G. Jönsson , O. Mariani , S. Boyault , P. Miron , A. Fatima , A. Langerod , S. A. J. R. Aparicio , A. Tutt , A. M. Sieuwerts , Å. Borg , G. Thomas , A. V. Salomon , A. L. Richardson , A. L. Borresen-Dale , P. A. Futreal , M. R. Stratton , P. J. Campbell , Cell 2012, 149, 994–1007. 22608083 10.1016/j.cell.2012.04.023 PMC3428864

[11] S. Vira , E. Mekhedov , G. Humphrey , P. S. Blank , Anal. Biochem. 2010, 402, 146–150. 20362543 10.1016/j.ab.2010.03.036 PMC2876214

[12] C. P. Toseland , J. Chem. Biol. 2013, 6, 85–95. 24432126 10.1007/s12154-013-0094-5 PMC3691395

[13] J. B. Grimm , L. D. Lavis , Nat. Methods 2021, 19, 149–158. 34949811 10.1038/s41592-021-01338-6

[14] A. Salic , T. J. Mitchison , Proc. Nat. Acad. Sci. 2008, 105, 2415–2420. 18272492 10.1073/pnas.0712168105 PMC2268151

[15] J. Gierlich , G. A. Burley , P. M. E. Gramlich , D. M. Hammond , T. Carell , Org. Lett. 2006, 8, 3639–3642. 16898780 10.1021/ol0610946

[16] G. M. Solius , D. I. Maltsev , V. V. Belousov , O. V. Podgorny , J. Biol. Chem. 2021, 297, 101345. 34717955 10.1016/j.jbc.2021.101345 PMC8592869

[17] A. Carlmark , E. Malmström , M. Malkoch , Chem. Soc. Rev. 2013, 42, 5858–5879. 23628841 10.1039/c3cs60101c

[18] S. S. Santos , R. V. Gonzaga , J. V. Silva , D. F. Savino , D. Prieto , J. M. Shikay , R. S. Silva , L. H. A. Paulo , E. I. Ferreira , J. Giarolla , Can. J. Chem. 2017, 95, 907–916.

[19] X. Yan , Y. Yang , Y. Sun , J. Phys. Conf. Ser. 2021, 1948, 012205.

[20] S. K. Prajapati , S. D. Maurya , M. K. Das , V. K. Tilak , K. K. Verma , R. C. Dhakar , J. Drug Deliv. Ther. 2016, 6, 67–92.

[21] J. Manono , C. A. Dougherty , K. Jones , J. DeMuth , M. M. B. Holl , S. DiMaggio , Mater. Today Commun. 2015, 4, 86–92. 10.1016/j.mtcomm.2015.06.006 PMC4631223 26549978

[22] C. A. Dougherty , S. Vaidyanathan , B. G. Orr , M. M. Banaszak Holl , Bioconjugate Chem. 2015, 26, 304–315. 10.1021/bc5005735 PMC4636191 25625297

[23] Á. Martín-Serrano Ortiz , P. Stenström , P. Mesa Antunez , O. C. J. Andrén , M. J. Torres , M. I. Montañez , M. Malkoch , J. Polym. Sci. Part A 2018, 56, 1609–1616.

[24] N. Raddaoui , S. Stazzoni , L. Möckl , B. Viverge , F. Geiger , H. Engelke , C. Bräuchle , T. Carell , ChemBioChem 2017, 18, 1716–1720. 28640486 10.1002/cbic.201700209

[25] M. Ceresole, Verfahren Zur Darstellung von Farbstoffen Aus Der Gruppe Des Meta-Amidophenolphtaleïns. German Patent 44002, **1887**.

[26] M. Beija , C. A. M. Afonso , J. M. G. Martinho , Chem. Soc. Rev. 2009, 38, 2410–2433. 19623358 10.1039/b901612k

[27] L. D. Lavis , R. T. Raines , ACS Chem. Biol. 2008, 3, 142–155. 18355003 10.1021/cb700248m PMC2802578

[28] J. B. Grimm , A. K. Muthusamy , Y. Liang , T. A. Brown , W. C. Lemon , R. Patel , R. Lu , J. J. Macklin , P. J. Keller , N. Ji , L. D. Lavis , Nat. Methods 2017, 14, 987–994. 28869757 10.1038/nmeth.4403 PMC5621985

[29] L. D. Lavis , R. T. Raines , ACS Chem. Biol. 2014, 9, 855–866. 24579725 10.1021/cb500078u PMC4006396

[30] J. B. Grimm , L. D. Lavis , Org. Lett. 2011, 13, 6354–6357. 22091952 10.1021/ol202618t PMC3235915

[31] J. B. Grimm , B. P. English , J. Chen , J. P. Slaughter , Z. Zhang , A. Revyakin , R. Patel , J. J. Macklin , D. Normanno , R. H. Singer , T. Lionnet , L. D. Lavis , Nat. Methods 2015, 12, 244–250. 25599551 10.1038/nmeth.3256 PMC4344395

[32] T. Peng , D. Yang , Org. Lett. 2010, 12, 496–499. 20067265 10.1021/ol902706b

[33] D. Magde , R. Wong , P. G. Seybold , Photochem. Photobiol. 2002, 75, 327–334. 12003120 10.1562/0031-8655(2002)075<0327:fqyatr>2.0.co;2

[34] L. Wu , K. Burgess , J. Org. Chem. 2008, 73, 8711–8718. 18928318 10.1021/jo800902j

[35] T. G. Hwang , G. R. Han , J. M. Lee , J. W. Lee , H. M. Kim , D. Hwang , S. K. Kim , J. P. Kim , J. Phys. Chem. C 2019, 123, 24263–24274.

[36] L. Porrès , A. Holland , L. O. Pålsson , A. P. Monkman , C. Kemp , A. Beeby , J. Fluoresc. 2006, 16, 267–273. 16477506 10.1007/s10895-005-0054-8

[37] R. Sjöback , J. Nygren , M. Kubista , Spectrochim. Acta Part A 1995, 51, L7–L21.

[38] J. R. Lakowicz , Principles of Fluorescence Spectroscopy, Springer, 2006.

[39] U. Mahmood , R. Weissleder , Mol. Cancer Ther. 2003, 2, 489–496. 12748311

[40] A. Eisfeld , J. S. Briggs , Chem. Phys. 2006, 324, 376–384.

[41] N. J. Hestand , F. C. Spano , Chem. Rev. 2018, 118, 7069–7163. 29664617 10.1021/acs.chemrev.7b00581

[42] I. L. Arbeloa , J. Chem. Soc. Faraday Trans. 2 Mol. Chem. Phys. 1981, 77, 1725–1733.

[43] M. Ogawa , N. Kosaka , P. L. Choyke , H. Kobayashi , ACS Chem. Biol. 2009, 4, 535–546. 19480464 10.1021/cb900089j PMC2743556

[44] M. Sun , K. Müllen , M. Yin , Chem. Soc. Rev. 2016, 45, 1513–1528. 26797049 10.1039/c5cs00754b

[45] A. Hassanzadeh , A. Zeini-Isfahani , M. H. Habibi , Spectrochim. Acta Part A 2006, 64, 464–476. 10.1016/j.saa.2005.07.077 16644265

[46] “ATTO-TEC GmbH – Dye-Aggregation,” can be found under https://www.atto-tec.com/fluorescence/dye-aggregation/?language=en, **2021**.

[47] W. C. Lai , N. S. Dixit , R. A. Mackay , J. Phys. Chem. 1984, 88, 5364–5368.

[48] H. R. Deepa , J. Thipperudrappa , H. M. S. Kumar , J. Phys. Conf. Ser. 2020, 1473, 012046.

[49] L. Wu , C. Huang , B. P. Emery , A. C. Sedgwick , S. D. Bull , X. P. He , H. Tian , J. Yoon , J. L. Sessler , T. D. James , Chem. Soc. Rev. 2020, 49, 5110–5139. 32697225 10.1039/c9cs00318e PMC7408345

[50] K. Lawson-Wood , S. Upstone , E. Kieran , Fluoresc. Spectrosc. 2018, 4, 1–5.

[51] Z. C. Yang , M. Wang , A. M. Yong , S. Y. Wong , X. H. Zhang , H. Tan , A. Y. Chang , X. Li , J. Wang , Chem. Commun. 2011, 47, 11615–11617. 10.1039/c1cc14860e 21931886

[52] M. M. Koerner , L. A. Palacio , J. W. Wright , K. S. Schweitzer , B. D. Ray , H. I. Petrache , Biophys. J. 2011, 101, 362–369. 21767488 10.1016/j.bpj.2011.05.062 PMC3136761

[53] J. Rheims , J. Köser , T. Wriedt , Meas. Sci. Technol. 1997, 8, 601.

[54] S. Dhami , A. J. D. Mello , G. Rumbles , S. M. Bishop , D. Phillips , A. Beeby , Photochem. Photobiol. 1995, 61, 341–346.

