# Supplementary material for: Divergent Synthesis of Ultrabright and Dendritic Xanthenes for Enhanced Click‐Chemistry‐Based Bioimaging
Source: Chemistry. 2022 Dec 7;29(5):e202202633. doi: 10.1002/chem.202202633 (PMC10107433; doi:10.1002/chem.202202633)
Supplement: Supplementary file 1 — Supporting Information [file CHEM-29-0-s001.pdf]

Chemistry–A European Journal

Supporting Information

Divergent Synthesis of Ultrabright and Dendritic Xanthenes for Enhanced Click-Chemistry-Based Bioimaging

Luis Montiel, Fabio Spada, Antony Crisp, Sascha Serdjukow, Thomas Carell, and Thomas Frischmuth*

Table of Contents

1. General Experimental Methods 2

2. Synthetic Procedures 5

3. Photophysical Properties of the Synthesised Fluorophores 16

4. Photostability Studies of RD_{H2}, RD_m and RD_{F2} 25

5. pK_a Values Calculation of RD_{H2}, RD_m and RD_{F2} 27

6. Solution Stability Studies 29

7. Mass Spectra of the Synthesised Compounds 30

8. NMR Spectra of the Synthesised Compounds 33

9. References 45

10. Author Contributions 45

1. General Experimental Methods

Materials and methods. Chemicals were purchased from Sigma-Aldrich, TCI, Fluka, ABCR, Acros Organics, VWR, Polymer Factory or Sapala Organics and were used without further purification. Solvents were purchased in septum-sealed bottles stored under an inert atmosphere. All reactions were magnetically stirred under a positive pressure of Argon (Ar) unless otherwise stated. Reactions and chromatography fractions were monitored by qualitative thin-layer chromatography (TLC) on silica gel F254 TLC plates from Merck KGaA, visualised by UV illumination or developed with ninhydrin, ceric ammonium molybdate or KMnO4 stains. Flash column chromatography was performed using silica gel (40-63 µm) or basic alumina (~150 mesh) from Merck KGaA. Size exclusion chromatography was performed on Sephadex® LH-20 (18-111 µm) from Merck KGaA. Dialysis membranes Spectra/Por® 6 MWCO 1 KDa (18 mm flat width) and Spectra/Por® 6 MWCO 3.5 KDa (18 mm flat width) were purchased from Repligen and used as received.

Nuclear Magnetic Resonance (NMR). Spectra were recorded on a *Bruker Avance III HD 400* (400 MHz), *Varian NMR-System 600* (600 MHz) and *Bruker Avance III HD* with *Cryo-Kopf 800* (800 MHz) spectrometers. ^{1}H chemical shifts were internally calibrated to the residual protons of the deuterated solvent: DMSO-d_5 (2.50 ppm) and CD_2HOD (3.31 ppm). ^{13}C NMR shifts were calibrated to the residual solvent: DMSO-d_6 (39.52 ppm), CD_3OD (49.00 ppm). ^{19}F chemical shifts (δ) were referenced to an external reference: $CFCl_3$ (0.00 ppm) or CF_3COOH (-76.55 ppm). Data for ^{1}H NMR spectra are reported as follows: chemical shift (δ ppm), multiplicity (s = singlet, d = doublet, t = triplet, q = quartet, p = pentuplet, dd = doublet of doublets, m = multiplet, br = broad), coupling constant (Hz), integration. Data for proton-decoupled ^{13}C and ^{19}F NMR spectra are reported by chemical shift (δ ppm) and, where necessary, multiplicity (s = singlet, t = triplet, q = quartet). All NMR spectra were analysed using the software MestreNova 14.1.1 from Mestrelab Research S. L. Broadening of aromatic signals was observed in the ^{1}H and ^{13}C NMR spectra of the synthesised dendritic dyes, most likely due to strong π-π stacking between the dye moieties, as previously reported by Parenti *et al.*[1]

IR spectroscopy. IR spectra were recorded on a PerkinElmer Spectrum BX II FT-IR system. All substances were directly applied as solids or on the ATR unit.

Mass Spectrometry (MS). High resolution mass spectra (ESI-MS) were recorded by the analytical section of the Department of Chemistry of the Ludwig-Maximilians-Universität München on a spectrometer MAT 90 (ESI) from Thermo Finnigan GmbH. Raw data was extracted using OpenChrome Lablicate Edition version 1.5.0, and spectra were processed in Origin 2018. Theoretical spectra were calculated using the Isotope Distributor Calculator and Mass Spec Plotter by Adaptas Solutions (https://www.sisweb.com/mstools/isotope.htm) and processed in Origin 2018. Matrix-assisted laser

desorption/ionization-time-of-flight (MALDI-TOF) mass spectra were recorded on a Bruker Autoflex II. The instrument was calibrated using SpheriCal™ calibrants. Samples were prepared by mixing 5 µL of 1 mg/mL analyte solution in EtOAc or MeOH, 5 µL of a 1 mg/mL counterion solution of sodium trifluoroacetate (NaTFA) in tetrahydrofuran (THF) and 20 µL of a 10 mg/mL trans-2-[3-(4-tert-butylphenyl)-2-methyl-2-propenylidene]-malononitrile (DCTB) solution in THF. 1 µL of the final mixture was applied to a stainless-steel sample plate using the dried droplet method. The obtained spectra were analysed with FlexAnalysis version 2.2 from Bruker Daltonics and processed in Origin 2018.

Analytical and preparative HPLC. Analytical RP-HPLC was performed on a Waters Alliance (e2695 Separation Module, 2998 Photodiode Array Detector) instrument equipped with an XBridge™ OST C18 column (2.5µm, 4.6mm x 50mm) using a flow of 1.5 mL/min at 40 °C column temperature. A linear gradient of buffer B 0-30 % v/v from 0 → 4 min and then 30-85 % v/v from 4 → 10 min was applied. Buffer A: 0.1 M triethylammonium acetate in H_2O. Buffer B: 0.1 M triethylammonium acetate in MeCN/H_2O 8:2 v/v. Preparative RP-HPLC was performed on a Waters Breeze (2487 Dual λ Array Detector, 1525 Binary HPLC Pump) instrument equipped with the column VP 250/32 C18 from Macherey Nagel using a flow of 5 mL/min at room temperature. Compounds were detected at the wavelength of maximum absorption (λ_{max}) of the corresponding dyes. The employed gradients for the subsequent purifications are specified in the Synthetic Procedures section.

Purification of dendrons by dialysis. High molecular weight dendrons were unsuitable for conventional flash chromatography and were instead purified according to the following procedure. A solution containing a particular dendron and other reaction components in DMSO (1 mL) was transferred to a dialysis membrane (ca. 10 cm of tubing) and carefully sealed at each end with a conventional clamp. The filled membrane was subsequently placed a beaker containing 1.0 L of DMSO and gently stirred for the reported time. The outer dialysate was replaced with fresh DMSO 2-3 times throughout the specified time course. The dialysis bag was then placed in a beaker containing 1.0 L H_2O for the specified time to remove the majority DMSO from the inner compartment by diffusion.

Visualisation of spectroscopic data in Origin. Absorption spectra, emission spectra, calibration curves, and their regression line equations were plotted and calculated using the software package Origin 2018 according to the developers' instructions.

Photostability measurements. A VFL-P-532 and a VFL-P-560 lasers from MPB communications were utilised to irradiate the samples at 532 nm and 560 nm, respectively. An *i*BEAM SMART 488 laser from TOPTICA was utilised to irradiate the samples at 488 nm.

The relationship between relative fluorescence intensity and irradiation time satisfies **Equation 1**:

$$\ln\left(\frac{I}{I_0}\right) = -k \cdot t \qquad \text{(Equation 1)}$$

Where I = fluorescence intensity at a time t, I_0 = initial fluorescence intensity, k = photobleaching rate constant and t = irradiation time. The slope's absolute value of the linear fitting between $ln\left(\frac{I}{I_0}\right)$ and t corresponds to the photobleaching rate constant (k). The fluorescence half-life time ($t_{1/2}$), therefore, was obtained following **Equation 2**:

$$t_{1/2} = \frac{\ln 2}{k} \qquad \text{(Equation 2)}$$

Calculation of pK_a values with Marvin. The software package Marvin (version 21.17.0, ChemAxon) was used for calculating the pK_a values of **RD_{H2}**, **RD_m** and **RD_{F2}** (https://www.chemaxon.com).

Nomenclature of synthesised fluorophores. Rhodamine dyes are named following the structure RD_X, where *RD* stands for rhodamine dye and *X* the substituent of the azetidine moiety (e.g. RD_{F2} corresponds to the rhodamine dye containing 4,4-difluoroazetidine groups). Fluorescein-based dendrons are abbreviated as FDn, being *FD* fluorescein dendron and *n* the number of fluorescein substituents present in the dendritic scaffold (e.g. FD4 corresponds to the dendron containing 4 fluorescein units). Rhodamine-based dendrons are named following the structure (RD_x)Dn, where RD_x is the type of rhodamine dye found in the scaffold, *D* stands for dendron, and *n* is the number of dye copies (e.g. RD_{F2}D4 corresponds to the dendron containing 4 units of RD_{F2}).

2. Synthetic Procedures

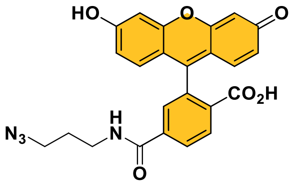

8
Chemical Formula: $C_{24}H_{18}N_4O_6$
Molecular Weight: 458.43

Fluorescein azide (8). A magnetically-stirred solution containing 6-carboxyfluorescein (**1**) (200 mg, 0.53 mmol, 1 eq) and *N,N'*-disuccinimidyl carbonate (DSC) (300 mg, 1.17 mmol, 2.2 eq) in DMF (8 mL) was treated with triethylamine (0.45 mL, 3.19 mmol, 6 eq) and then 4-dimethylaminopyiridine (DMAP) (6.49 mg, 0.053 mmol, 0.1 eq), and the resulting mixture was maintained at room temperature for 1 h while shielded from light. After this time, 3-azidopropan-1-amine (133 mg, 1.33 mmol, 2.5 eq) was added and the reaction stirred an additional 2 h at room temperature. The crude product was subsequently concentrated *in vacuo*, dissolved in EtOAc (20 mL), and washed with aqueous $KHSO_4$ (1 M, 2 x 10 mL) and brine (10 mL). The combined organic extracts were dried over Na_2SO_4, filtered, and concentrated *in vacuo*. Purification by silica gel flash chromatography (9:1 CH_2Cl_2/MeOH v/v) afforded compound **8** (171 mg, 0.37 mmol, 70 %) as an orange solid.

$\mathbf{R}_f$ = 0.4 (SiO_2; CH_2Cl_2/MeOH, 9:1). **^{1}H NMR** (600 MHz, DMSO-*d*$_6$) δ 10.17 (br, 2H), 8.74 (t, *J* = 5.6 Hz, 1H), 8.17 (dd, *J* = 8.0, 1.4 Hz, 1H), 8.08 (d, *J* = 8.0 Hz, 1H), 7.67 (d, *J* = 1.3 Hz, 1H), 6.71 (d, *J* = 2.2 Hz, 2H), 6.59 (d, *J* = 8.7 Hz, 2H), 6.56 (dd, *J* = 8.7, 2.3 Hz, 2H), 3.35 (t, *J* = 6.7 Hz, 2H), 3.26 (app. q, *J* = 6.5 Hz, 2H), 1.72 (app. p, *J* = 6.8 Hz, 2H). **^{13}C NMR** (151 MHz, DMSO-*d*$_6$) δ 168.2, 164.7, 159.8, 152.7, 151.9, 140.7, 129.5, 129.4, 128.3, 125.0, 122.3, 112.9, 109.2, 102.3, 83.6, 48.5, 36.9, 28.2. **IR** (ATR): $\tilde{\nu}$ (cm^{-1}) = 3326 (br), 3148 (br), 2100 (s), 1704 (vs), 1644 (m), 1631 (m), 1612 (s) 1588 (s), 1554 (s), 1505 (s), 1463 (s), 1363 (m), 1342 (m), 1318 (w), 1292 (w), 1273 (m), 1248 (vs), 1232 (vs), 1191 (vs), 1176 (s), 1149 (s), 1108 (s), 1014 (w), 998 (m), 977 (w), 950 (w), 925 (w), 852 (s), 838 (s), 797 (m), 753 (m), 717 (w), 691 (m), 672 (m). **HRMS** (ESI): *m/z* calcd for $C_{24}H_{19}N_4O_6^+$ $[M+H]^+$: 459.1305, found: 459.1299.

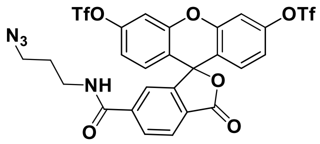

9

Chemical Formula: $C_{26}H_{16}F_6N_4O_{10}S_2$
Molecular Weight: 722.54

Fluorescein azide ditriflate (9). Fluorescein azide (**8**) (1.00 g, 2.18 mmol, 1 eq) was suspended in CH_2Cl_2 (12.5 mL) and cooled to 0 °C under a positive pressure of Ar. Pyridine (2.6 mL, 32.7 mmol, 15 eq) and trifluoromethanesulfonic anhydride (2.75 mL, 16.4 mmol, 7.5 eq) were added, the ice bath removed, and the reaction stirred at room temperature overnight. The crude mixture was subsequently diluted with H_2O (75 mL) and extracted with CH_2Cl_2 (3 x 75 mL). The combined organic extracts were washed with $CuSO_4$ (100 mL of a sat. *aq.* solution) and brine (100 mL), dried with $MgSO_4$, filtered, and concentrated *in vacuo*. Purification by silica gel flash chromatography (CH_2Cl_2:MeOH 197:3 → 195:5) afforded compound **9** (1.28 g, 1.77 mmol, 81 %) as a pale-yellow powder.

R_f = 0.4 (SiO_2; CH_2Cl_2/MeOH, 98:2). **^{1}H NMR** (400 MHz, CD_3OD) δ 8.16 (dd, *J* = 8.1, 1.4 Hz, 1H), 8.13 (d, *J* = 8.1 Hz, 1H), 7.67 (s, 1H), 7.52 (d, *J* = 2.5 Hz, 2H), 7.17 (dd, *J* = 8.9, 2.5 Hz, 2H), 7.10 (d, *J* = 8.8 Hz, 2H), 3.35 (t, *J* = 6.8 Hz, 2H), 3.30 (t, *J* = 5.7 Hz, 2H), 1.76 (app. p, *J* = 6.8 Hz, 2H). **^{13}C NMR** (101 MHz, CD_3OD) δ 169.3, 168.0, 154.0, 152.7, 151.9, 143.0, 131.7, 131.2, 129.2, 126.8, 123.8, 120.4, 120.1 (q, $^1J_{CF}$ = 319.9 Hz), 119.1, 112.1, 81.7, 50.1, 38.6, 29.5. **^{19}F NMR** (377 MHz, CD_3OD) δ -73.1 (s). **IR** (ATR): $\tilde{\nu}$ (cm^{-1}) = 3318 (br), 3076 (w), 2925 (w), 2095 (s), 1772 (s), 1645 (m), 1607 (m), 1539 (m), 1487 (m), 1419 (vs), 1207 (vs) 1136 (vs), 1104 (vs), 985 (vs) 939 (s) 851 (vs), 826 (s), 745 (w), 718 (m). **HRMS** (ESI): *m/z* calcd for $C_{26}H_{17}F_6N_4O_{10}S_2^+$ $[M+H]^+$: 723.0290, found: 723.0289.

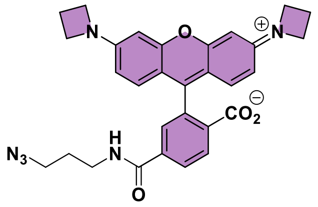

10

Chemical Formula: $C_{30}H_{28}N_6O_4$
Molecular Weight: 536.59

Rhodamine dye RD$_{H2}$ (10). A heavy-walled sealable pressure tube was charged with fluorescein azide ditriflate (**9**) (100 mg, 0.138 mmol, 1 eq), azetidine hydrochloride (130 mg, 1.39 mmol, 10 eq), $Pd_2(dba)_3$ (25 mg, 27.3 µmol, 0.2 eq), XPhos (40 mg, 84.0 µmol, 0.6 eq), and Cs_2CO_3 (902 mg, 2.77 mmol, 20 eq). The reaction vessel was sealed, evacuated, and backfilled with Ar (x3). Dioxane (3 mL) was added, and the reaction was magnetically stirred at 100 °C for 4 h. The crude mixture was subsequently filtered, and the residue washed with dioxane (20 mL). After concentration of the filtrate *in vacuo*, the crude product was precipitated *via* dissolution in the minimum volume of CH_2Cl_2, followed by dropwise addition of the mixture to ^{i}hexane/MeOH (100 mL of a 95 % v/v solution) and storage at 4 ºC overnight. The supernatant was removed by centrifugation, and the precipitate dried *in vacuo*. The crude material was subjected to further rounds of precipitation (x2) according to the same procedure, without storage at 4 ºC. Purification of the solid by basic alumina flash chromatography (CH_2Cl_2/MeOH 1:0 → 93:7 v/v) afforded **RD$_{H2}$** (**10**) (31 mg, 0.058 mmol, 42 %) as a dark, purple solid. A small quantity of the chromatographed material (15 mg) was further subjected to RP-HPLC (30–80 % v/v MeCN/H_2O in 1 h, 5 mL/min, room temperature) to obtain an analytically pure sample for photophysical characterisation.

R_f = 0.4 (SiO_2; CH_2Cl_2/MeOH, 85:15). **^{1}H NMR** (800 MHz, CD_3OD) δ 8.11 (dd, *J* = 8.2, 0.5 Hz, 1H), 8.06 (dd, *J* = 8.1, 1.8 Hz, 1H), 7.67 (dd, *J* = 1.8, 0.5 Hz, 1H), 7.17 (d, *J* = 9.2 Hz, 2H), 6.56 (dd, *J* = 9.2, 2.2 Hz, 2H), 6.49 (d, *J* = 2.2 Hz, 2H), 4.27 (t, *J* = 7.5 Hz, 8H), 3.45 (t, *J* = 6.8 Hz, 2H), 3.40 (t, *J* = 6.7 Hz, 2H), 2.54 (p, *J* = 7.4 Hz, 4H), 1.86 (app. p, *J* = 6.7 Hz, 2H). **^{13}C NMR** (201 MHz, CD_3OD) δ 172.5, 168.7, 161.8, 158.9, 158.0, 144.5, 136.3, 134.1, 132.9, 131.0, 129.6, 129.3, 115.0, 113.1, 95.0, 52.7, 50.2, 38.6, 29.7, 16.8. **IR** (ATR): $\tilde{v}$ (cm^{-1}) = 3260 (br), 2928 (s), 2857 (s), 2094 (s), 1751 (w), 1592 (vs), 1550 (s), 1532 (s), 1467 (s), 1408 (s), 1377 (vs), 1343 (s), 1296 (s), 1255 (vs), 1221 (s), 1181 (s), 1137 (s), 1113 (s), 1068 (s), 1029 (vs), 971 (w), 928 (w), 818 (s), 754 (w), 697 (w). **HRMS** (ESI): *m/z* calcd for $C_{30}H_{29}N_6O_4^+$ $[M+H]^+$: 537.2250, found: 537.2248.

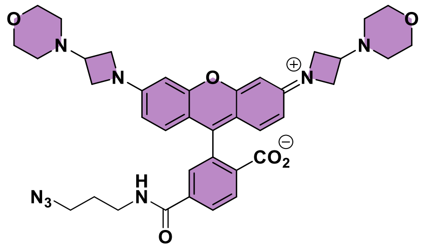

11
Chemical Formula: $C_{38}H_{42}N_8O_6$
Molecular Weight: 706.80

Rhodamine dye RD$_m$ (11). A heavy-walled sealable pressure tube was charged with fluorescein ditriflate (**9**) (100 mg, 0.138 mmol, 1 eq), 4-(azetidin-3-yl)morpholine hydrochloride (247 mg, 1.38 mmol, 10 eq), $Pd_2(dba)_3$ (25 mg, 27.3 µmol, 0.2 eq), XPhos (40 mg, 84.0 µmol, 0.6 eq) and Cs_2CO_3 (902 mg, 2.77 mmol, 20 eq). The reaction vessel was sealed, evacuated, and backfilled with Ar (x3). Dioxane (3 mL) was added, and the reaction was then stirred at 100 °C for 4 h. The crude mixture was subsequently filtered, and the residue washed with dioxane (20 mL). After concentration of the filtrate *in vacuo*, the crude product was precipitated *via* dissolution in the minimum volume of CH_2Cl_2, followed by dropwise addition of the mixture to ^{i}hexane/MeOH (100 mL of a 95 % v/v solution) and storage at 4 ºC overnight. The supernatant was removed by centrifugation, and the precipitate dried *in vacuo*. The crude material was subjected to further rounds of precipitation (x2) according to the same procedure, without storage at 4 ºC. Purification of the solid by basic alumina flash chromatography (CH_2Cl_2:MeOH 1:0 → 9:1 v/v) afforded **RD$_m$** (**11**) (59 mg, 0.083 mmol, 60 %) as a purple solid. A small quantity of the chromatographed material (15 mg) was further subjected to RP-HPLC (0-80 % v/v MeCN/H_2O in 30 min, 5 mL/min, room temperature) to obtain an analytically pure sample for photophysical characterisation.

R_f = 0.4 (SiO_2; CH_2Cl_2/MeOH, 95:5). **^{1}H NMR** (800 MHz, CD_3OD) δ 8.13 (d, *J* = 8.2 Hz, 1H), 8.07 (dd, *J* = 8.2, 1.8 Hz, 1H), 7.67 (d, *J* = 1.8 Hz, 1H), 7.19 (d, *J* = 9.2 Hz, 2H), 6.62 (dd, *J* = 9.2, 2.2 Hz, 2H), 6.57 (d, *J* = 2.2 Hz, 2H), 4.34 – 4.24 (m, 4H), 4.15 – 4.08 (m, 4H), 3.46 (t, *J* = 6.8 Hz, 2H), 3.40 (t, *J* = 6.6 Hz, 2H), 2.50 (br, 8H), 1.87 (app. p, *J* = 6.7 Hz, 2H). **^{13}C NMR** (201 MHz, CD_3OD) δ 172.3, 168.7, 159.5, 158.7, 157.8, 143.9, 136.6, 134.5, 133.0, 130.9, 129.4, 129.3, 115.1, 113.3, 95.6, 67.5, 56.1, 55.7, 51.1, 50.2, 38.6, 29.7. **IR** (ATR): $\tilde{v}$ (cm^{-1}) = 3304 (br), 2923 (s) , 2851 (s), 2095 (s), 1754 (s), 1632 (w), 1594 (vs), 1552 (w), 1513 (w), 1481 (w), 1450 (w), 1428 (w), 1407 (w), 1377 (s), 1341 (w), 1320 (w), 1293 (s), 1270 (w), 1245 (w), 1215 (w), 1185 (s), 1139 (w), 1111 (vs), 1067 (w), 965 (w), 926 (w), 888 (w), 864 (w), 820 (s), 750 (w), 716 (w), 691 (w). **HRMS** (ESI): *m*/*z* calcd for $C_{38}H_{43}N_8O_6^+$ $[M+H]^+$: 707.3306, found: 707.3301.

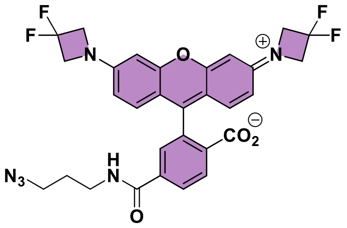

12
Chemical Formula: $C_{30}H_{24}F_4N_6O_4$
Molecular Weight: 608.55

Rhodamine dye RD$_{F2}$ (12). A heavy-walled sealable pressure tube was charged with fluorescein ditriflate (**9**) (100 mg, 0.138 mmol, 1 eq), 4,4-difluoroazetidine hydrochloride (179 mg, 1.38 mmol, 10 eq), $Pd_2(dba)_3$ (25 mg, 0.027 mmol, 0.2 eq), XPhos (40 mg, 0.084 mmol, 0.6 eq) and Cs_2CO_3 (902 mg, 2.77 mmol, 20 eq). The reaction vessel was sealed, evacuated, and backfilled with Ar (x3). Dioxane (3 mL) was added, and the reaction was then stirred at 100 °C for 4 h. The crude mixture was subsequently filtered, and the residue washed with dioxane (20 mL). After concentration of the filtrate *in vacuo*, the crude product was precipitated *via* dissolution in the minimum volume of CH_2Cl_2, followed by dropwise addition of the mixture to ^{i}hexane/MeOH (100 mL of a 95 % v/v solution) and storage at 4 ºC overnight. The supernatant was removed by centrifugation, and the precipitate dried *in vacuo*. The crude material was subjected to further rounds of precipitation (x2) according to the same procedure, without storage at 4 ºC, to afford **RD$_{F2}$** (**12**) (62 mg, 0.10 mmol, 74 %) as a purple solid. A small quantity of the chromatographed material (15 mg) was further subjected to RP-HPLC (30–80 % v/v MeCN/H_2O in 1 h, 5 mL/min, room temperature) to obtain an analytically pure sample for photophysical characterisation.
R_f = 0.5 (SiO_2; CH_2Cl_2/MeOH, 8:2). **^{1}H NMR** (800 MHz, CD_3OD) δ 8.11 (dd, J = 8.1, 1.4 Hz, 1H), 8.08 (d, J = 8.1 Hz, 1H), 7.59 (d, J = 1.7 Hz, 1H), 6.71 (d, J = 8.6 Hz, 2H), 6.46 (d, J = 2.4 Hz, 2H), 6.36 (dd, J = 8.7, 2.4 Hz, 2H), 4.31 (t, J = 11.5 Hz, 8H), 3.38 (t, J = 6.8 Hz, 2H), 3.34 (t, J = 6.6 Hz, 2H), 1.80 (app. p, J = 6.8 Hz, 2H). **^{13}C NMR** (201 MHz, CD_3OD) δ 170.8, 168.3, 154.5, 153.9, 152.2, 141.5, 132.2, 130.5, 130.2, 126.8, 124.7, 117.3 (t, $^1J_{CF}$ = 272.7 Hz), 110.9, 110.7, 100.0, 96.8, 64.2 (t, $^2J_{CF}$ = 26.6 Hz), 50.2, 38.6, 29.6. **^{19}F NMR** (377 MHz, CD_3OD) δ -100.1 (p, $^3J_{FH}$ = 11.8 Hz). **IR** (ATR): ṽ (cm^{-1}) = 3321 (br), 2928 (w), 2862 (w), 2095 (s), 1754 (s), 1632 (m), 1610 (s), 1553 (m), 1511 (s), 1462 (m), 1429 (s), 1370 (s), 1352 (m), 1318 (s), 1295 (m), 1270 (m), 1222 (vs), 1124 (s), 1086 (s), 960 (w), 906 (s), 857 (w), 821 (w), 810 (w), 750 (w), 725 (s), 664 (s), 664 (s). **HRMS** (ESI): m/z calcd for $C_{30}H_{25}F_4N_6O_4^+$ $[M+H]^+$: 609.1873, found: 609.1877.

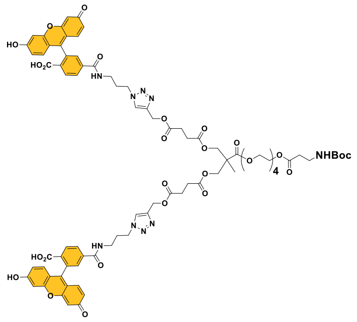

13
Chemical Formula: $C_{83}H_{87}N_9O_{29}$
Molecular Weight: 1674.64

Dendritic dye FD2 (13). A Schlenk tube was charged with bis-MPA Acetylene Dendron, NH-Boc Core, Generation 1 (50 mg, 0.066 mmol, 1 eq), fluorescein azide (**8**) (121 mg, 0.264 mmol, 4 eq), CuBr (9 mg, 0.06 mmol, 0.9 eq) and *N*,*N*,*N′*,*N″*,*N″*-pentamethyldiethylenetriamine (PMDTA) (27.8 µL, 0.132 mmol, 2 eq) under a positive pressure of Ar. Anhydrous DMSO (0.5 mL) was added, and the reaction mixture was stirred for 2 h at 50 ºC. After cooling to room temperature, the crude reaction mixture was subjected to dialysis (1 KDa MWCO) against DMSO for 72 h and then against DI H_2O for 24 h according to the procedure specified in the General Experimental Methods section. Concentration of the dialysed solution *in vacuo* afforded **FD2** (**7**) (67 mg, 0.040 mmol, 61 %) as an orange solid.

$\mathbf{R}_t$ = 5.94 min (Analytical RP-HPLC; 1.5 mL/min, 40 °C, buffer B 0-30 % v/v 0 → 4 min, then 30-85 % v/v 4 → 10 min). **^{1}H NMR** (800 MHz, DMSO-*d*$_6$) δ 10.36 (br, 4H), 8.75 (s, 2H), 8.14 (br, 6H), 7.69 (br, 2H), 6.82 (t, *J* = 5.6 Hz, 1H), 6.77 – 6.17 (m, 12H), 5.08 (s, 4H), 4.37 (s, 4H), 4.21 – 4.06 (m, 8H), 3.60 – 3.55 (m, 4H), 3.52 – 3.47 (m, 8H), 3.23 (br, 4H), 3.14 (app. q, *J* = 6.5 Hz, 2H), 2.56 (s, 8H), 2.42 (t, *J* = 7.1 Hz, 2H), 2.03 (br, 4H), 1.35 (s, 9H), 1.13 (s, 3H). **^{13}C NMR** (201 MHz, DMSO-*d*$_6$) δ 172.2, 171.7, 171.4, 171.2, 164.8, 155.4, 141.6, 129.2, 124.8, 111.6, 102.4, 77.7, 69.7, 68.2, 68.1, 65.1, 64.0, 63.2, 57.4, 47.4, 45.9, 36.7, 36.1, 34.1, 29.6, 28.5, 28.4, 28.2, 17.1. **IR** (ATR): ṽ (cm^{-1}) = 3279 (br), 2962 (w), 1737 (vs), 1640 (m), 1591 (s), 1506 (m), 1461 (s), 1385 (m), 1316 (m), 1250 (vs), 1209 (s), 1174 (vs), 1108 (vs), 1027 (m), 851 (s), 808 (s), 759 (w), 662 (w). **MS (MALDI-ToF)**: *m*/*z* calcd for $C_{83}H_{87}N_9NaO_{29}^+$ $[M+Na]^+$: 1696.5502 (monoisotopic mass), 1697.6322 (molecular weight), found: 1697.9.

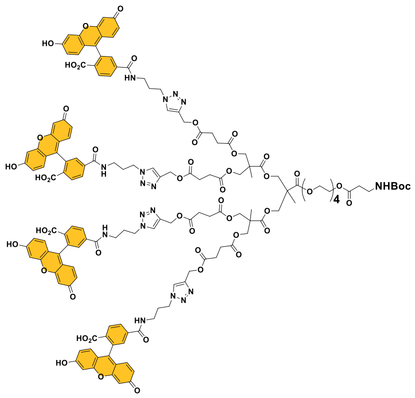

14
Chemical Formula: $C_{155}H_{151}N_{17}O_{53}$
Molecular Weight: 3099.98

Dendritic dye FD4 (14). A Schlenk tube was charged with bis-MPA Acetylene Dendron, NH-Boc Core, Generation 2 (50 mg, 0.040 mmol, 1 eq), fluorescein azide (**8**) (145 mg, 0.316 mmol, 8 eq), CuBr (11 mg, 0.077 mmol, 1.9 eq) and *N*,*N*,*N'*,*N''*,*N''*-pentamethyldiethylenetriamine (PMDTA) (33.0 µL, 0.158 mmol, 4 eq) under a positive pressure of Ar. Anhydrous DMSO (0.5 mL) was added, and the reaction mixture was stirred for 2 h at 50 ºC. After cooling to room temperature, the crude reaction mixture was subjected to dialysis (1 KDa MWCO) against DMSO for 72 h and then against DI H_2O for 24 h according to the procedure specified in the General Experimental Methods section. Concentration of the dialysed solution *in vacuo* afforded **FD4** (**14**) (82 mg, 0.026 mmol, 67 %) as an orange solid.
R_t = 5.88 min (Analytical RP-HPLC; buffer B 0-30 % v/v 0 → 4 min, then 30-85 % v/v 4 → 10 min). **^{1}H NMR** (800 MHz, DMSO-d_6) δ 10.50 (s, 8H), 8.77 (s, 4H), 8.60 – 7.88 (m, 12H), 7.85 – 7.40 (m, 4H), 6.81 (t, J = 5.6 Hz, 1H), 6.78 – 6.27 (m, 24H), 5.07 (s, 8H), 4.36 (t, J = 6.9 Hz, 8H), 4.25 – 4.04 (m, 16H), 3.58 (t, J = 4.9 Hz, 2H), 3.56 (t, J = 4.8 Hz, 2H), 3.53 – 3.44 (m, 8H), 3.27 – 3.18 (m, 8H), 3.13 (q, J = 6.7 Hz, 2H), 2.54 (s, 16H), 2.41 (t, J = 7.0 Hz, 2H), 2.02 (app. p, J = 7.4 Hz, 8H), 1.34 (s, 9H), 1.18 (s, 3H), 1.11 (s, 6H). **^{13}C NMR** (201 MHz, DMSO-d_6) δ 172.0, 171.7, 171.6, 171.4, 171.2, 164.9, 155.4, 152.5, 141.5, 139.6, 129.3 (br, x2), 124.8, 123.1, 114.1, 109.5, 102.4, 77.7, 69.7, 68.2, 68.0, 65.5, 65.1, 64.1, 63.2, 57.4, 47.4, 46.1, 46.0, 36.7, 36.1, 34.1, 29.6, 28.4, 28.3, 28.2, 17.0, 16.9. **IR** (ATR): $\tilde{\nu}$ (cm^{-1}) = 3289 (br), 2927 (w), 1737 (vs), 1639 (m), 1591 (vs), 1506 (s), 1462 (vs), 1383 (s), 1318 (s), 1248 (vs), 1208 (vs), 1157 (vs), 1108 (vs), 1053 (w), 1028 (w), 995 (w), 968 (w), 921 (w), 850 (s), 808 (w), 758 (m), 717 (w), 663 (w). **MS (MALDI-ToF)**: *m*/*z* calcd for $C_{155}H_{151}N_{17}NaO_{53}^+$ [M+Na]$^+$: 3120.9535 (monoisotopic mass), 3122.9682 (molecular weight), found: 3121.9.

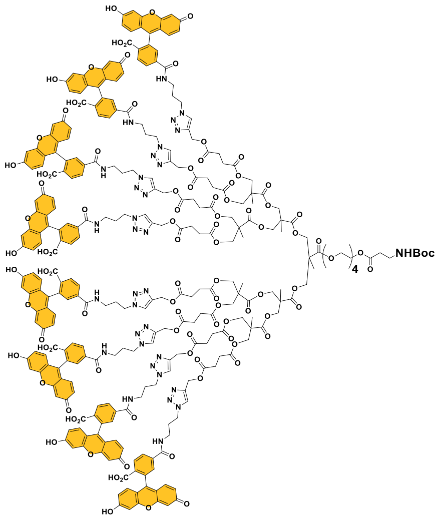

15
Chemical Formula: $C_{299}H_{279}N_{33}O_{101}$
Molecular Weight: 5950.65

Dendritic dye FD8 (15). A Schlenk tube was charged with bis-MPA Acetylene Dendron, NH-Boc Core, Generation 3 (47 mg, 0.021 mmol, 1 eq), fluorescein azide (**8**) (154 mg, 0.336 mmol, 16 eq), CuBr (12 mg, 0.084 mmol, 4 eq) and *N*,*N*,*N'*,*N''*,*N''*-pentamethyldiethylenetriamine (PMDTA) (34.2 µL, 0.164 mmol, 8 eq) under a positive pressure of Ar. Anhydrous DMSO (0.5 mL) was added, and the reaction mixture was stirred for 2 h at 50 ºC. After cooling to room temperature, the crude reaction mixture was subjected to dialysis (3.5 KDa MWCO) against DMSO for 24 h and then against DI H_2O for 24 h according to the procedure specified in the General Experimental Methods section. Concentration of the dialysed solution *in vacuo* afforded **FD8** (**15**) (50 mg, 8.4 µmol, 40 %) as a yellow solid.

R_t = 5.64 min (Analytical RP-HPLC; 1.5 mL/min, 40 °C, buffer B 0-30 % v/v 0 → 4 min, then 30-85 % v/v 4 → 10 min). **^{1}H NMR** (800 MHz, DMSO-d_6) δ 10.17 (s, 16H), 8.75 (t, *J* = 5.6 Hz, 8H), 8.15 (dd, *J* = 8.1, 1.4 Hz, 8H), 8.10 (s, 8H), 8.06 (d, *J* = 8.1 Hz, 8H), 7.67 (s, 8H), 6.77 (t, *J* = 5.7 Hz, 1H), 6.68 (d, *J* = 2.4 Hz, 16H), 6.58 (d, *J* = 8.6 Hz, 16H), 6.54 (dd, *J* = 8.7, 2.4 Hz, 16H), 5.05 (s, 16H), 4.34 (t, *J* = 7.1 Hz, 16H), 4.26 – 4.03 (m, 32H), 3.60 – 3.56 (m, 2H), 3.55 – 3.52 (m, 2H), 3.51 – 3.42 (m, 8H), 3.20 (app. q, *J* = 6.5 Hz, 16H), 3.12 (q, *J* = 6.7 Hz, 2H), 2.52 (s, 32H), 2.39 (t, *J* = 7.0 Hz, 2H), 2.00 (app. p, *J* = 7.0 Hz, 16H), 1.31 (s, 9H), 1.20 (s, 3H), 1.17 (s, 6H), 1.10 (s, 12H). **^{13}C NMR** (201 MHz, DMSO-d_6) δ 171.9, 171.7, 171.6, 171.4, 171.2, 168.0, 164.7, 159.8, 155.4, 152.4, 151.9, 141.5, 140.5, 129.4, 129.3, 128.5, 124.9, 124.8, 122.3, 112.9, 109.2, 102.3, 77.7, 69.7, 69.6, 68.2, 68.0, 65.1, 65.0, 64.1, 63.2, 57.3, 47.4, 46.2, 46.1, 46.0, 36.8, 36.0, 34.1, 29.6, 28.3, 28.3, 28.1, 17.0, 16.9, 16.8. **IR** (ATR): $\tilde{\nu}$ (cm^{-1}) = 3260 (br), 2922 (s), 2852 (m), 1734 (vs), 1635 (m), 1610 (s), 1543 (m), 1506 (m), 1450 (s), 1368 (m), 1315 (m), 1239 (s), 1175 (vs), 1152 (vs), 1110 (vs), 994 (s), 949 (w), 847 (s), 822 (m), 752 (m), 688 (m). **MS (MALDI-ToF)**: *m*/*z* calcd for $C_{299}H_{279}N_{33}NaO_{101}^+$ [M+Na]$^+$: 5969.7602 (monoisotopic mass), 5973.6402 (molecular weight), found: 5970.0. *m*/*z* calcd for $C_{299}H_{278}N_{33}Na_2O_{101}^+$ [M-H+2Na]$^+$: 5991.7427 (monoisotopic mass), 5995.6225 (molecular weight), found: 5991.5.

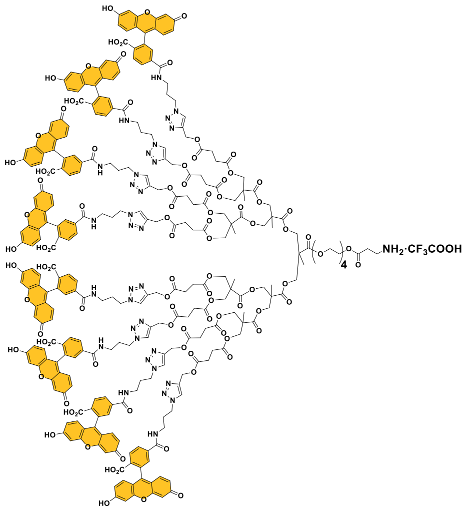

15b
Chemical Formula: $C_{296}H_{272}F_3N_{33}O_{101}$
Molecular Weight: 5964.56

Dendritic dye 15b. Dendritic dye **FD8** (**15**) (15 mg, 2.5 μmol) was suspended in *neat* trifluoroacetic acid (18 μL) and the resulting mixture was maintained at room temperature for 2 h with vigorous stirring. After this time, the reaction mixture was subjected to successive strip cycles (x3) by addition and evaporation of methanol *in vacuo* to afford **15b** (15 mg, 2.5 μmol, quant.) as a yellow solid.

R_t = 5.23 min (Analytical RP-HPLC; 1.5 mL/min, 40 °C, buffer B 0-30 % v/v 0 → 4 min, then 30-85 % v/v 4 → 10 min). **^{1}H NMR** (800 MHz, DMSO-d_6) δ 10.16 (s, 16H), 8.75 (t, *J* = 5.5 Hz, 8H), 8.15 (dd, *J* = 8.1, 1.4 Hz, 8H), 8.11 (s, 8H), 8.06 (d, *J* = 8.2 Hz, 8H), 7.71 (br, 3H), 7.66 (s, 8H), 6.69 (d, *J* = 2.4 Hz, 16H), 6.58 (d, *J* = 8.7 Hz, 16H), 6.54 (dd, *J* = 8.7, 2.4 Hz, 16H), 5.05 (s, 16H), 4.34 (t, *J* = 7.1 Hz, 16H), 4.25 – 4.02 (m, 32H), 3.60 – 3.54 (m, 4H), 3.52 – 3.42 (m, 8H), 3.20 (app. q, *J* = 6.4 Hz, 16H), 3.02 (app. h, *J* = 6.3 Hz, 2H), 2.64 (t, *J* = 6.9 Hz, 2H), 2.54 – 2.51 (m, 32H), 2.00 (app. p, *J* = 7.0 Hz, 16H), 1.23 (s, 3H), 1.17 (d, *J* = 5.2 Hz, 6H), 1.10 (s, 12H). **^{13}C NMR** (201 MHz, DMSO-d_6) δ 171.7, 171.6, 171.4, 170.4, 168.0, 164.7, 159.6, 157.9, 152.7, 151.8, 141.5, 140.6, 129.4, 129.3, 128.2, 124.8, 124.8, 122.3, 112.7, 109.1, 102.2, 83.3, 69.6, 68.1, 68.0, 65.1, 65.0, 63.8, 57.3, 47.4, 46.2, 46.1, 46.0, 36.8, 34.7, 31.3, 29.6, 28.3, 28.3, 17.0, 16.9, 16.8. **IR** (ATR): $\tilde{v}$ (cm^{-1}) = 3430 (br), 3186 (br), 2924 (s), 2850 (m), 2158 (w), 1731 (s), 1652 (s), 1606 (s), 1505 (w), 1453 (m), 1378 (w), 1314 (w), 1240 (m), 1177 (vs), 1128 (vs), 992 (m), 841 (m), 803 (m), 759 (w), 749 (w), 720 (m), 685 (w). **MS (MALDI-ToF)**: *m/z* calcd for $C_{294}H_{271}N_{33}NaO_{99}^+$ [M-H+Na]$^+$: 5869.7083 (monoisotopic mass), 5873.5238 (molecular weight), found: 5870.1. *m/z* calcd for $C_{294}H_{270}N_{33}Na_2O_{99}^+$ [M-2H+2Na]$^+$: 5891.6897 (monoisotopic mass), 5895.5050 (molecular weight), found: 5891.4.

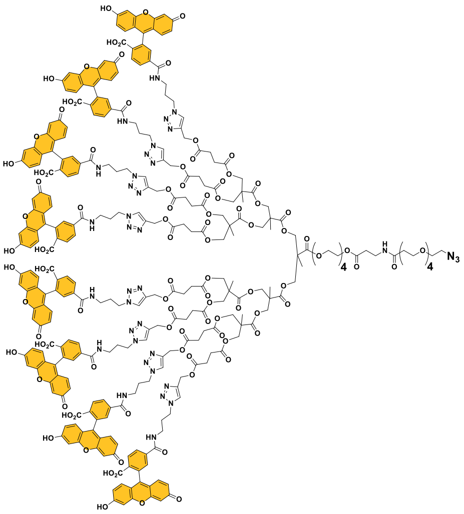

16
Chemical Formula: $C_{305}H_{290}N_{36}O_{104}$
Molecular Weight: 6123.82

Dendritic dye FD8-N$_3$ (16). Dendritic dye **15b** (10 mg, 1.7 µmol, 1 eq) was dissolved in DMF (100 µL). Triethylamine (1.2 µL, 8.4 µmmol, 5 eq) and azido-PEG4-NHS ester (1.3 mg, 3.4 µmol, 2 eq) were added and the resulting mixture was maintained at room temperature for 1 h with vigorous stirring. After this time, the crude reaction mixture was concentrated *in vacuo*, dissolved in the minimum amount of DMSO and subjected to dialysis (3.5 KDa MWCO) against DI H_2O for 48 h according to the procedure specified in the General Experimental Methods section. Concentration of the dialysed solution *in vacuo* afforded **FD8-N$_3$** (**16**) (7 mg, 1 µmol, 67 %) as an orange solid.

R$_t$ = 5.55 min (Analytical RP-HPLC; 1.5 mL/min, 40 °C, buffer B 0-30 % v/v 0 → 4 min, then 30-85 % v/v 4 → 10 min). **^{1}H NMR** (800 MHz, DMSO-d_6) δ 10.18 (s, 16H), 8.74 (t, *J* = 5.6 Hz, 8H), 8.14 (dd, *J* = 8.0, 1.4 Hz, 8H), 8.09 (s, 8H), 8.05 (d, *J* = 8.1 Hz, 8H), 7.88 (t, *J* = 5.7 Hz, 1H), 7.66 (s, 8H), 6.68 (d, *J* = 2.4 Hz, 16H), 6.58 (d, *J* = 8.6 Hz, 16H), 6.54 (dd, *J* = 8.6, 2.5 Hz, 16H), 5.04 (s, 16H), 4.33 (t, *J* = 6.8 Hz, 16H), 4.25 – 3.98 (m, 32H), 3.64 – 3.31 (m, 30H), 3.23 (app. q, *J* = 6.7 Hz, 2H), 3.20 (q, *J* = 6.5 Hz, 16H), 2.54 – 2.46 (m, 34H), 2.25 (t, *J* = 6.5 Hz, 2H), 2.00 (app. p, *J* = 7.0 Hz, 16H), 1.19 (s, 3H), 1.16 (s, 6H), 1.09 (s, 12H). **^{13}C NMR** (201 MHz, DMSO-d_6) δ 171.8, 171.7, 171.5, 171.4, 170.3, 168.1, 164.8, 159.7, 152.7, 151.9, 141.6, 140.7, 129.7, 129.5, 129.3, 128.3, 124.9, 124.8, 122.3, 112.8, 109.2, 102.3, 83.4, 69.8 (x2), 69.8, 69.7 (x2), 69.6, 69.3, 68.2, 68.0, 66.8, 65.2, 65.0, 63.3, 57.4, 50.0, 47.4, 46.2 (x2), 46.1, 36.8, 29.6, 28.4 (x2), 17.1, 17.0, 16.9. **IR** (ATR): ṽ (cm^{-1}) = 3130 (br), 2923 (m), 2853 (w), 2357 (w), 2251 (w), 2110 (w), 1734 (vs), 1634 (m), 1610 (s), 1545 (m), 1507 (m) 1451 (s), 1368 (w), 1242 (s), 1177 (vs), 1150 (vs), 1110 (vs), 1084 (m), 1045 (m), 1023 (s), 993 (vs), 848 (m), 818 (s), 757 (m), 688 (w), 674 (w), 688 (w). **MS (MALDI-ToF)**: *m*/*z* calcd for $C_{305}H_{290}N_{36}NaO_{104}^+$ [M+Na]$^+$: 6142.8403 (monoisotopic mass), 6146.8122 (molecular weight), found: 6145.6. *m*/*z* calcd for $C_{305}H_{289}N_{36}Na_2O_{104}^+$ [M-H+2Na]$^+$: 6164.8222 (monoisotopic mass), 6148.7940 (molecular weight), found: 6167.1.

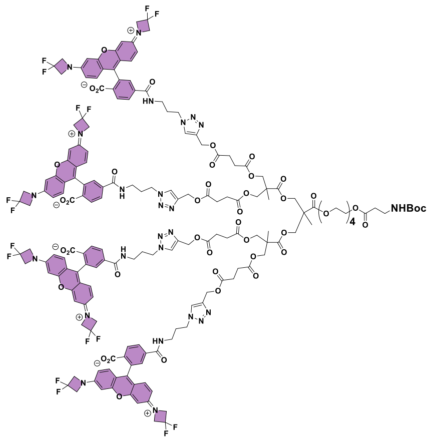

17
Chemical Formula: $C_{179}H_{175}F_{16}N_{25}O_{45}$
Molecular Weight: 3700.47

Dendritic dye RD$_{F2}$D4 (17). A vial was charged with bis-MPA Acetylene Dendron, Generation 2 (5 mg, 4 µmol, 1 eq), dye **RD$_{F2}$** (**12**) (14 mg, 23 µmol, 5.8 eq), CuBr (1.1 mg, 7.9 µmol, 2 eq) and *N*,*N*,*N′*,*N″*,*N″*-pentamethyldiethylenetriamine (PMDTA) (3.3 µL, 15.8 µmol, 4 eq) under Ar atmosphere. After dissolution with 300 µL of anhydrous DMF, the reaction mixture was stirred for 2 h at 50 ºC. The crude was then purified by size exclusion chromatography (Sephadex LH-20, DMF) to yield **RD$_{F2}$D4** (**17**) (12 mg, 3.2 µmol, 85 %) as a pink solid.

R_f = 0.6 (SiO_2; EtOAc/MeOH, 8:2). **^{1}H NMR** (600 MHz, DMSO-*d$_6$*) δ 8.76 (s, 4H), 8.16 (d, *J* = 6.6 Hz, 4H), 8.11 (s, 4H), 8.08 (d, *J* = 8.1 Hz, 4H), 7.62 (s, 4H), 6.80 (s, 1H), 6.62 (dd, *J* = 8.6, 2.3 Hz, 8H), 6.46 (s, 8H), 6.31 (d, *J* = 8.6, 8H), 5.07 (s, 8H), 4.40 – 4.27 (m, 40H), 4.22 – 4.06 (m, 16H), 3.64 – 3.53 (m, 4H), 3.49 (t, *J* = 9.0 Hz, 8H), 3.23 – 3.20 (m, 8H), 3.16 – 3.11 (m, 2H), 2.54 (s, 16H), 2.42 (t, *J* = 7.0 Hz, 2H), 2.01 (t, *J* = 7.0 Hz, 8H), 1.34 (s, 9H), 1.18 (s, 3H), 1.12 (s, 6H). **^{13}C NMR** (151 MHz, DMSO-*d$_6$*) δ 172.0, 171.7, 171.6, 171.4, 171.2, 168.1, 164.7, 155.4, 152.7, 151.6, 141.5, 140.5, 128.9, 128.3, 124.9, 124.8, 122.2, 116.5 (t, $^1J_{CF}$ = 273.1 Hz), 109.6, 108.3, 99.2, 83.5, 77.7, 69.7, 68.2, 68.0, 65.1, 64.1, 63.2, 62.9 (t, $^2J_{CF}$ = 25.3 Hz), 57.3, 47.3, 46.1, 36.7, 36.0, 34.1, 29.6, 28.3, 28.2, 17.0, 16.9. **^{19}F NMR** (377 MHz, DMSO-*d$_6$*) δ -101.6 (p, $^3J_{FH}$ = 12.9 Hz). **IR** (ATR): ṽ (cm^{-1}) = 3353 (br), 2954 (s), 2921 (vs), 2851 (vs), 2364 (w), 1738 (s), 1634 (w), 1613 (s), 1514 (m), 1463 (s), 1429 (w), 1377 (s), 1320 (w), 1270 (m), 1230 (s), 1087 (w), 965 (w), 910 (m), 830 (w), 727 (m). **MS (MALDI-ToF)**: *m*/*z* calcd for $C_{179}H_{175}N_{25}O_{45}^+$ $[M+H]^+$: 3699.1991 (monoisotopic mass), 3701.4809 (molecular weight), found: 3702.7; *m*/*z* calcd for $C_{179}H_{175}N_{25}NaO_{45}^+$ $[M+Na]^+$: 3721.1811 (monoisotopic mass), 3723.4627 (molecular weight), found: 3724.7.

3. Photophysical Properties of the Synthesised Fluorophores

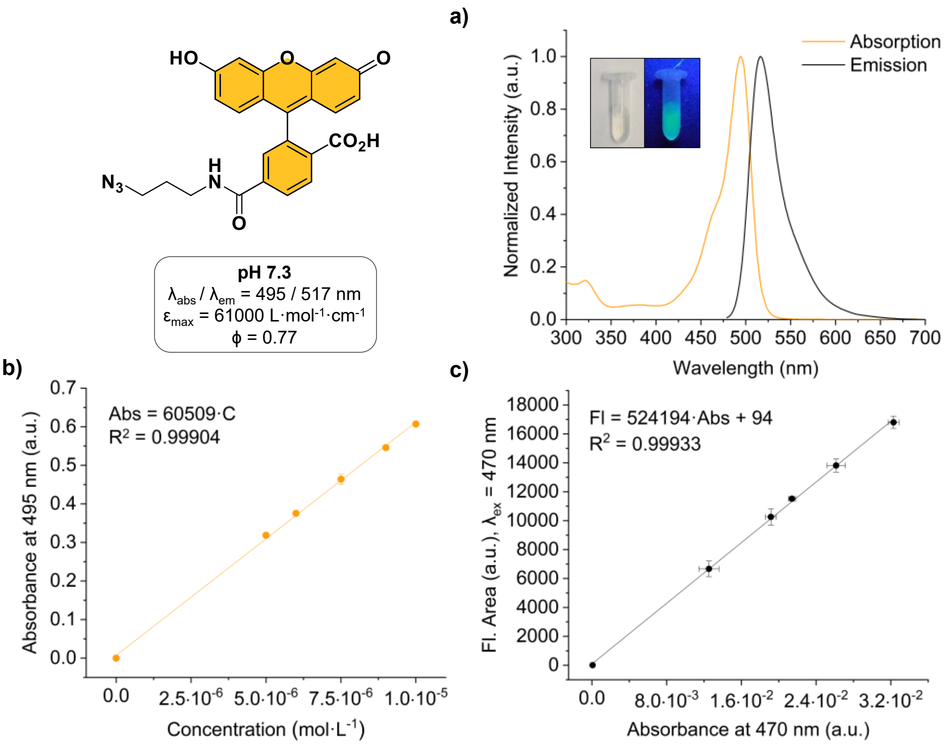

Figure S1. Photophysical properties of **fluorescein azide** (**8**) in 10 mM HEPES, pH 7.3 buffer at room temperature. a) Absorption and emission spectra. Solutions in the photographs correspond to **fluorescein azide** at 2 μM (absorbance; left) and 0.2 μM (emission; right). b) Linear regression analysis obeying the Beer-Lamber law. All absorbance values are averages (n = 3). c) Fluorescence area *vs.* absorbance plot for the quantum yield comparative method. All absorbance and fluorescence area values are averages (n = 3).

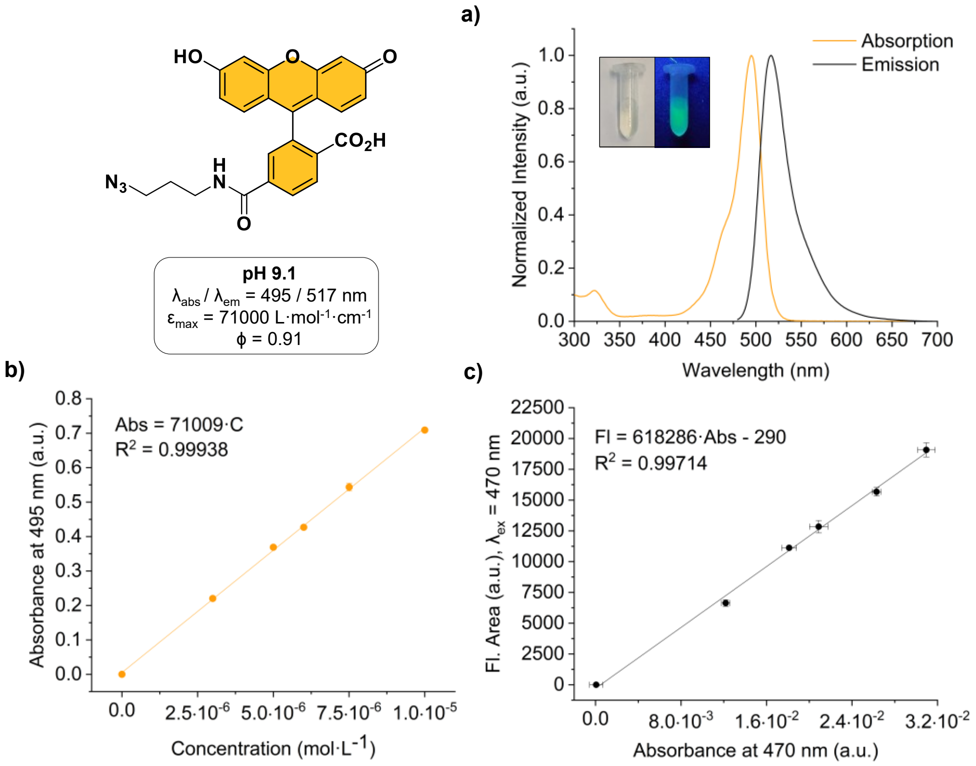

Figure S2. Photophysical properties of **fluorescein azide** (**8**) in 10 mM sodium borate, pH 9.1 buffer at room temperature. a) Absorption and emission spectra. Solutions in the photographs correspond to **fluorescein azide** at 2 μM (absorbance; left) and 0.2 μM (emission; right). b) Linear regression analysis obeying the Beer-Lamber law. All absorbance values are averages (n = 3). c) Fluorescence area *vs.* absorbance plot for the quantum yield comparative method. All absorbance and fluorescence area values are averages (n = 3).

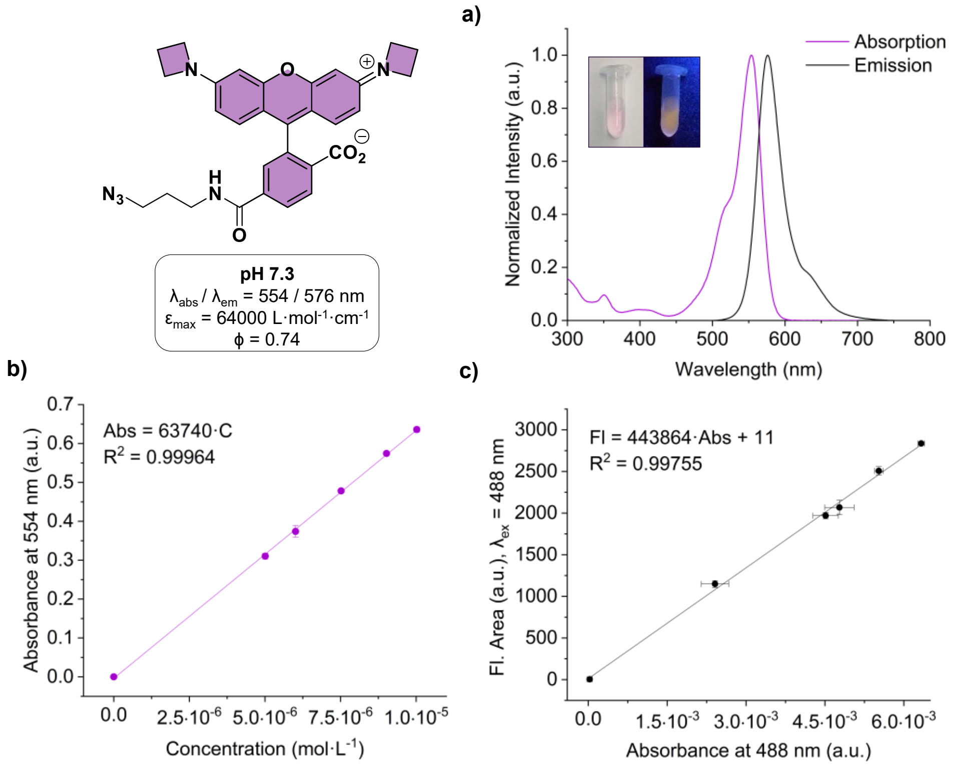

Figure S3. Photophysical properties of **RD$_{H2}$** (**10**) in 10 mM HEPES, pH 7.3 buffer at room temperature. a) Absorption and emission spectra. Solutions in the photographs correspond to **RD$_{H2}$** at 2 µM (absorbance; left) and 0.2 µM (emission; right). b) Linear regression analysis obeying the Beer-Lamber law. All absorbance values are averages (n = 3). c) Fluorescence area *vs.* absorbance plot for quantum yield comparative method. All absorbance and fluorescence area values are averages (n = 3).

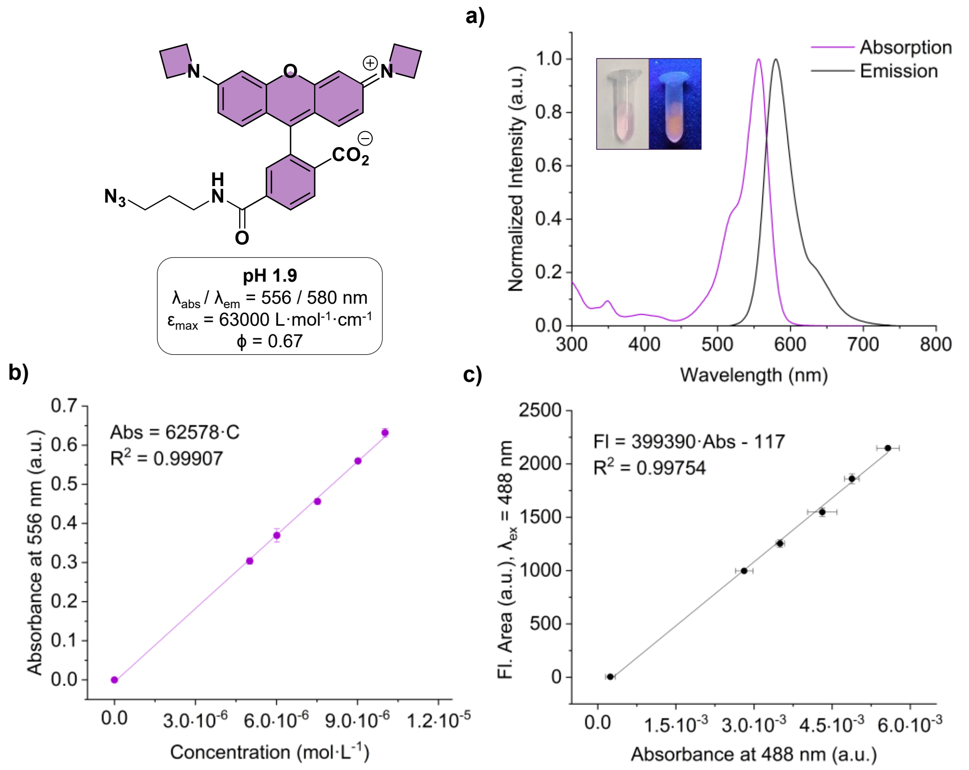

Figure S4. Photophysical properties of **RD$_{H2}$** (**10**) in aqueous TFA (0.1 % v/v) at room temperatures. a) Absorption and emission spectra. Solutions in the photographs correspond to **RD$_{H2}$** at 2 µM (absorbance; left) and 0.2 µM (emission; right). b) Linear regression analysis obeying the Beer-Lamber law. All absorbance values are averages (n = 3). c) Fluorescence area *vs.* absorbance plot for the quantum yield comparative method. All absorbance and fluorescence area values are averages (n = 3).

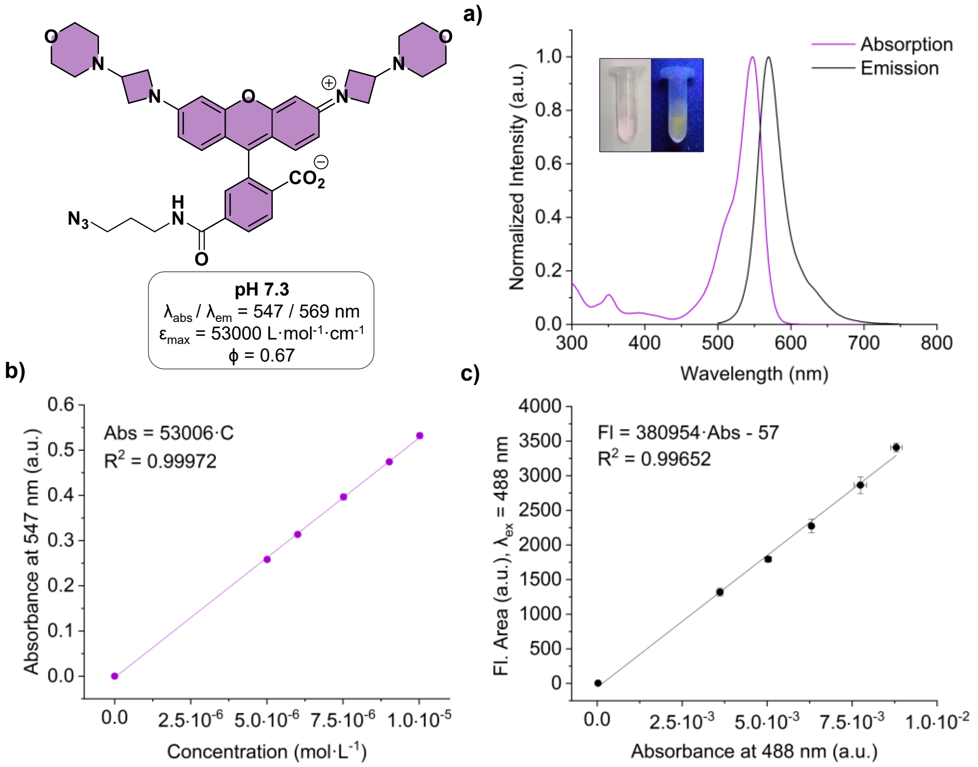

Figure S5. Photophysical properties of **RD$_m$** (**11**) in 10 mM HEPES, pH 7.3 buffer at room temperature. a) Absorption and emission spectra. Solutions in the photographs correspond to **RD$_m$** at 2 µM (absorbance; left) and 0.2 µM (emission; right). b) Linear regression analysis obeying the Beer-Lamber law. All absorbance values are averages (n = 3). c) Fluorescence area *vs.* absorbance plot for the quantum yield comparative method. All absorbance and fluorescence area values are averages (n = 3).

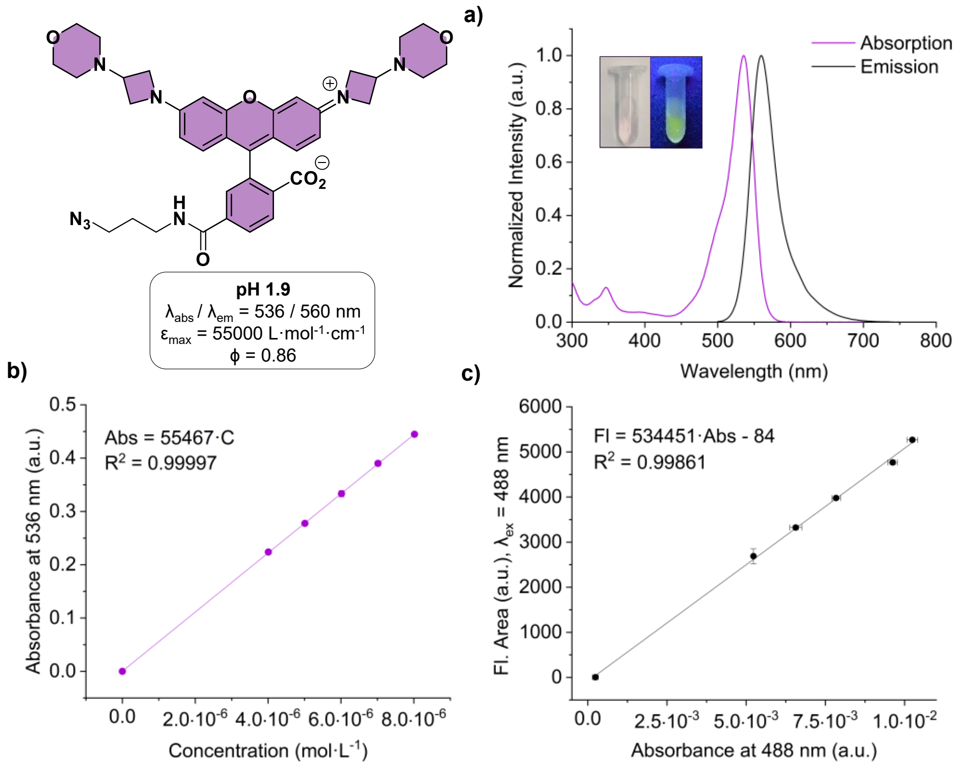

Figure S6. Photophysical properties of **RD$_m$** (**11**) in aqueous TFA (0.1 % v/v) at room temperature. a) Absorption and emission spectra. Solutions in the photographs correspond to **RD$_m$** at 2 µM (absorbance; left) and 0.2 µM (emission; right). b) Linear regression analysis obeying the Beer-Lamber law. All absorbance values are averages (n = 3). c) Fluorescence area *vs.* absorbance plot for the quantum yield comparative method. All absorbance and fluorescence area values are averages (n = 3).

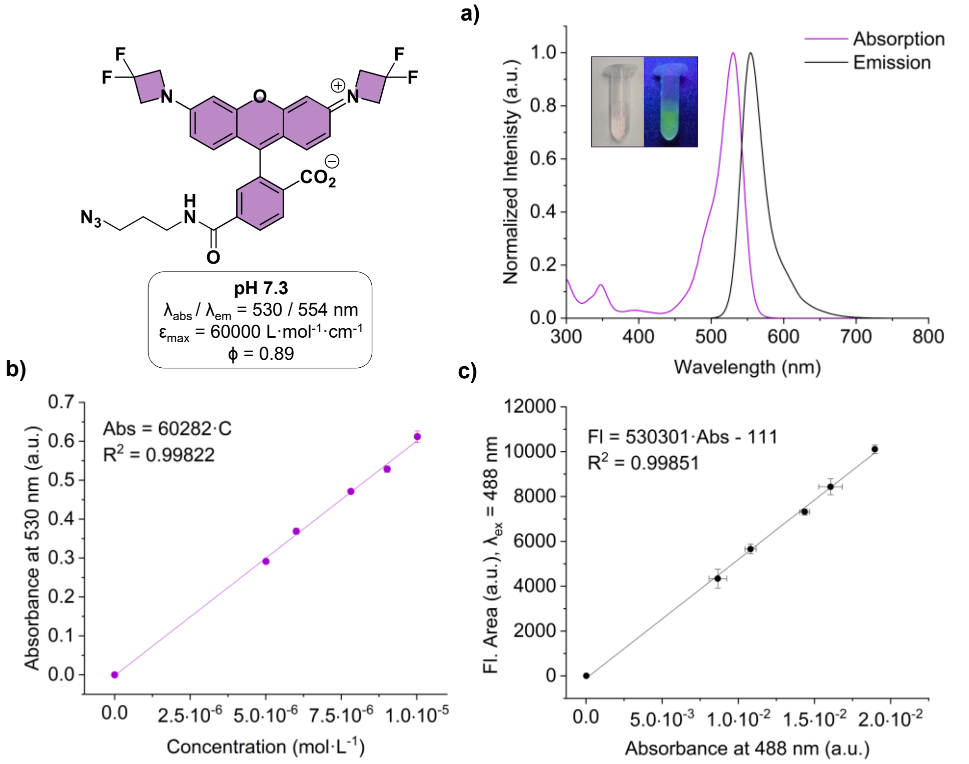

Figure S7. Photophysical properties of **RD$_{F2}$** (**12**) in 10 mM HEPES, pH 7.3 buffer at room temperature. a) Absorption and emission spectra. Solutions in the photographs correspond to **RD$_{F2}$** at 2 μM (absorbance; left) and 0.2 μM (emission; right). b) Linear regression analysis obeying the Beer-Lamber law. All absorbance values are averages (n = 3). c) Fluorescence area *vs.* absorbance plot for the quantum yield comparative method. All absorbance and fluorescence area values are averages (n = 3).

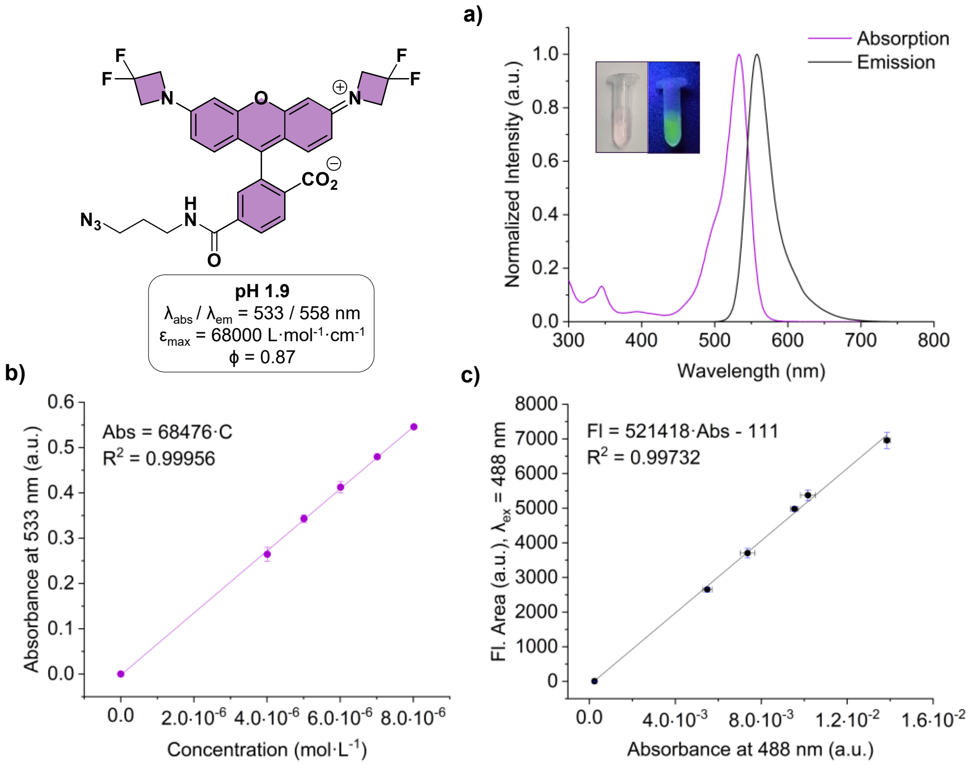

Figure S8. Photophysical properties of **RD$_{F2}$** (**12**) in aqueous TFA (0.1 % v/v) at room temperature. a) Absorption and emission spectra. Solutions in the photographs correspond to **RD$_{F2}$** at 2 μM (absorbance; left) and 0.2 μM (emission; right). b) Linear regression analysis obeying the Beer-Lamber law. All absorbance values are averages (n = 3). c) Fluorescence area *vs.* absorbance plot for the quantum yield comparative method. All absorbance and fluorescence area values are averages (n = 3).

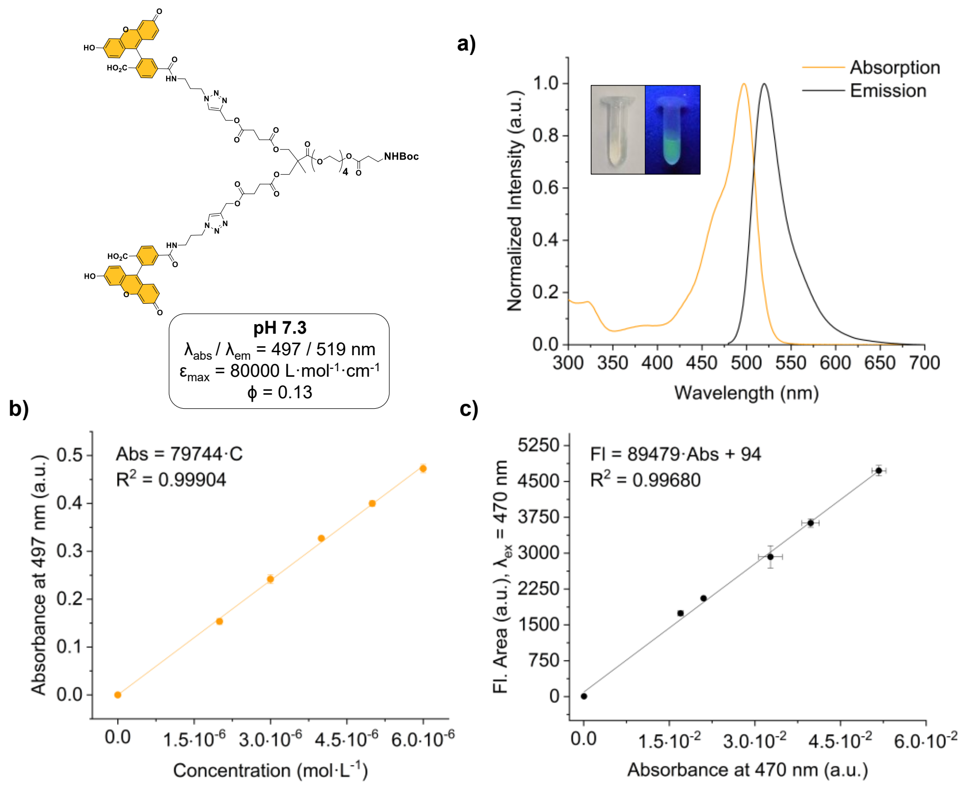

Figure S9. Photophysical properties of **FD2** (**13**) in 10 mM HEPES, pH 7.3 buffer at room temperature. a) Absorption and emission spectra. Solutions in the photographs correspond to **FD2** at 2 μM (absorbance; left) and 0.2 μM (emission; right). b) Linear regression analysis obeying the Beer-Lamber law. All absorbance values are averages (n = 3). c) Fluorescence area *vs.* absorbance plot for the quantum yield comparative method. All absorbance and fluorescence area values are averages (n = 3).

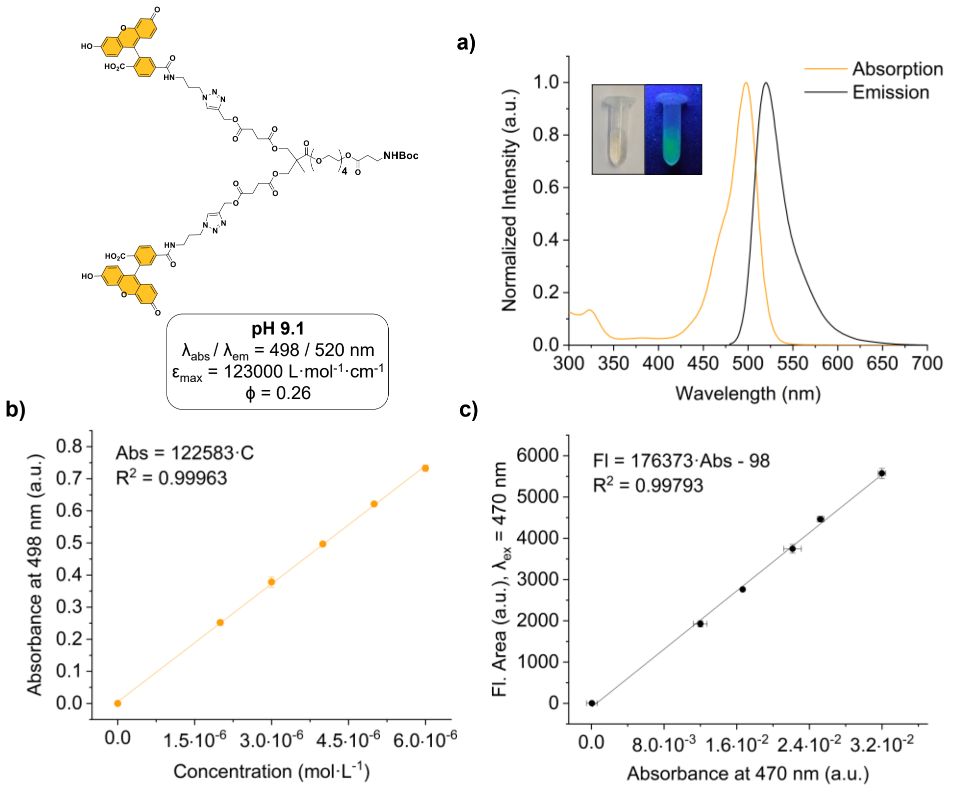

Figure S10. Photophysical properties of **FD2** (**13**) in 10 mM sodium borate, pH 9.1 buffer at room temperature. a) Absorption and emission spectra. Solutions in the photographs correspond to **FD2** at 2 μM (absorbance; left) and 0.2 μM (emission; right). b) Linear regression analysis obeying the Beer-Lamber law. All absorbance values are averages (n = 3). c) Fluorescence area *vs.* absorbance plot for the quantum yield comparative method. All absorbance and fluorescence area values are averages (n = 3).

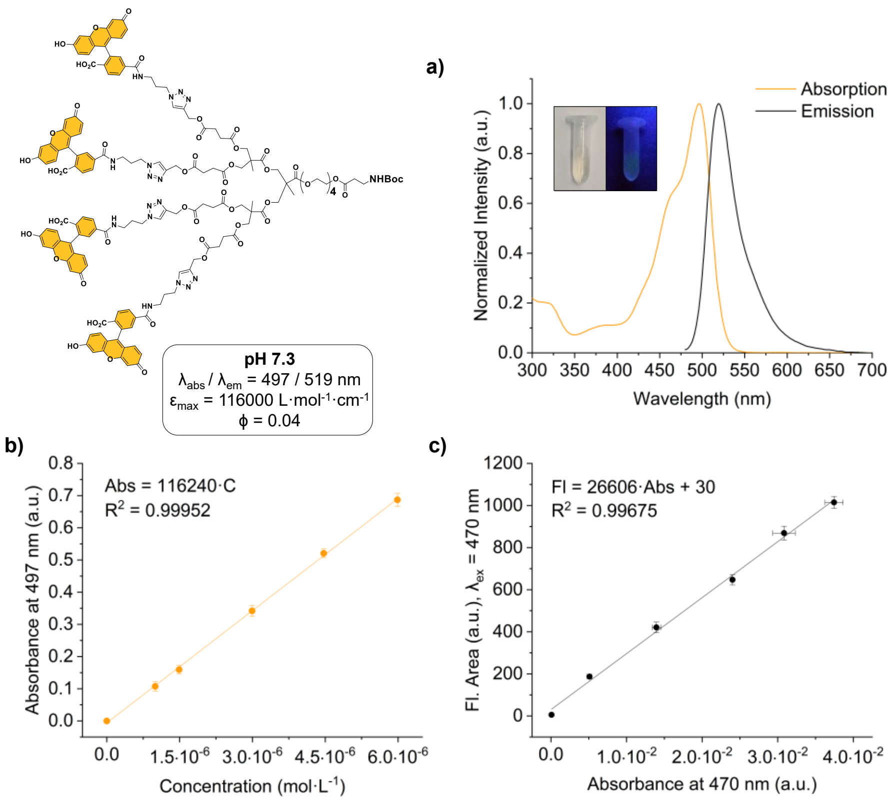

Figure S11. Photophysical properties of **FD4** (**14**) in 10 mM HEPES, pH 7.3 buffer at room temperature. a) Absorption and emission spectra. Solutions in the photographs correspond to **FD4** at 2 μM (absorbance; left) and 0.2 μM (emission; right). b) Linear regression analysis obeying the Beer-Lamber law. All absorbance values are averages (n = 3). c) Fluorescence area *vs.* absorbance plot for the quantum yield comparative method. All absorbance and fluorescence area values are averages (n = 3).

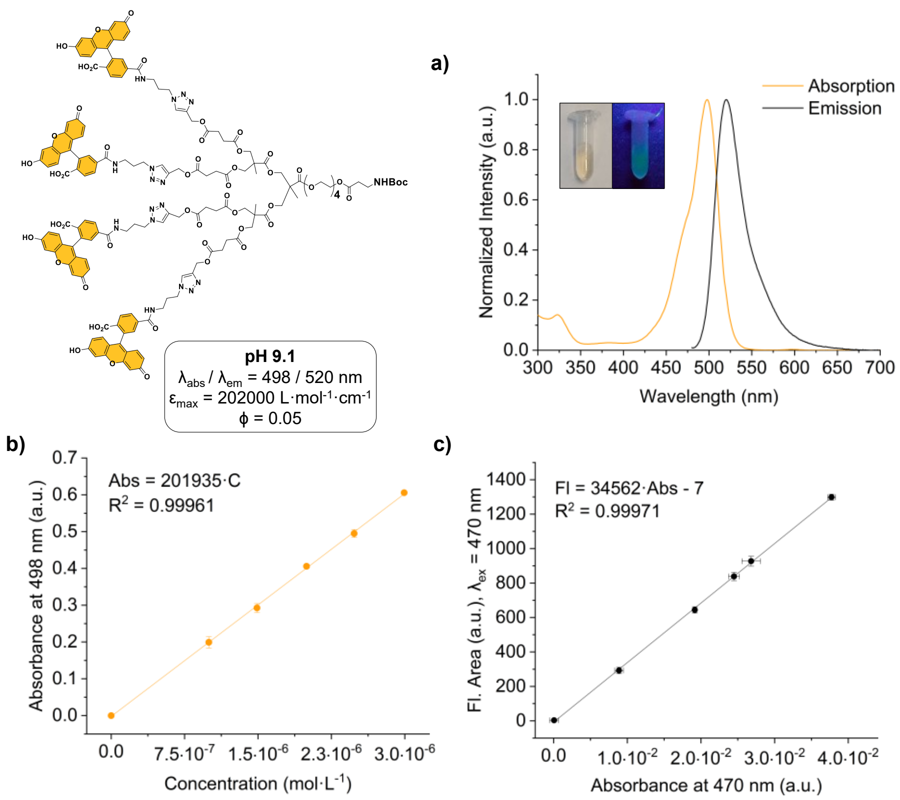

Figure S12. Photophysical properties of **FD4** (**14**) in 10 mM sodium borate, pH 9.1 buffer at room temperature. a) Absorption and emission spectra. Solutions in the photographs correspond to **FD4** at 2 μM (absorbance; left) and 0.2 μM (emission; right). b) Linear regression analysis obeying the Beer-Lamber law. All absorbance values are averages (n = 3). c) Fluorescence area *vs.* absorbance plot for the quantum yield comparative method. All absorbance and fluorescence area values are averages (n = 3).

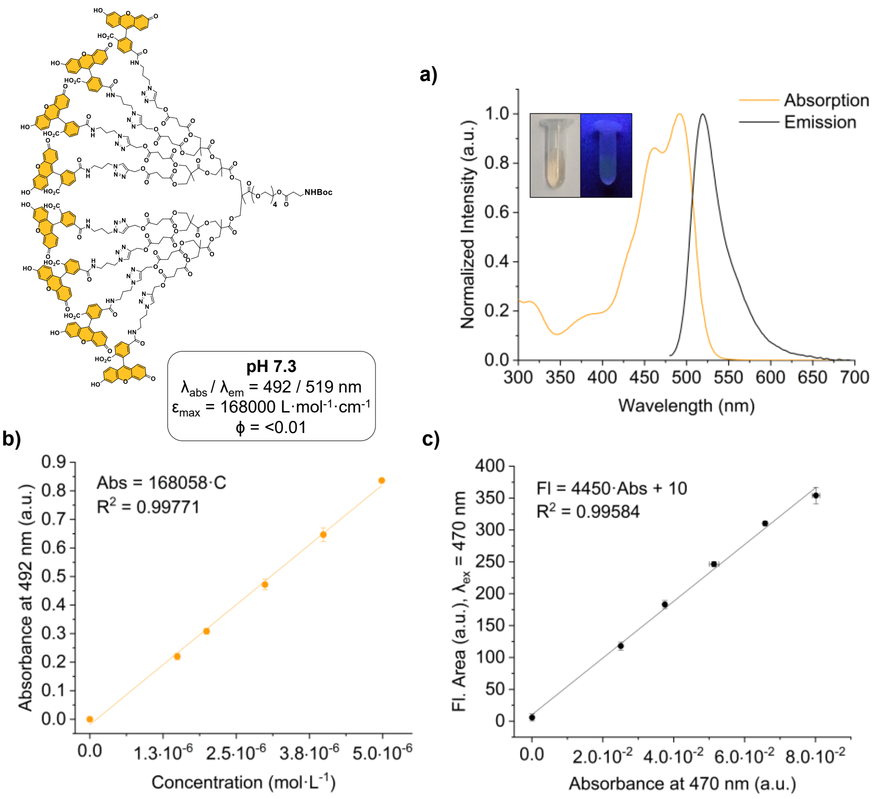

Figure S13. Photophysical properties of **FD8** (**15**) in 10 mM HEPES, pH 7.3 buffer at room temperature. a) Absorption and emission spectra. Solutions in the photographs correspond to **FD8** at 2 µM (absorbance; left) and 0.2 µM (emission; right). b) Linear regression analysis obeying the Beer-Lamber law. All absorbance values are averages (n = 3). c) Fluorescence area *vs.* absorbance plot for the quantum yield comparative method. All absorbance and fluorescence area values are averages (n = 3).

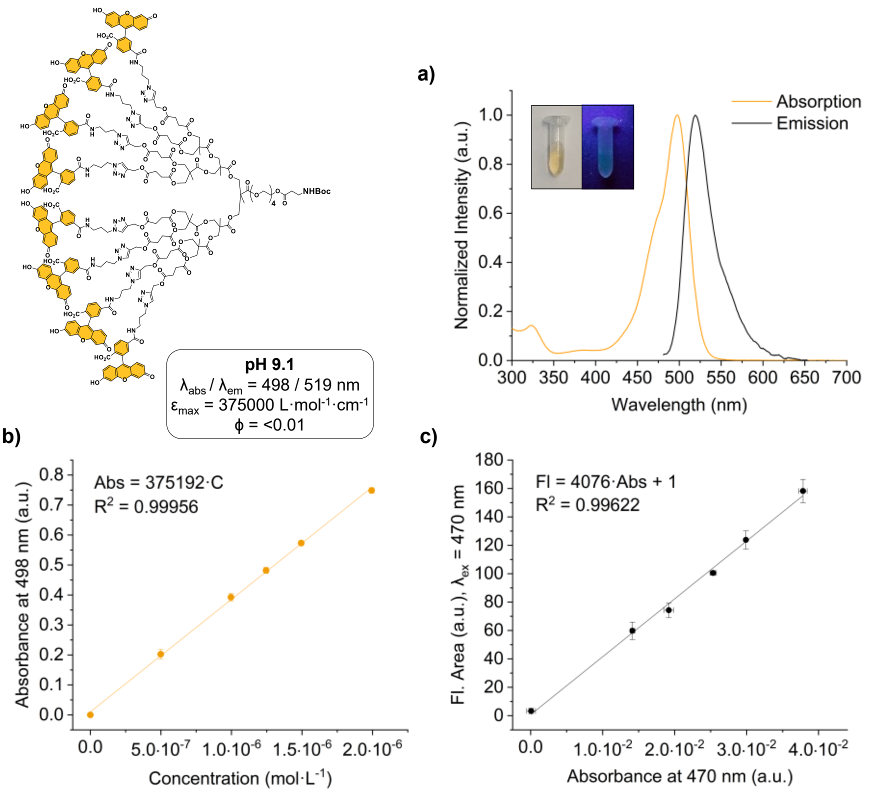

Figure S14. Photophysical properties of **FD8** (**15**) in 10 mM sodium borate, pH 9.1 buffer at room temperature. a) Absorption and emission spectra. Solutions in the photographs correspond to **FD8** at 2 µM (absorbance; left) and 0.2 µM (emission; right). b) Linear regression analysis obeying the Beer-Lamber law. All absorbance values are averages (n = 3). c) Fluorescence area *vs.* absorbance plot for the quantum yield comparative method. All absorbance and fluorescence area values are averages (n = 3).

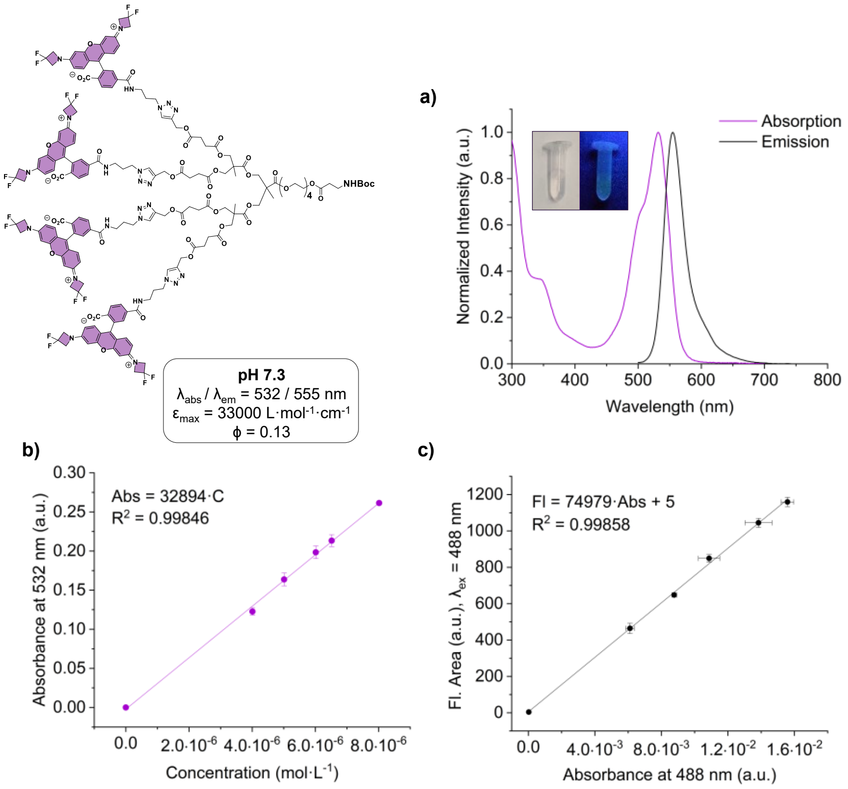

Figure S15. Photophysical properties of **RD$_{F2}$D4** (**17**) in 10 mM HEPES, pH 7.3 buffer at room temperature. a) Absorption and emission spectra. Solutions in the photographs correspond to **RD$_{F2}$D4** at 2 µM (absorbance; left) and 0.2 µM (emission; right). b) Linear regression analysis obeying the Beer-Lamber law. All absorbance values are averages (n = 3). c) Fluorescence area *vs.* absorbance plot for the quantum yield comparative method. All absorbance and fluorescence area values are averages (n = 3).

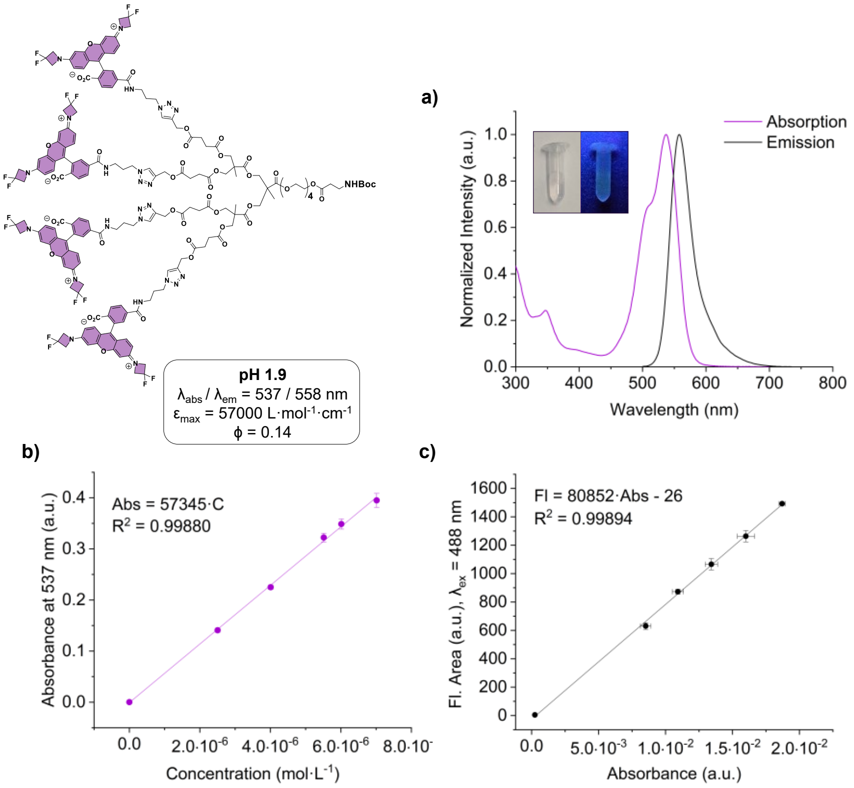

Figure S16. Photophysical properties of **RD$_{F2}$D4** (**17**) in aqueous TFA (0.1 % v/v) at room temperature. a) Absorption and emission spectra. Solutions in the photographs correspond to **RD$_{F2}$D4** at 2 µM (absorbance; left) and 0.2 µM (emission; right). b) Linear regression analysis obeying the Beer-Lamber law. All absorbance values are averages (n = 3). c) Fluorescence area *vs.* absorbance plot for the quantum yield comparative method. All absorbance and fluorescence area values are averages (n = 3).

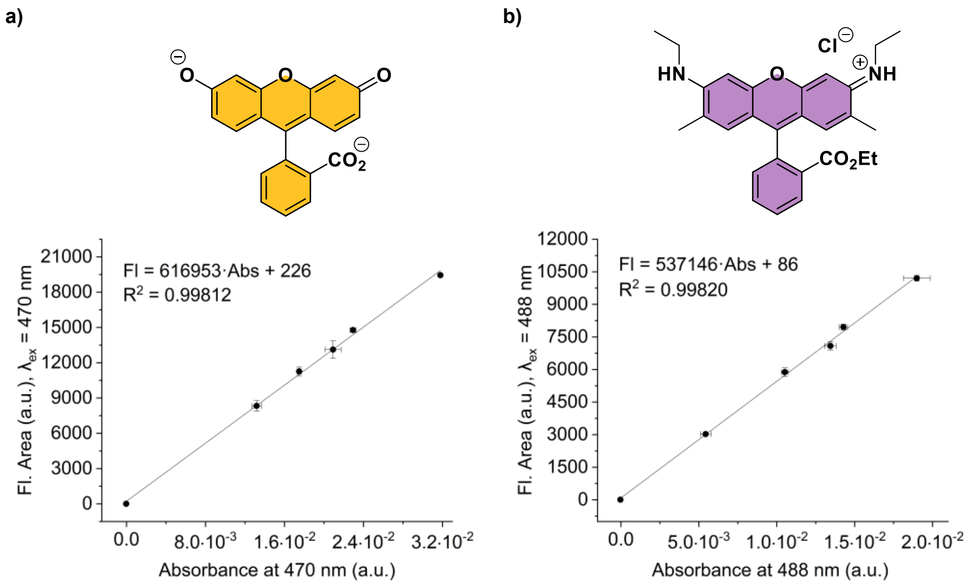

Figure S17. Fluorescence area *vs.* absorbance plots of the references used for the quantum yield comparative method. a) Fluorescein in aqueous 0.1 M NaOH. b) Rhodamine 6G in ethanol. All absorbance and fluorescence area values are averages (n = 3) and the measurements were taken at room temperature.

4. Photostability Studies of RD_{H2}, RD_m and RD_{F2}

The photobleaching rate constant values (*k*) and fluorescence half-life time values ($t_{1/2}$) of **RD_{H2}**, **RD_m**, and **RD_{F2}** were evaluated to characterise the photostability of these new dyes. As described in the General Experimental Methods section, ln $(\frac{I}{I_0})$ was plotted against the irradiation time (t) (**Equation 1**) to obtain the photobleaching constant rate as the absolute value of the slope's linear fitting (see **Table S1** and **Figure S18**). Fluorescence half-life time values were calculated, therefore, based on the photobleaching constant rates (**Equation 2**). The photostability of **TAMRA-PEG3-azide** and **fluorescein azide** was also evaluated for comparative reasons. As shown in **Table S1**, the newly synthesised rhodamine dyes exhibit similar fluorescence half-life times to that of **TAMRA-PEG3-azide**, demonstrating the photostability of the new dyes is comparable to that of a commercially available rhodamine dye. The half-life time of **fluorescein azide** was also calculated to show, as already reported,[2,3] the exceptional photostability of rhodamine dyes in comparison to fluorescein.

Table S1. Photobleaching rate constants (*k*) and fluorescence half-life times ($t_{1/2}$) of **RD_{H2}**, **RD_m**, **RD_{F2}**, **TAMRA-PEG3-azide** and **fluorescein azide**.

Fluorophore	*k* (min^{-1})	$t_{1/2}$ (min)
**RD_{H2}**[a]	2.15×10^{-2}	32.3
**RD_m**[b]	4.89×10^{-2}	14.2
**RD_{F2}**[b]	2.75×10^{-2}	25.2
**TAMRA-PEG3-azide**[a]	2.39×10^{-2}	29.0
**Fluorescein azide**[c]	8.60×10^{-2}	8.1

[a] 0.2 μM sample irradiated using a 560 nm laser at a power density of 550 mW/cm^2. [b] 0.2 μM sample irradiated using a 531 nm laser at a power density of 550 mW/cm^2. [c] 0.2 μM sample irradiated using a 488 nm laser at a power density of 13 mW/cm^2. **TAMRA-PEG3-azide** and **fluorescence azide** were used as references for comparison.

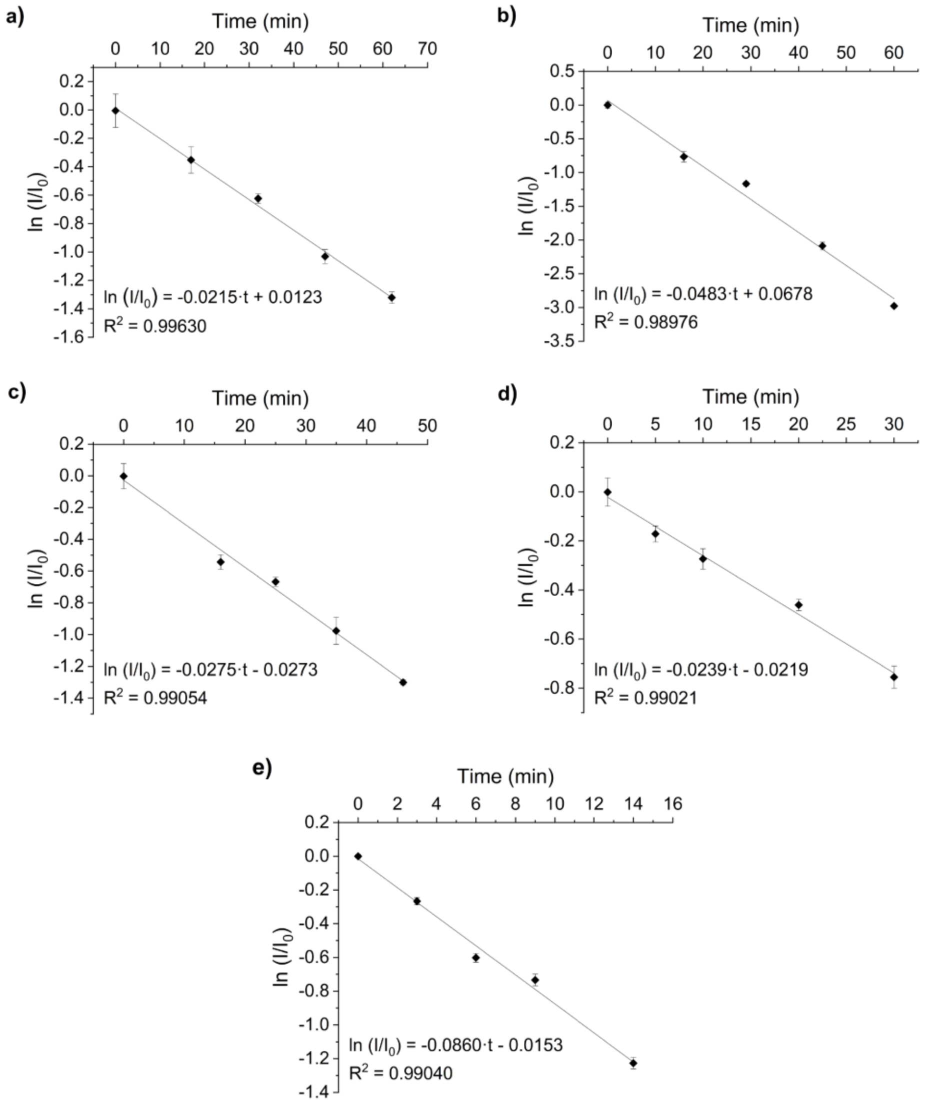

Figure S18. $\ln(I_0/I)$ plotted against irradiation time (t) of a) **RD$_{H2}$**, b) **RD$_m$**, c) **RD$_{F2}$**, d) **TAMRA-PEG3-azide** and e) **fluorescein azide**. The absolute value of the linear fitting's slope corresponds to the photobleaching rate constant (*k*). I_0 = initial intensity. I = intensity at time *t*. All $\ln(I_0/I)$ values are averages (n = 3).

5. pK_a Values Calculation of RD_{H2}, RD_m and RD_{F2}

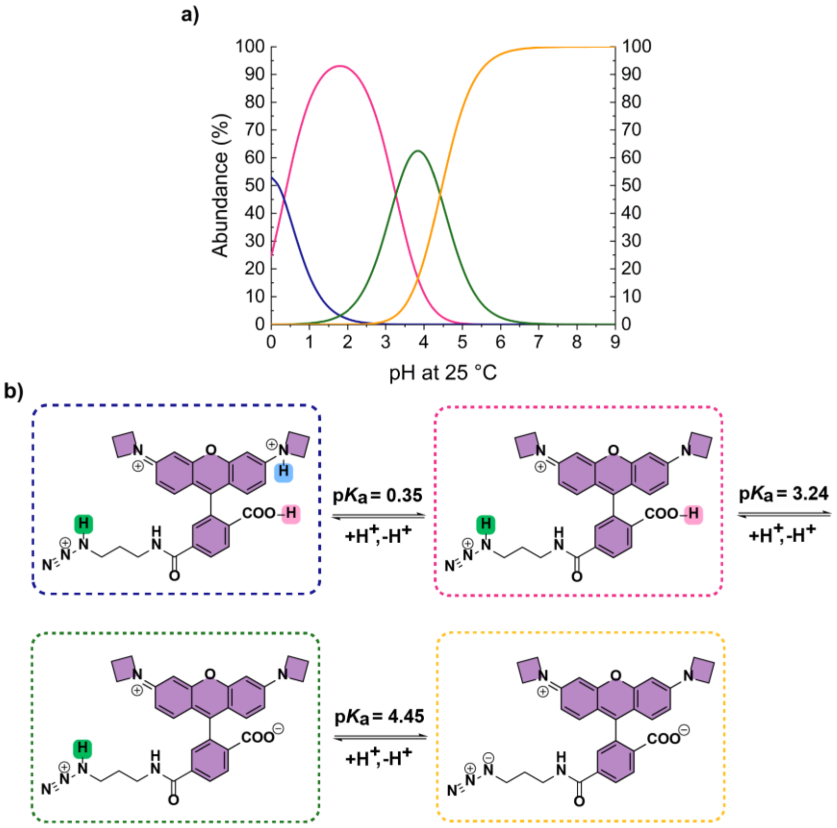

Figure S19. Calculation of the different pK_a values of $\mathbf{RD_{H2}}$ at 25 °C. a) Distribution of the different species in solution as a function of pH. b) Structure of the different protonated and unprotonated forms of $\mathbf{RD_{H2}}$. At pH 7.3 the main species found in solution is the deprotonated form of $\mathbf{RD_{H2}}$, whereas the doubly protonated form of $\mathbf{RD_{H2}}$ is the prevailing one at pH 1.9.

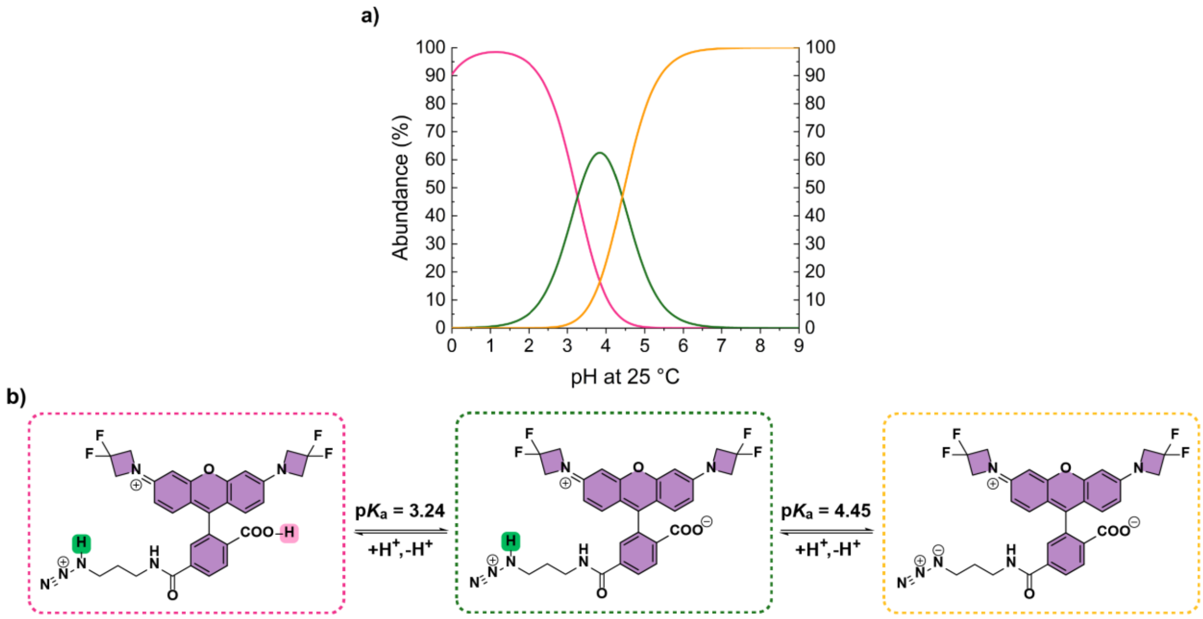

Figure S20. Calculation of the different pK_a values of $\mathbf{RD_{F2}}$ at 25 °C. a) Distribution of the different species in solution as a function of pH. b) Structure of the different protonated and unprotonated forms of $\mathbf{RD_{F2}}$. At pH 7.3 the main species found in solution is unprotonated form of RD_{F2}, whereas the doubly protonated form of RD_{F2} is the prevailing one at pH 1.9.

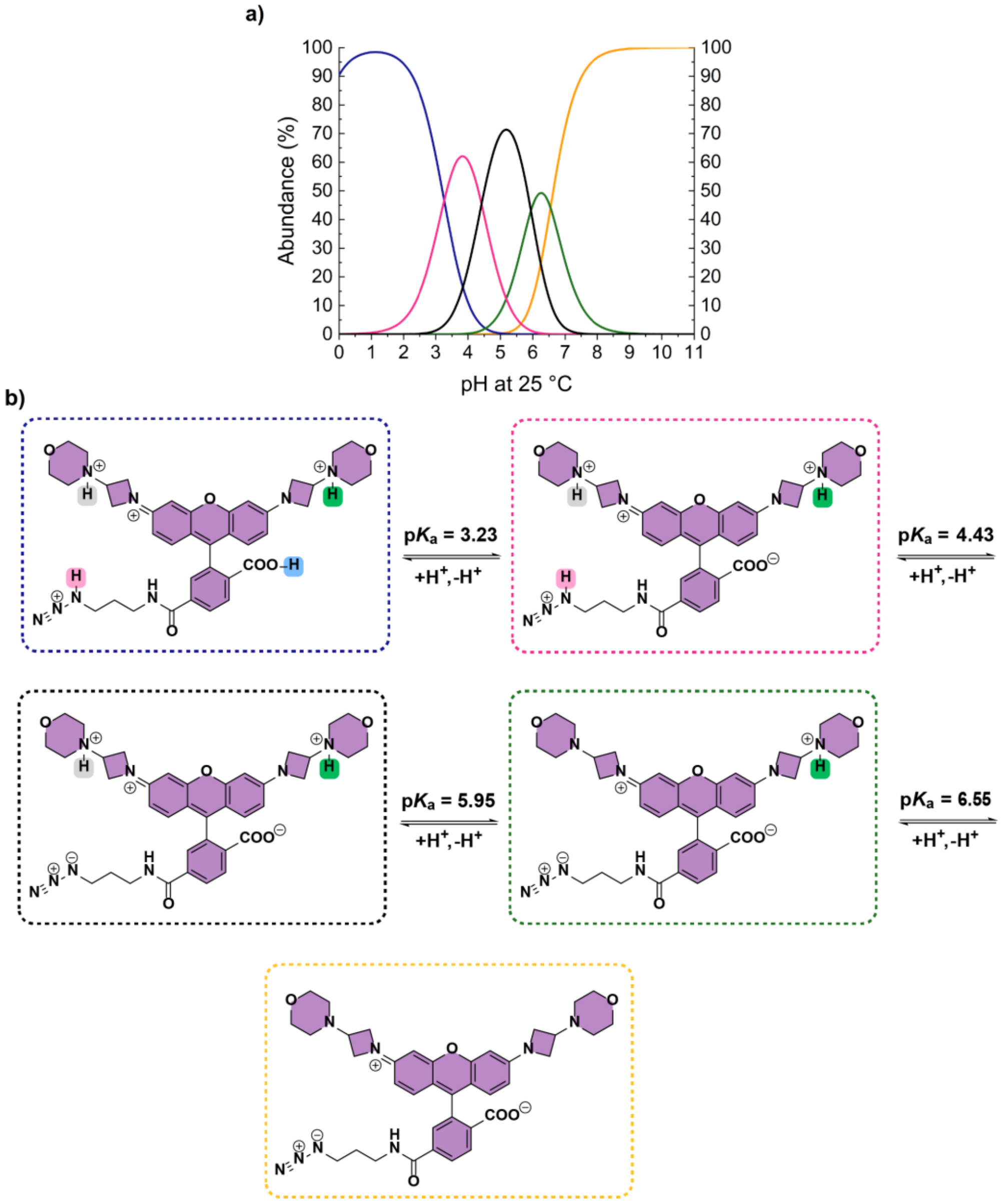

Figure S21. Calculation of the different pK_a values of **RD$_m$** at 25 °C. a) Distribution of the different species in solution as a function of pH. b) Structure of the different protonated and unprotonated forms of **RD$_m$**. At pH 7.3 the main species found in solution are the unprotonated and monoprotonated form of **RD$_m$**, whereas the tetraprotonated form of **RD$_m$** is the prevailing one at pH 1.9.

6. Solution Stability Studies

A solution stability study was performed for **fluorescein azide**, **FD2**, **FD4**, **FD8**, **RD$_{H2}$**, **RD$_m$**, **RD$_{F2}$** and **RD$_{F2}$D4**. In order to carry out this experiment, buffered pH 7.3 aqueous solutions (HEPES, 10 mM) of the fluorophores were prepared at a concentration of 2.0 µM. The samples were allowed to stand under ambient conditions for 7 days, at room temperature and shielded from sunlight. Aliquots were taken and measured by HPLC at three time points, shown below. For solubility reasons, analyses of **RD$_{F2}$D4** by HPLC were not possible and, consequently, MALDI-ToF measurements were performed. Overall, all fluorophores are stable after 1 week in solution under these conditions.

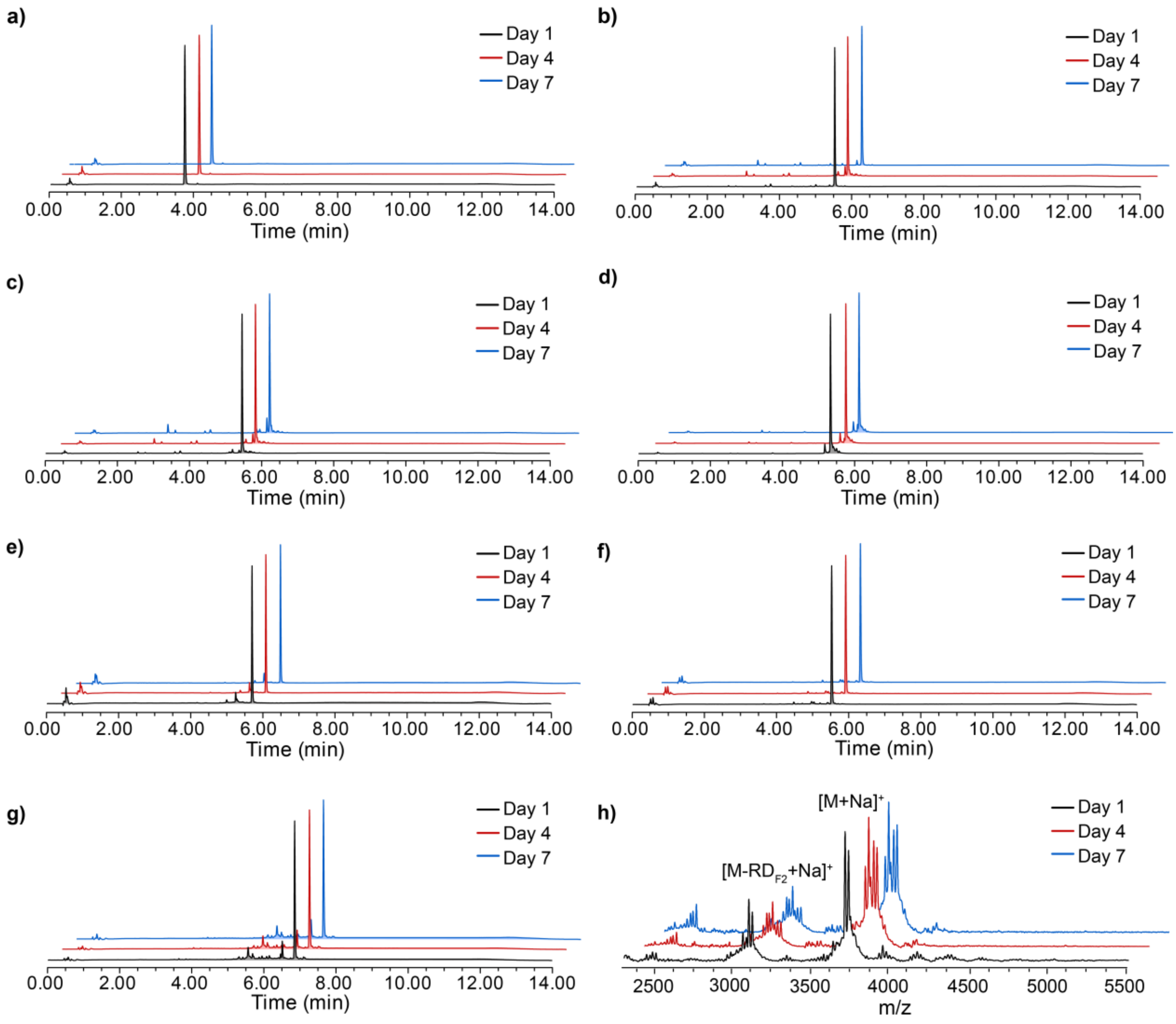

Figure S22. Solution stability study of a) **fluorescein azide**, b) **FD2**, c) **FD4**, d) **FD8**, e) **RD$_{H2}$**, f) **RD$_m$**, g) **RD$_{F2}$** and h) **RD$_{F2}$D4**.,HPLC run conditions: a-f) 1.5 mL/min, 40 °C, buffer B 0-30 % v/v 0 → 4 min, then 30-85 % v/v 4 → 10 min. g) 1.5 mL/min, 40 °C, buffer B 0-30 % v/v 0 → 4 min, then 30-85 % v/v 4 → 6 min and 85 % v/v 6 → 10 min. MALDI-ToF conditions: h) positive ionization mode, DCTB (matrix), NaTFA (cationization agent).

7. Mass Spectra of the Synthesised Compounds

7.1 ESI spectra

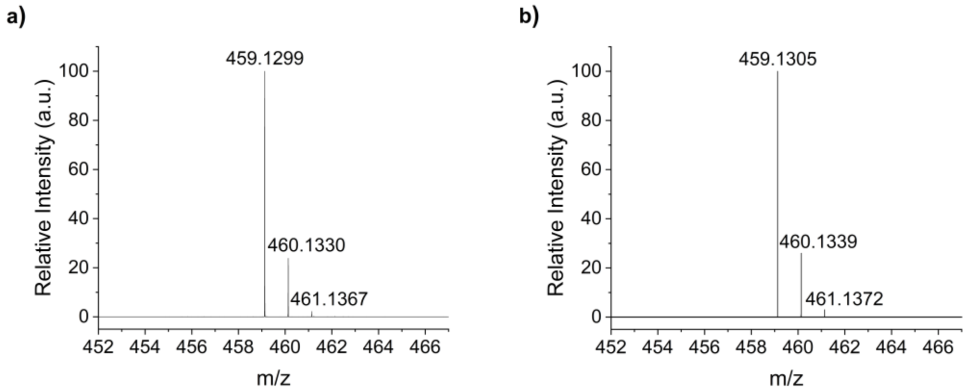

Figure S23. a) Experimental and b) theoretical isotopic distributions of $[M+H]^+$ for **fluorescein azide**.

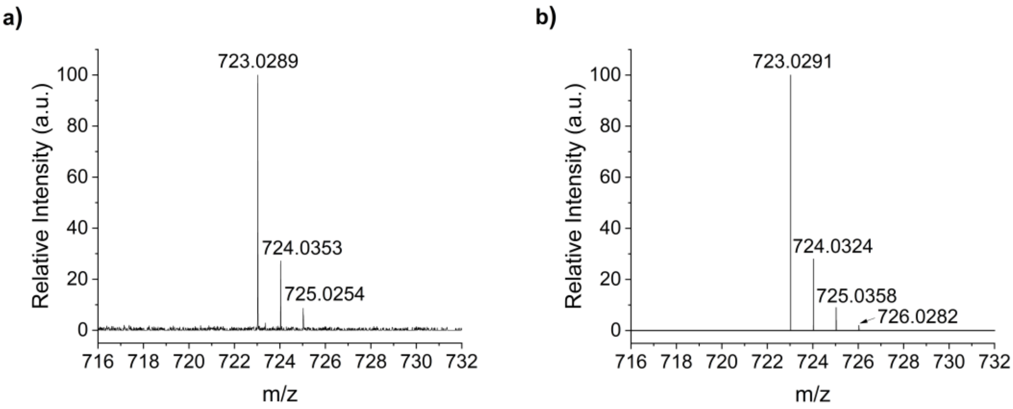

Figure S24. a) Experimental and b) theoretical isotopic distributions of $[M+H]^+$ for **fluorescein ditriflate**.

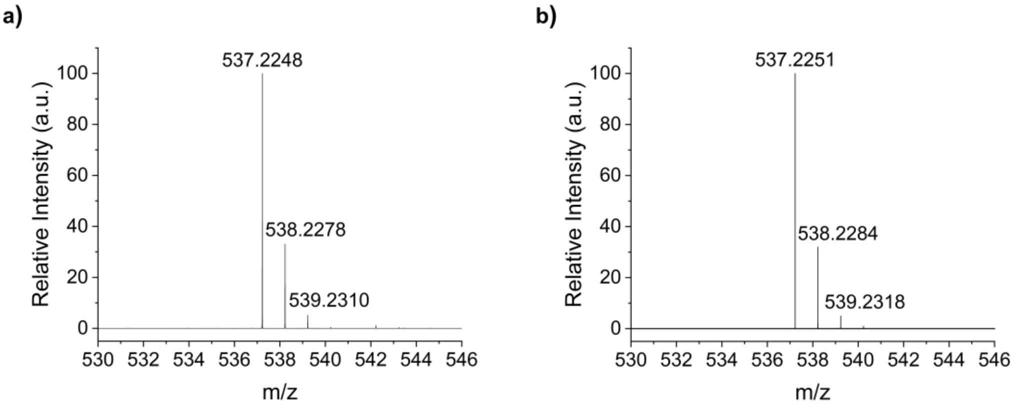

Figure S25. a) Experimental and b) theoretical isotopic distributions of $[M+H]^+$ for **RD$_{H2}$**.

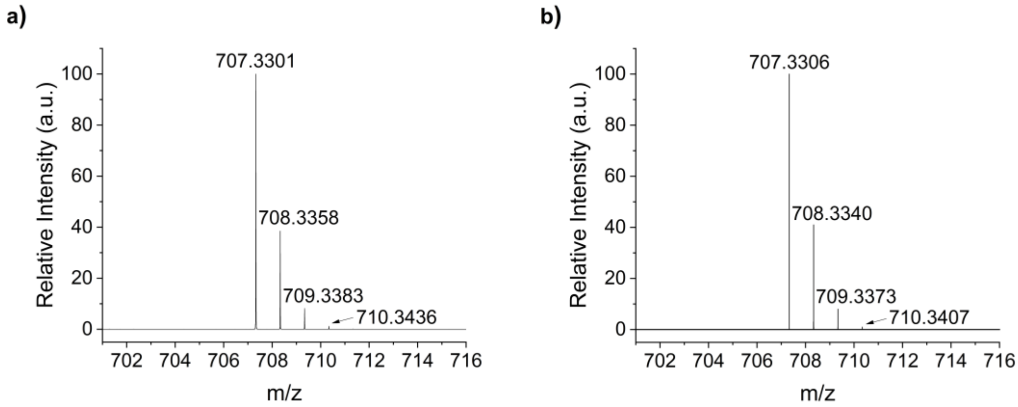

Figure S26. Experimental and b) theoretical isotopic distributions of $[M+H]^+$ for **RD$_m$**.

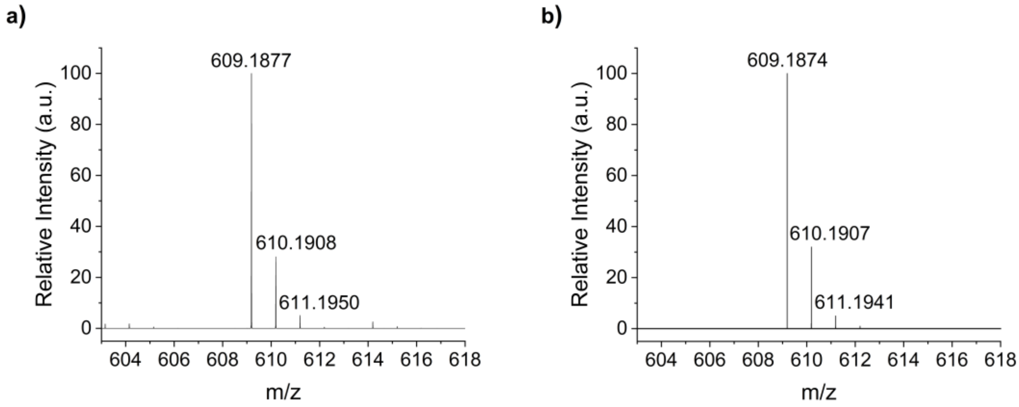

Figure S27. Experimental and b) theoretical isotopic distributions of $[M+H]^+$ for **RD$_{F2}$**.

7.2 MALDI-ToF spectra

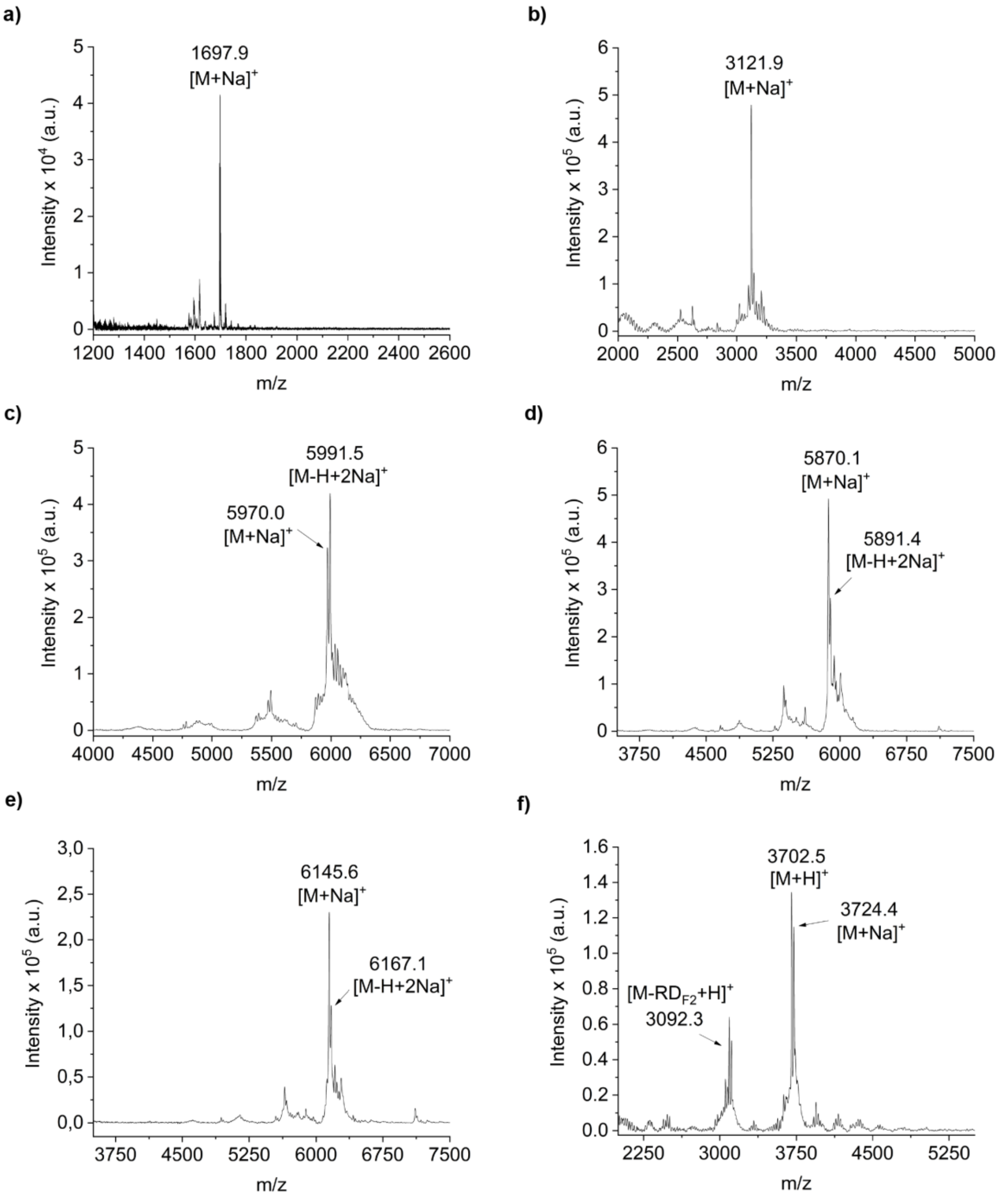

Figure S28. MALDI-ToF spectra of a) **FD2**, b) **FD4**, c) **FD8**, d) **FD8-NH$_3$**, e) **FD8-N$_3$** and f) **RD$_{F2}$D4.**

8. NMR Spectra of the Synthesised Compounds

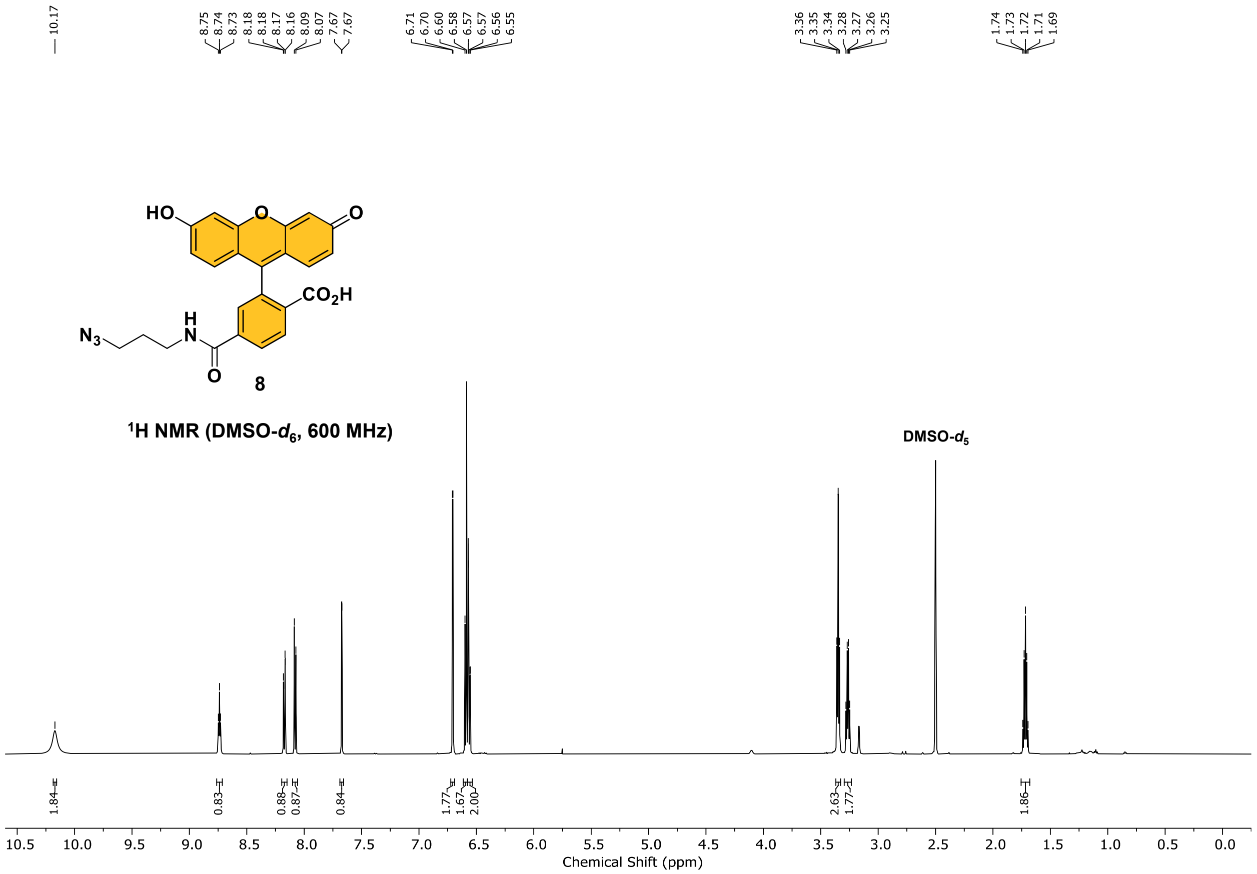

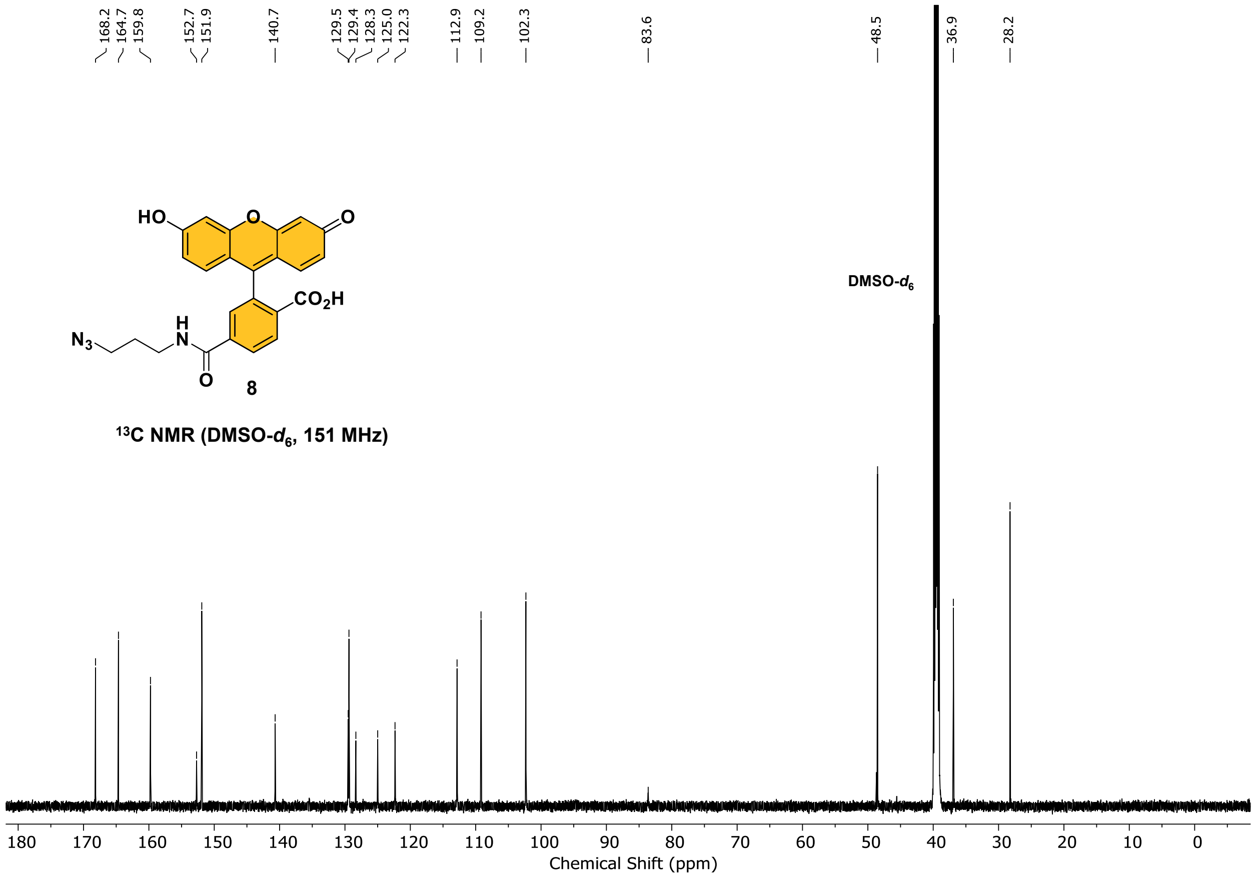

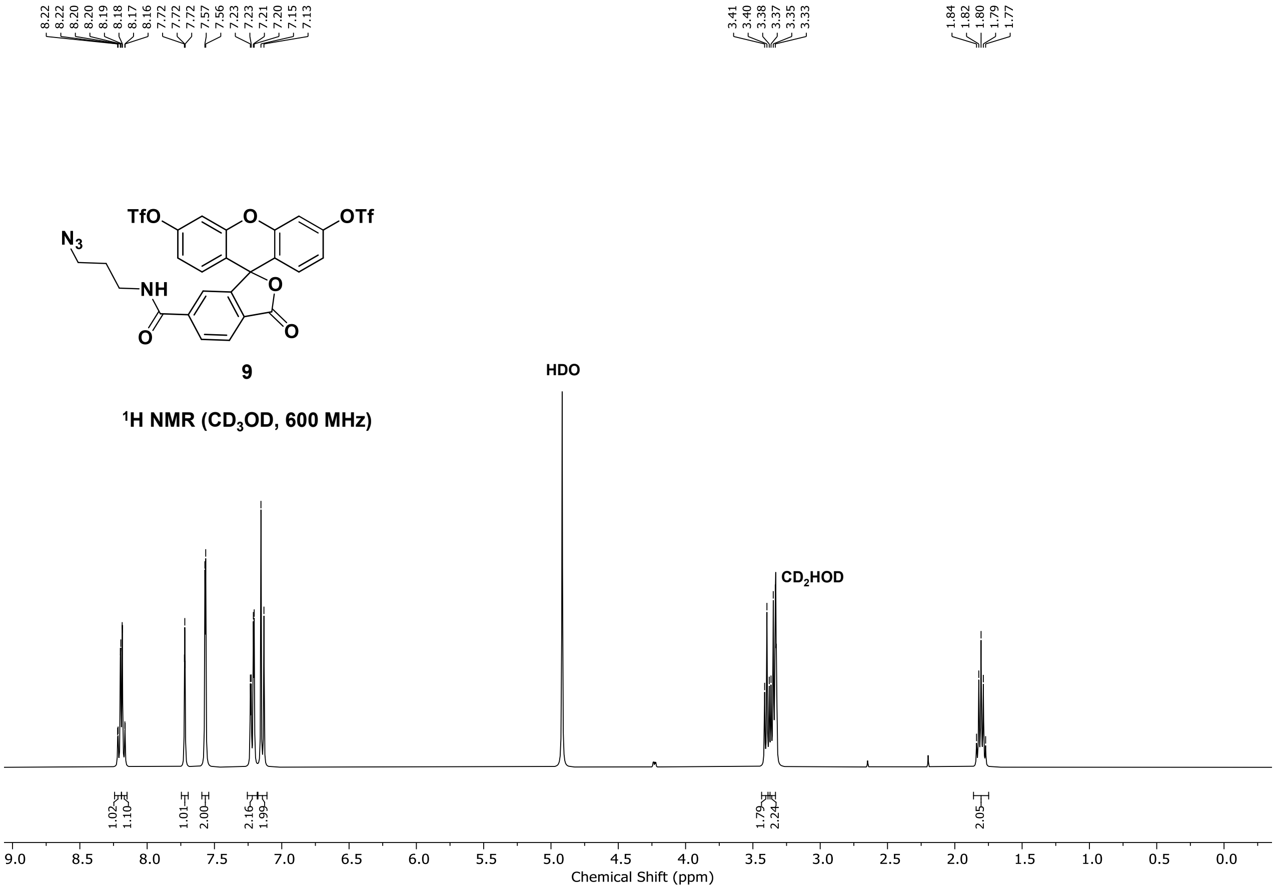
8.22 8.22 8.20 8.20 8.19 8.18 8.17 8.16 7.72 7.72 7.72 7.72 7.57 7.56 7.23 7.23 7.21 7.20 7.15 7.13
3.41 3.40 3.38 3.37 3.35 3.33
1.84 1.82 1.80 1.79 1.77
TfO
O
OTf
N3
NH
O
O
O
9
1H NMR (CD3OD, 600 MHz)
HDO
CD2HOD
1.02 1.10 1.01 2.00 2.16 1.99 1.79 2.24 2.05
Chemical Shift (ppm)

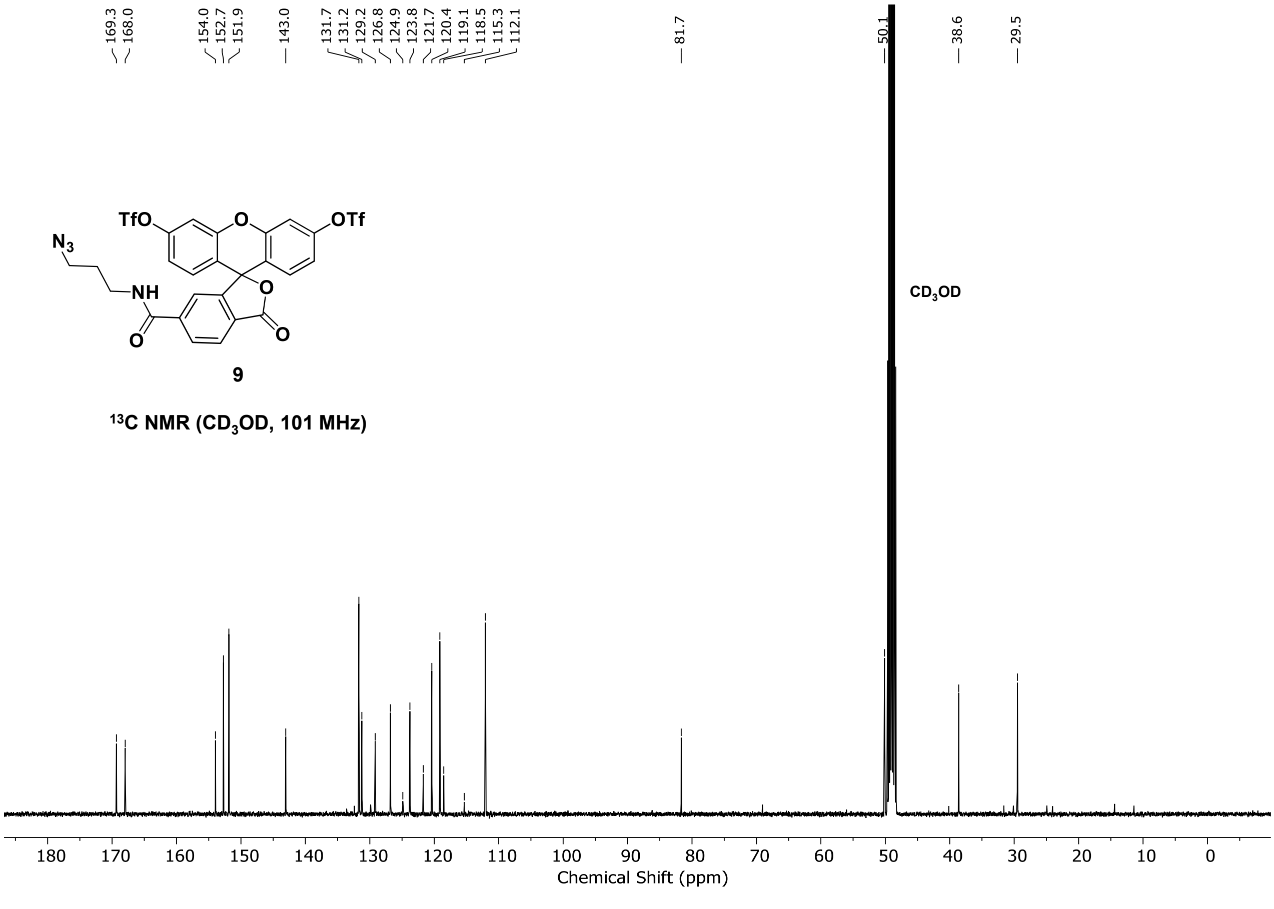
169.3 168.0 154.0 152.7 151.9 143.0 131.7 131.2 129.2 126.8 124.9 123.8 121.7 120.4 119.1 118.5 115.3 112.1 81.7 50.1 38.6 29.5
TfO
O
OTf
N3
NH
O
O
O
9
13C NMR (CD3OD, 101 MHz)
CD3OD
Chemical Shift (ppm)

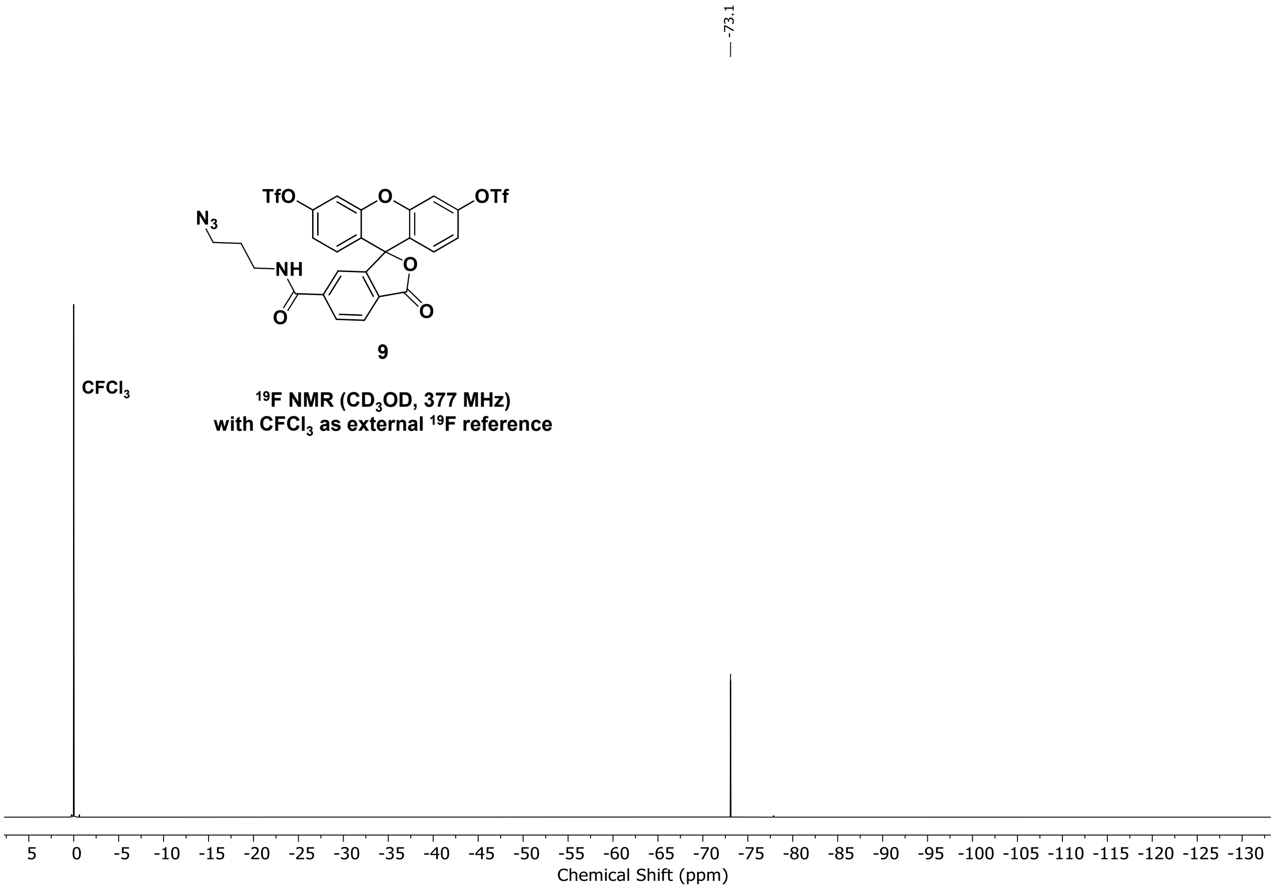

-73.1
TfO
OTf
N3
NH
O
9
CFCl3
19F NMR (CD3OD, 377 MHz)
with CFCl3 as external 19F reference
Chemical Shift (ppm)

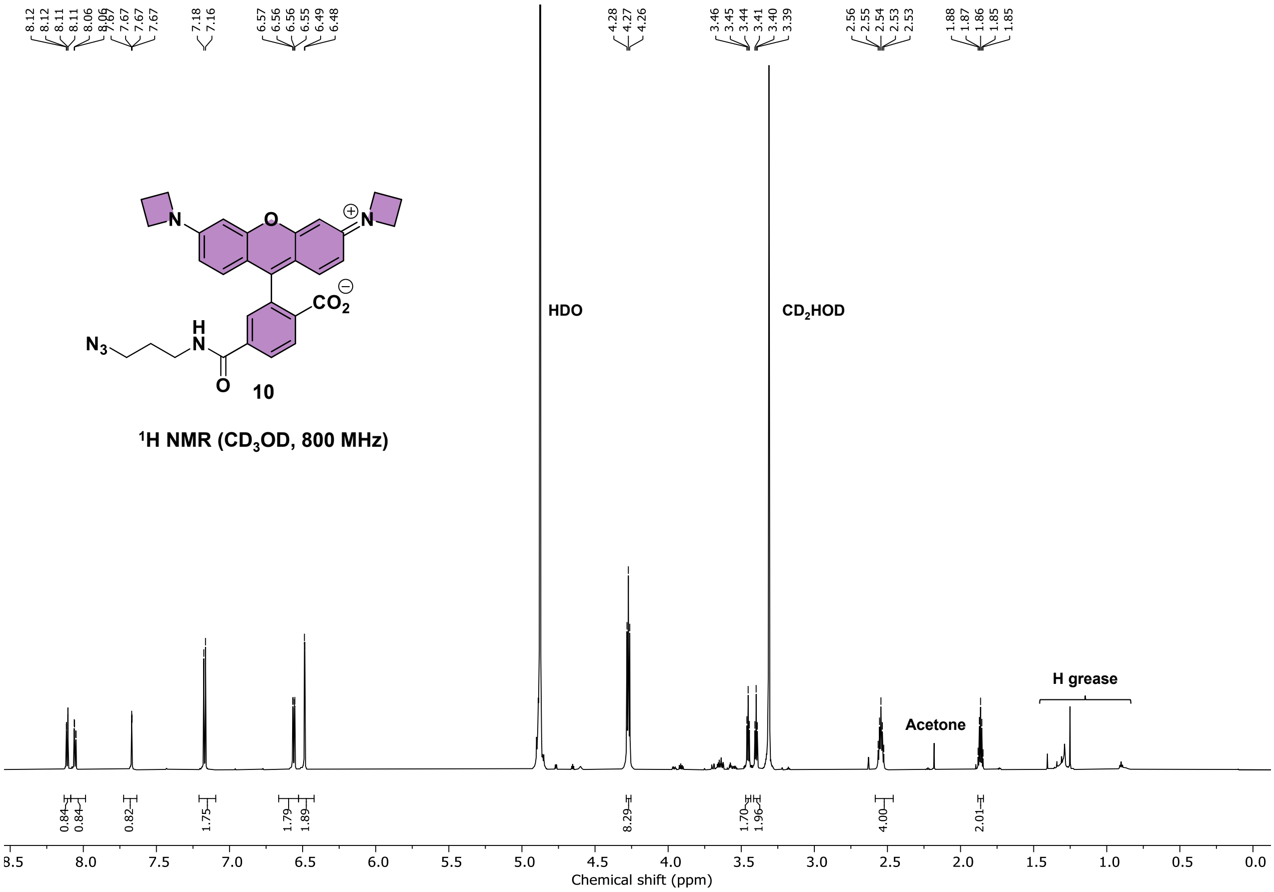

N
O
N
CO2
H
N
N3
O
10
1H NMR (CD3OD, 800 MHz)
HDO
CD2HOD
Acetone
H grease
Chemical shift (ppm)

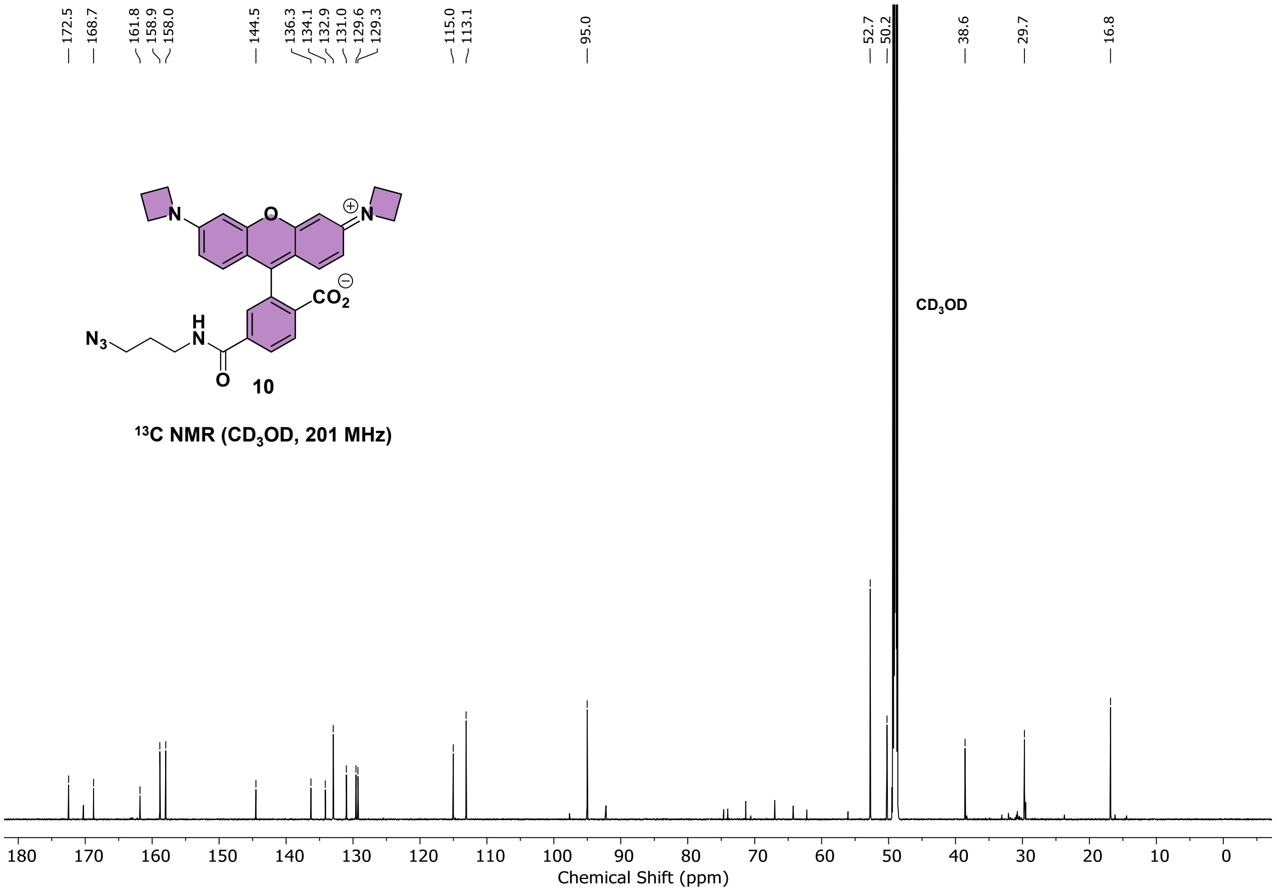
172.5
168.7
161.8
158.9
158.0
144.5
136.3
134.1
132.9
131.0
129.6
129.3
115.0
113.1
95.0
52.7
50.2
38.6
29.7
16.8
N
O
N
CO2
N3
H
N
O
10
13C NMR (CD3OD, 201 MHz)
CD3OD
Chemical Shift (ppm)

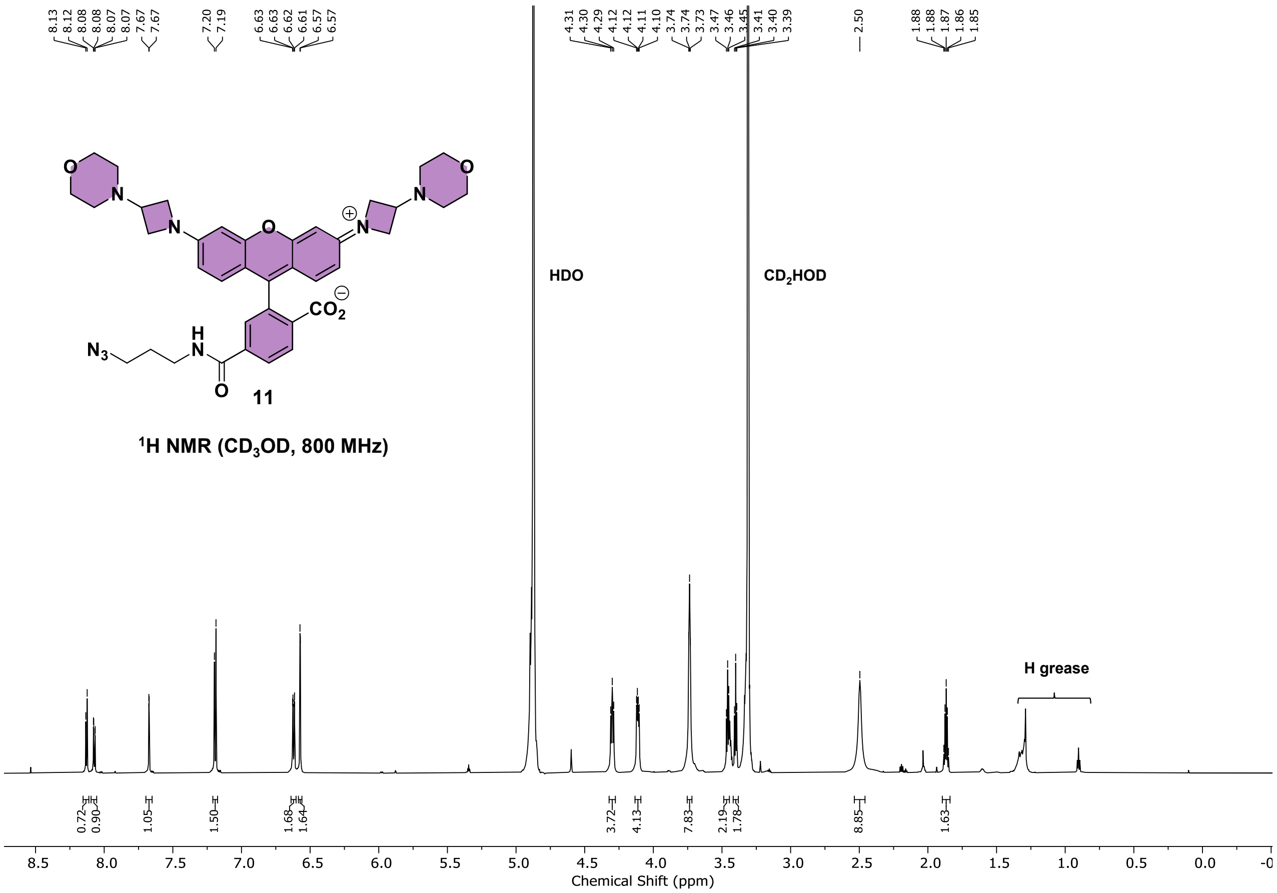
8.13
8.12
8.08
8.08
8.07
7.67
7.67
7.20
7.19
6.63
6.63
6.62
6.61
6.57
6.57
4.31
4.30
4.29
4.12
4.12
4.11
4.10
3.74
3.74
3.73
3.47
3.46
3.45
3.41
3.40
3.39
2.50
1.88
1.88
1.87
1.86
1.85
O
N
N
O
N
N
O
CO2
N3
H
N
O
11
1H NMR (CD3OD, 800 MHz)
HDO
CD2HOD
H grease
0.72
0.90
1.05
1.50
1.68
1.64
3.72
4.13
7.83
2.19
1.78
8.85
1.63
Chemical Shift (ppm)

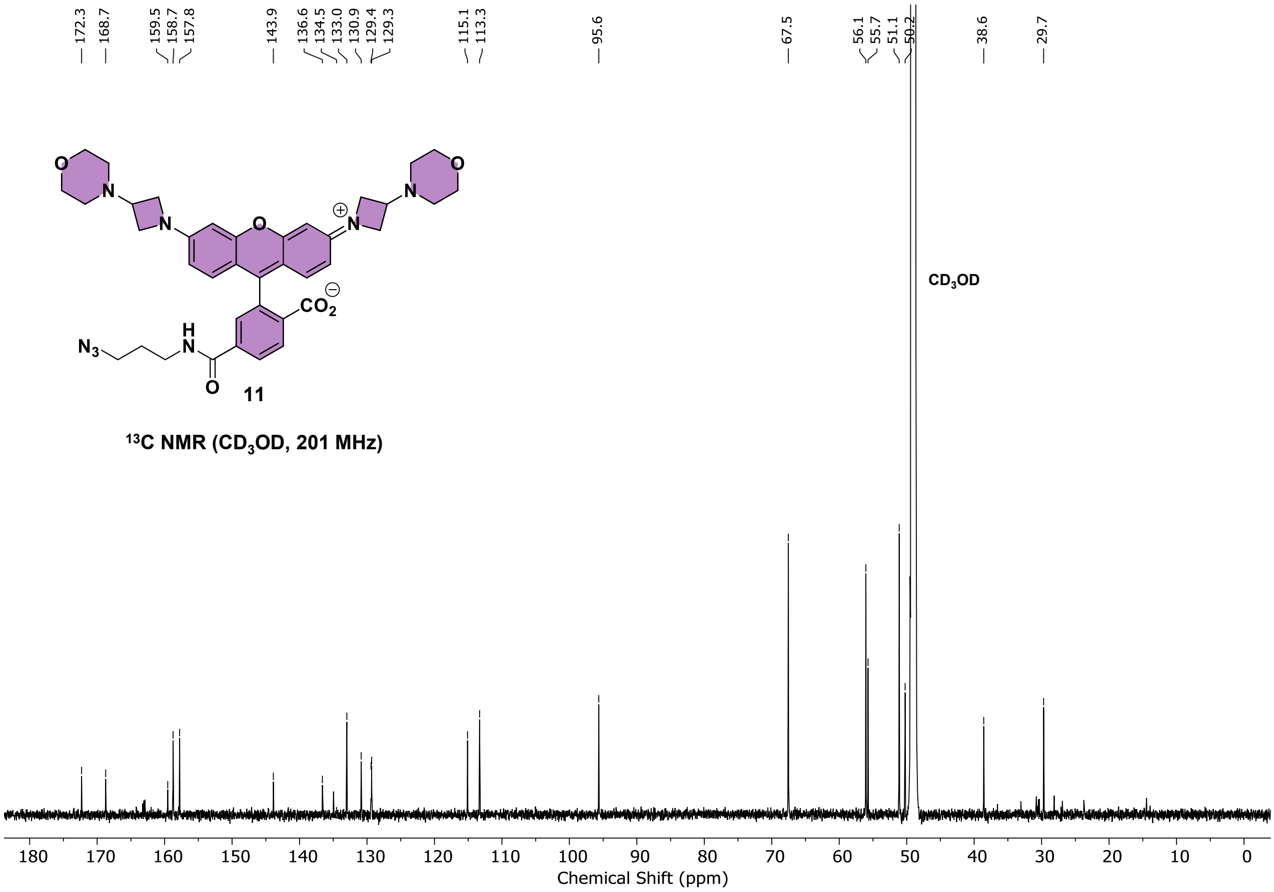
172.3
168.7
159.5
158.7
157.8
143.9
136.6
134.5
133.0
130.9
129.4
129.3
115.1
113.3
95.6
67.5
56.1
55.7
51.1
50.2
38.6
29.7
CD3OD
11
13C NMR (CD3OD, 201 MHz)
Chemical Shift (ppm)

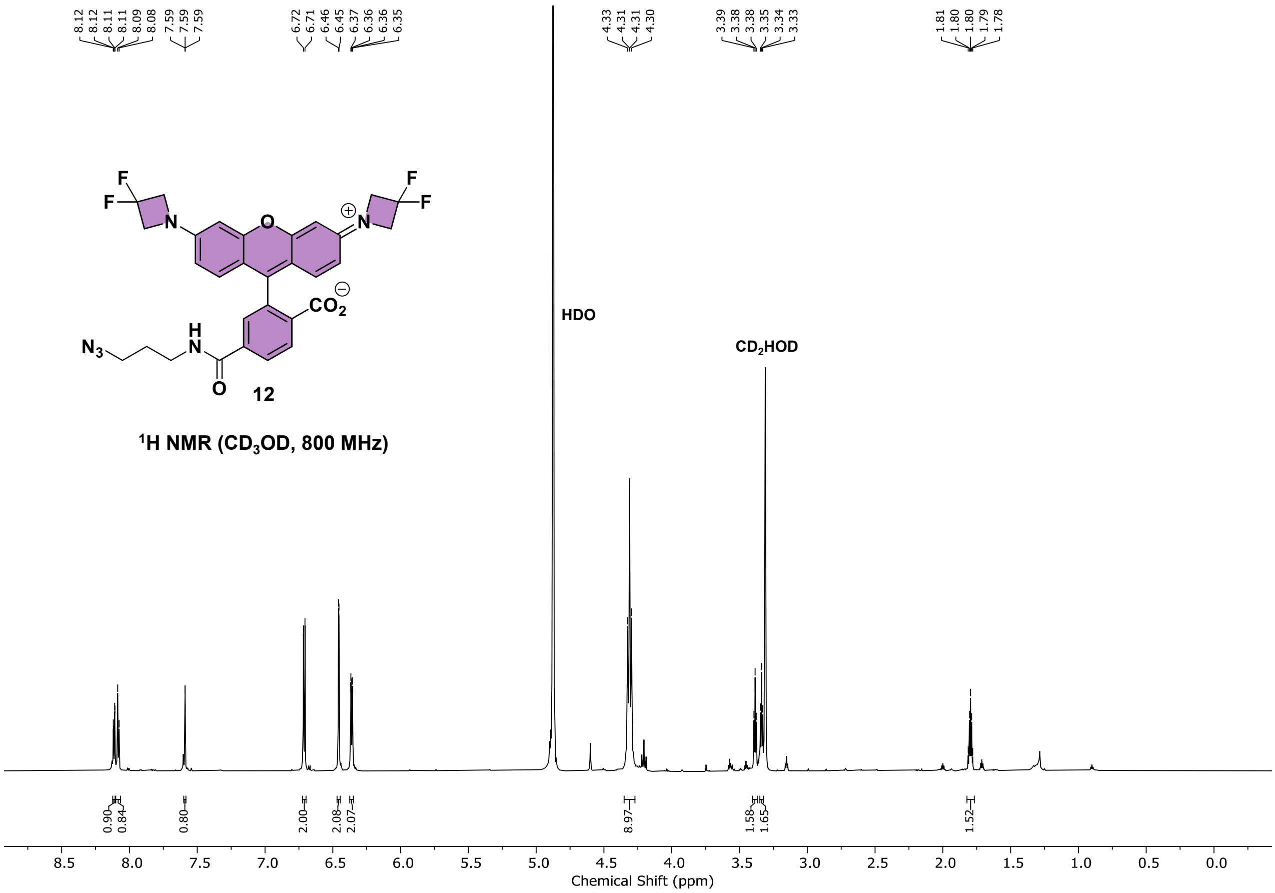
8.12
8.12
8.11
8.11
8.09
8.08
7.59
7.59
7.59
6.72
6.71
6.46
6.45
6.37
6.36
6.36
6.35
4.33
4.31
4.31
4.30
3.39
3.38
3.38
3.35
3.34
3.33
1.81
1.80
1.80
1.79
1.78
HDO
CD2HOD
12
1H NMR (CD3OD, 800 MHz)
0.90
0.84
0.80
2.00
2.08
2.07
8.97
1.58
1.65
1.52
Chemical Shift (ppm)

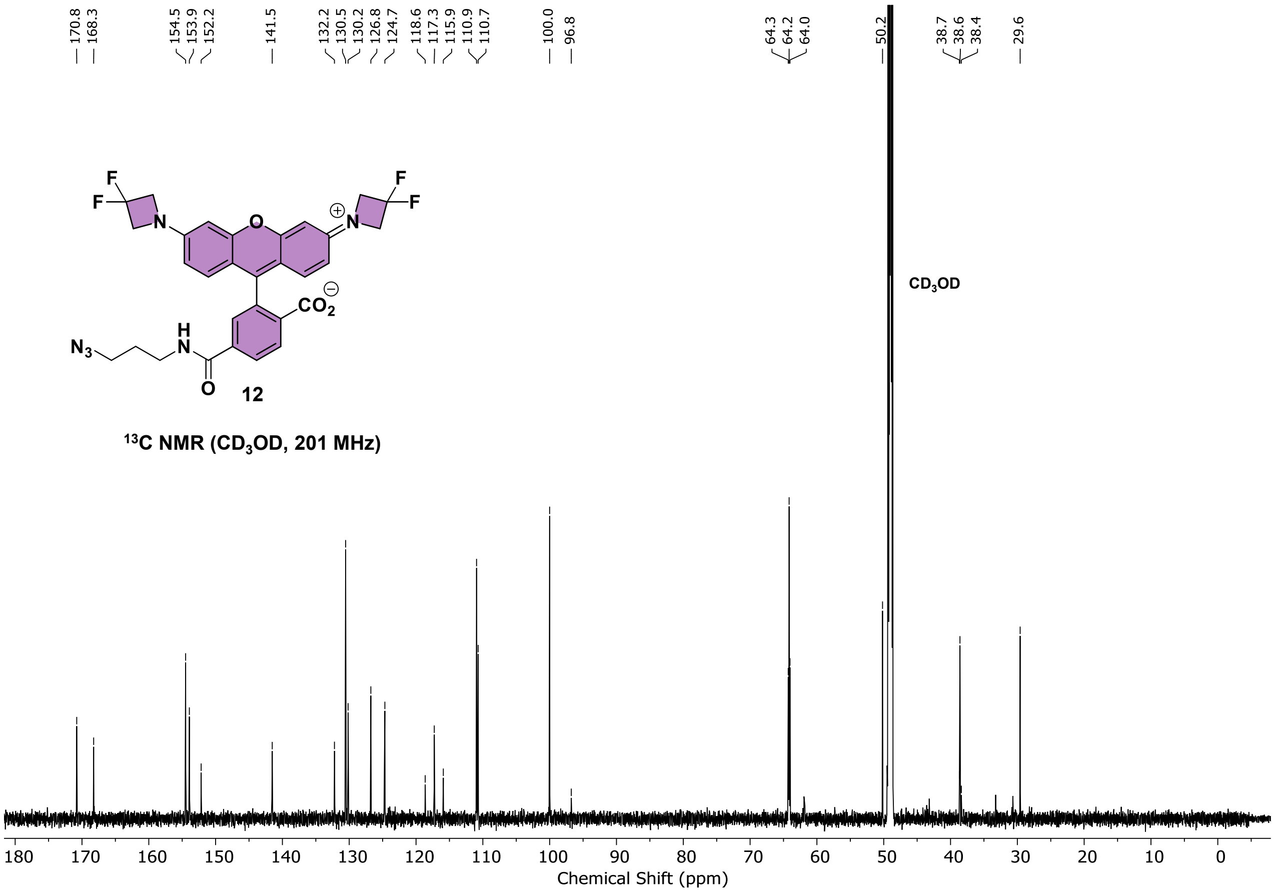

170.8
168.3
154.5
153.9
152.2
141.5
132.2
130.5
130.2
126.8
124.7
118.6
117.3
115.9
110.9
110.7
100.0
96.8
64.3
64.2
64.0
50.2
38.7
38.6
38.4
29.6
CD3OD
12
13C NMR (CD3OD, 201 MHz)
Chemical Shift (ppm)

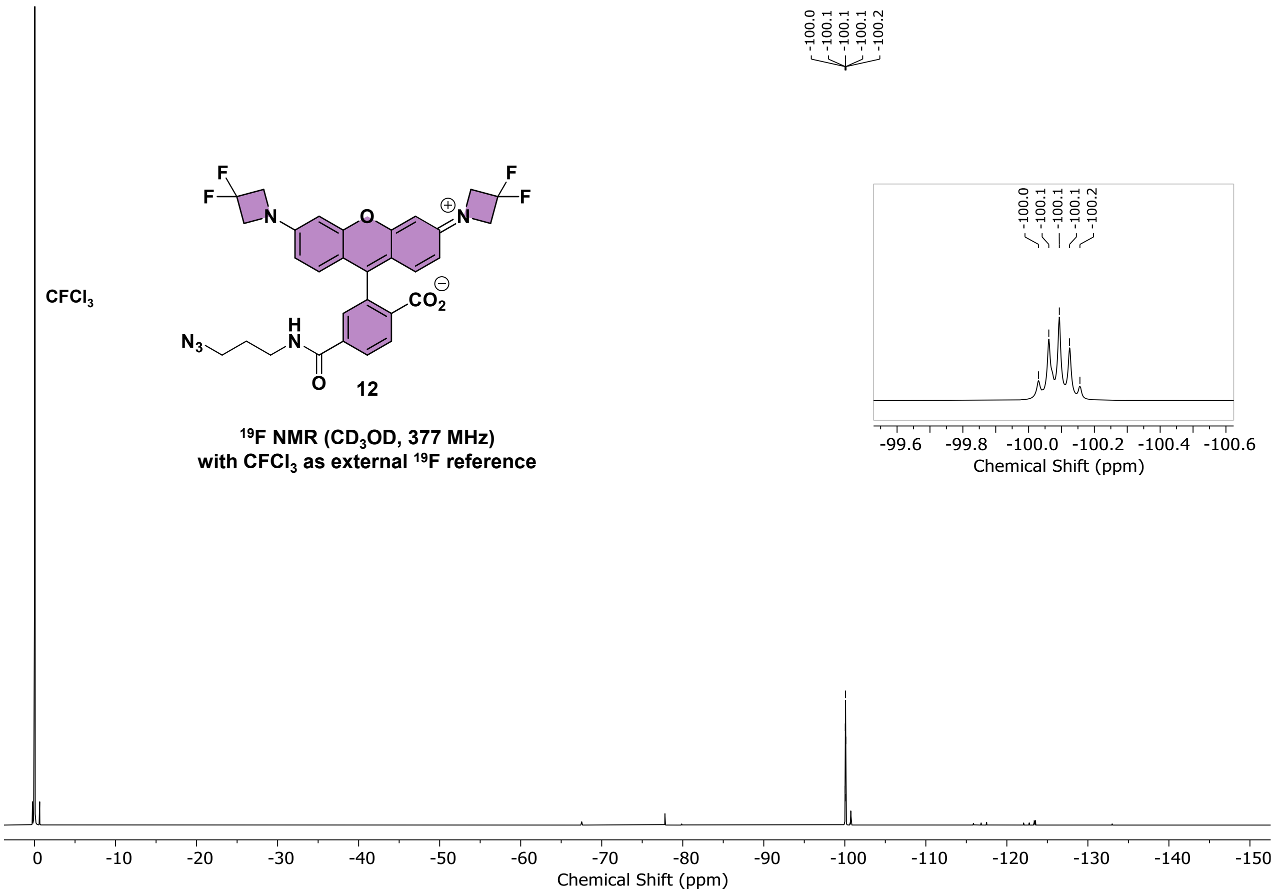

-100.0
-100.1
-100.1
-100.1
-100.2
CFCl3
12
19F NMR (CD3OD, 377 MHz)
with CFCl3 as external 19F reference
Chemical Shift (ppm)

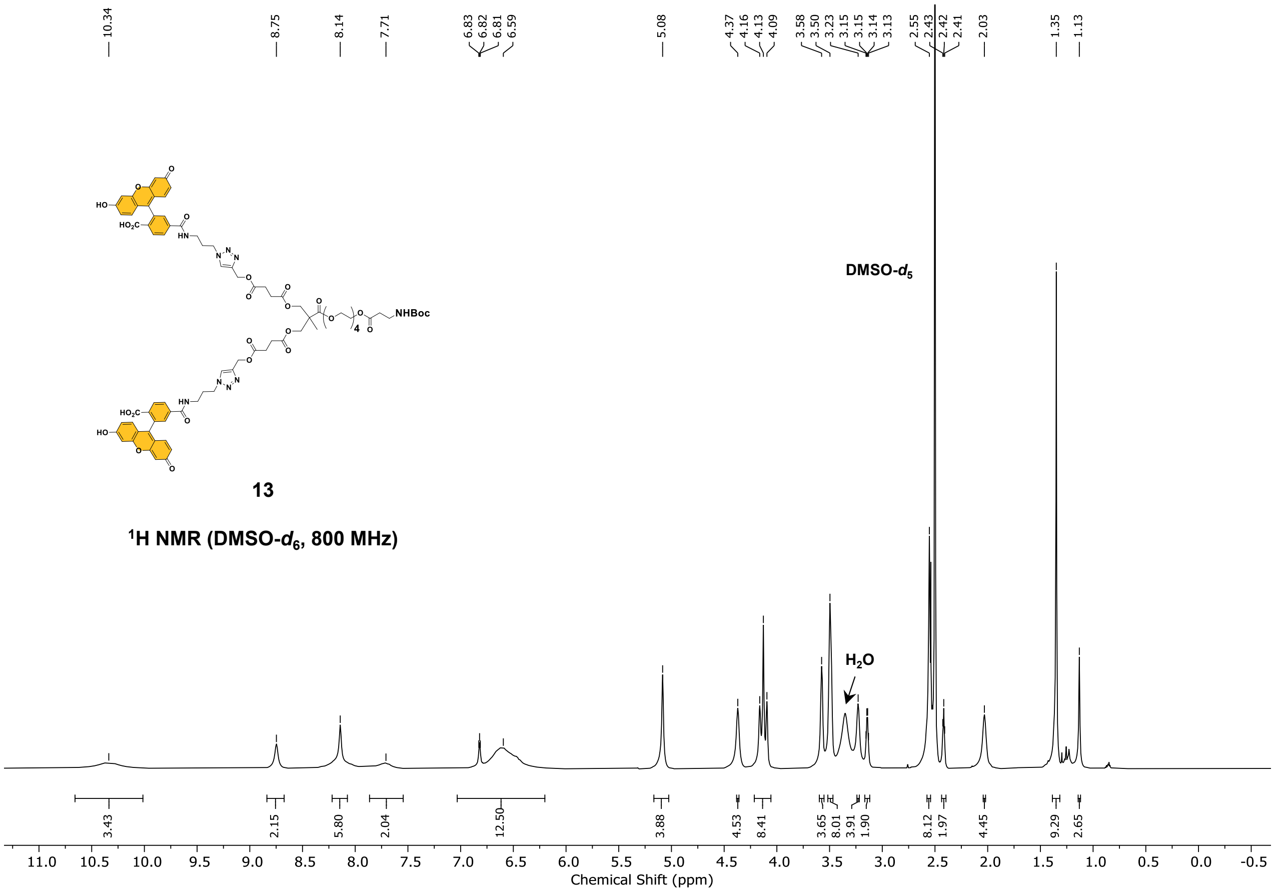
10.34
8.75
8.14
7.71
6.83
6.82
6.81
6.59
5.08
4.37
4.16
4.13
4.09
3.58
3.50
3.23
3.15
3.15
3.14
3.13
2.55
2.43
2.42
2.41
2.03
1.35
1.13
HO
HO2C
HN
NHBoc
13
1H NMR (DMSO-d6, 800 MHz)
DMSO-d5
H2O
3.43
2.15
5.80
2.04
12.50
3.88
4.53
8.41
3.65
8.01
3.91
1.90
8.12
1.97
4.45
9.29
2.65
Chemical Shift (ppm)

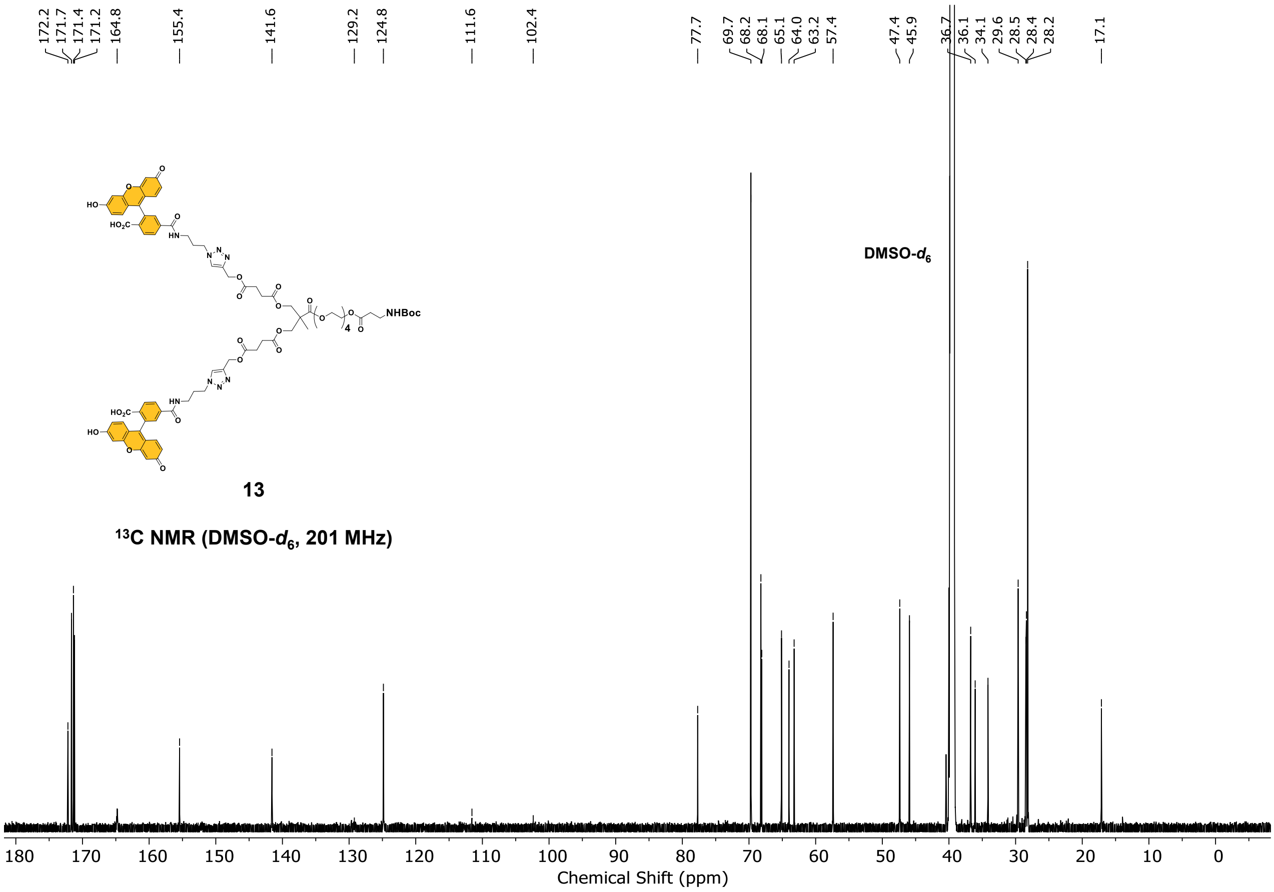
172.2
171.7
171.4
171.2
164.8
155.4
141.6
129.2
124.8
111.6
102.4
77.7
69.7
68.2
68.1
65.1
64.0
63.2
57.4
47.4
45.9
36.7
36.1
34.1
29.6
28.5
28.4
28.2
17.1
HO
HO2C
HN
NHBoc
13
13C NMR (DMSO-d6, 201 MHz)
DMSO-d6
Chemical Shift (ppm)

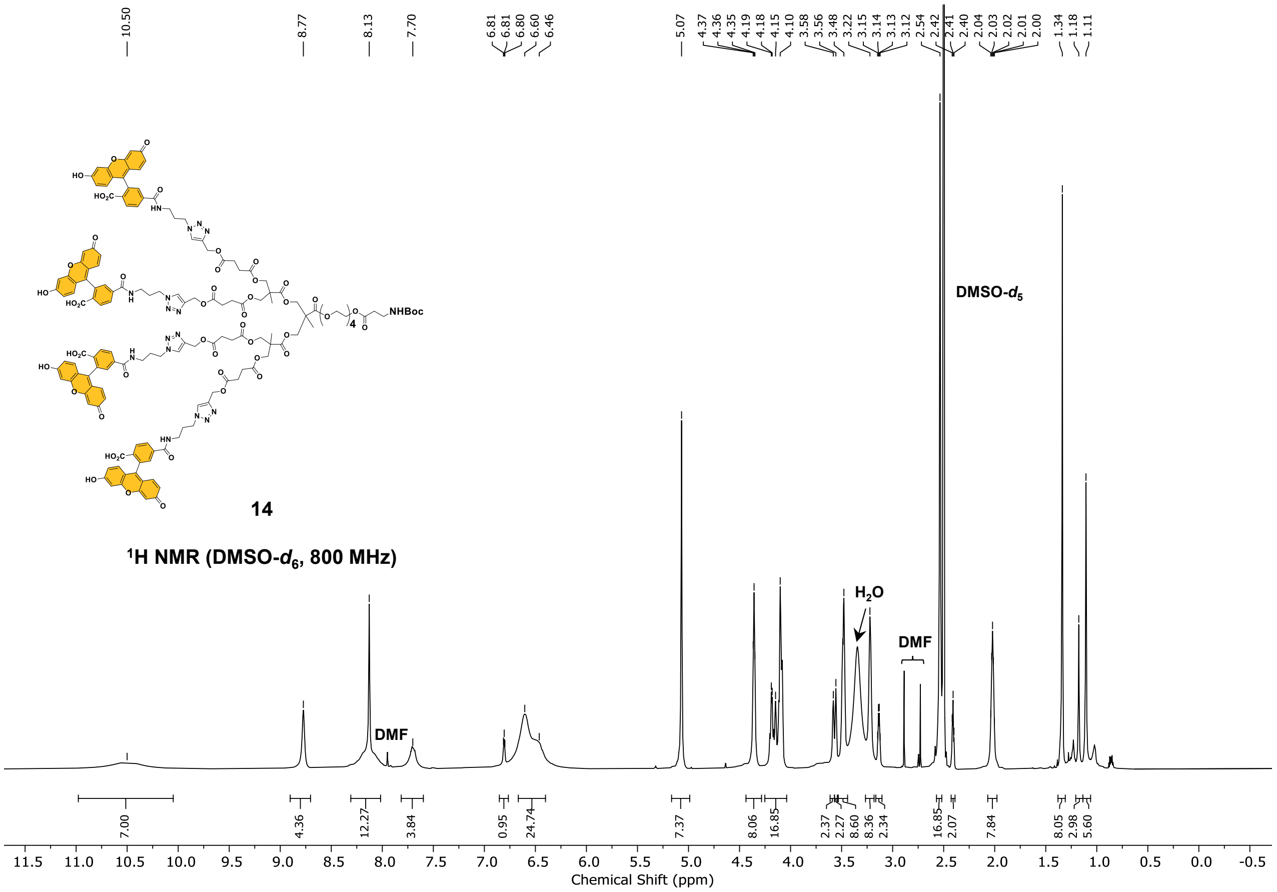
10.50, 8.77, 8.13, 7.70, 6.81, 6.81, 6.80, 6.60, 6.46, 5.07, 4.37, 4.36, 4.35, 4.19, 4.18, 4.15, 4.10, 3.58, 3.56, 3.48, 3.22, 3.15, 3.14, 3.13, 3.12, 2.54, 2.42, 2.41, 2.40, 2.04, 2.03, 2.02, 2.01, 2.00, 1.34, 1.18, 1.11
HO
HO₂C
NHBoc
14
¹H NMR (DMSO-d₆, 800 MHz)
DMSO-d₅
H₂O
DMF
DMF
7.00, 4.36, 12.27, 3.84, 0.95, 24.74, 7.37, 8.06, 16.85, 2.37, 2.27, 8.60, 8.36, 2.34, 16.85, 2.07, 7.84, 8.05, 2.98, 5.60
Chemical Shift (ppm)

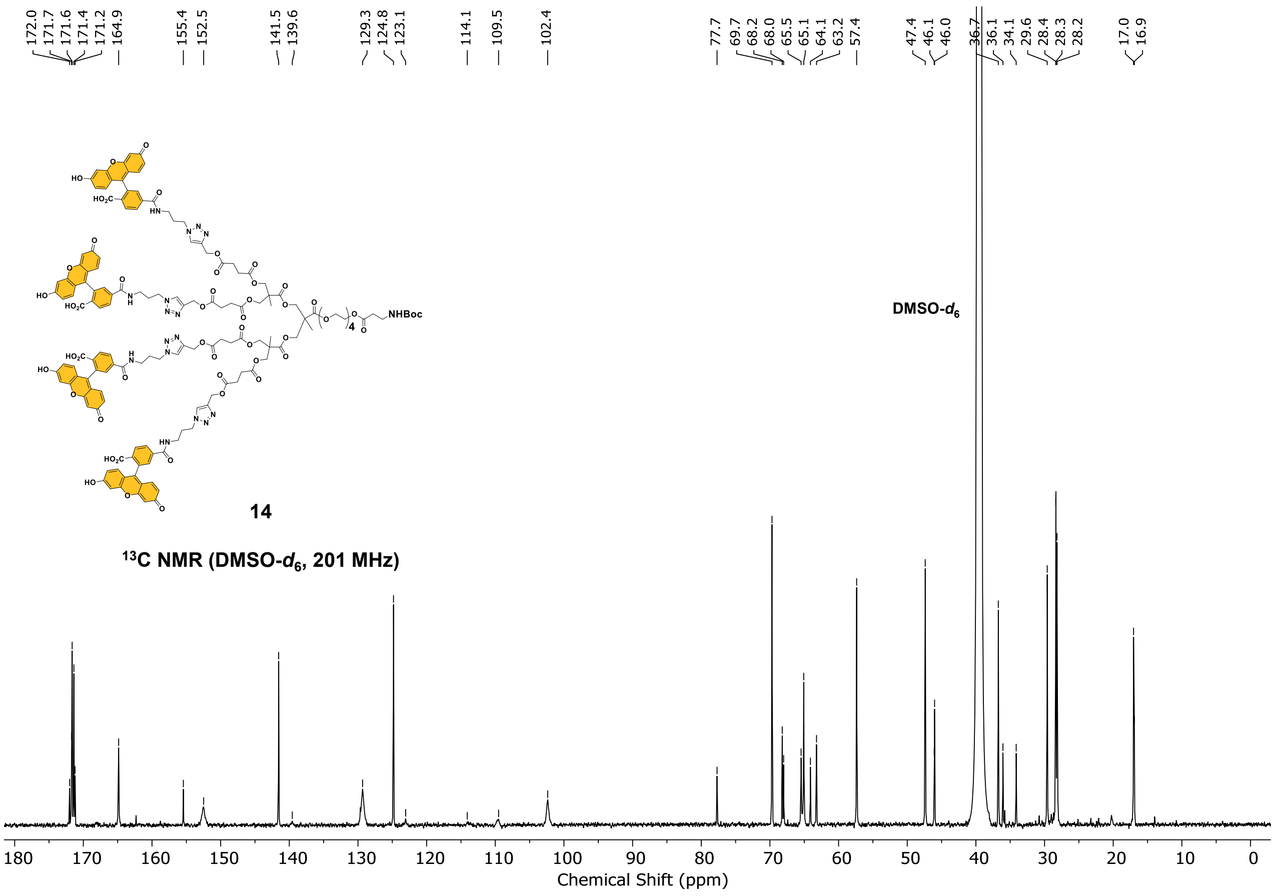
172.0, 171.7, 171.6, 171.4, 171.2, 164.9, 155.4, 152.5, 141.5, 139.6, 129.3, 124.8, 123.1, 114.1, 109.5, 102.4, 77.7, 69.7, 68.2, 68.0, 65.5, 65.1, 64.1, 63.2, 57.4, 47.4, 46.1, 46.0, 36.7, 36.1, 34.1, 29.6, 28.4, 28.3, 28.2, 17.0, 16.9
HO
HO₂C
NHBoc
14
¹³C NMR (DMSO-d₆, 201 MHz)
DMSO-d₆
Chemical Shift (ppm)

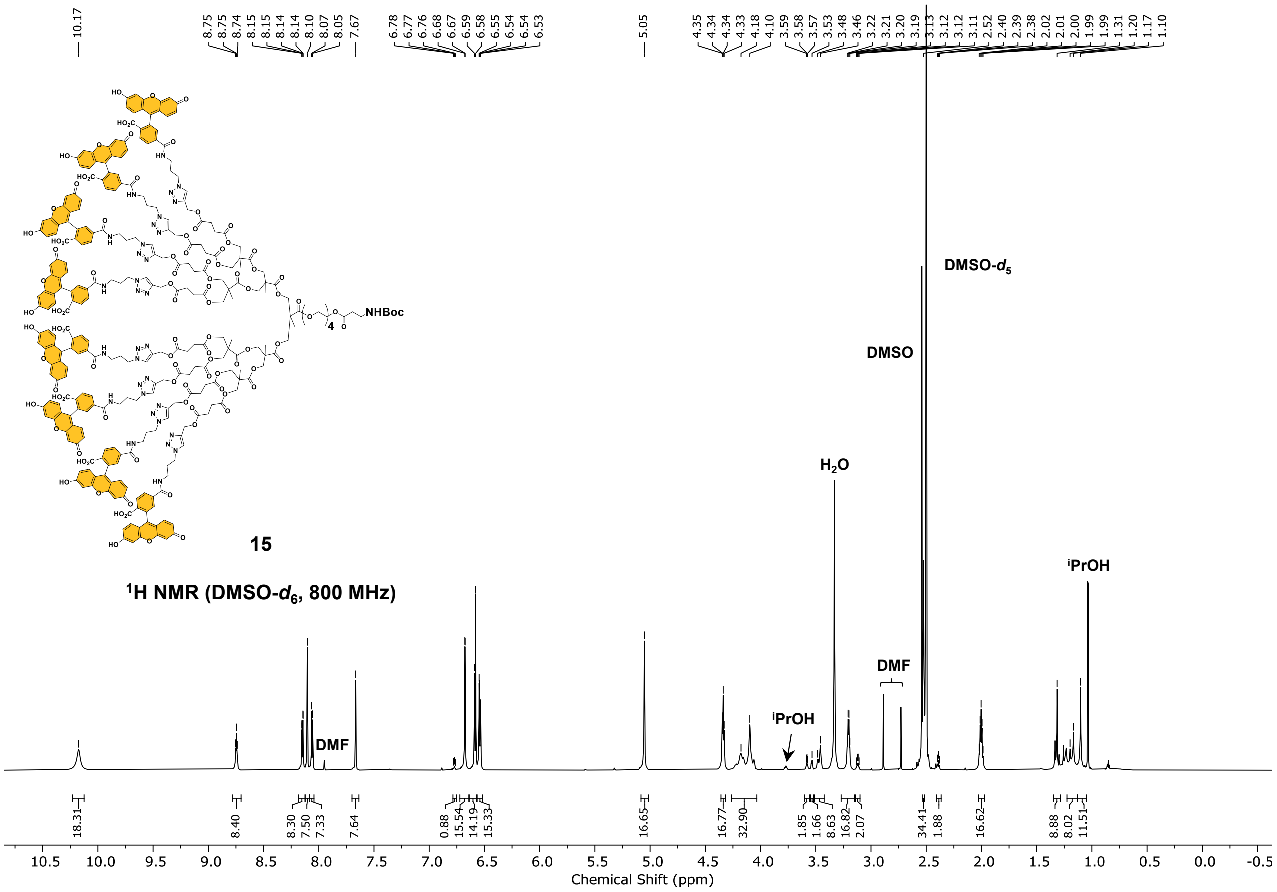
15
^{1}H NMR (DMSO-d_6, 800 MHz)
DMSO-d_5
DMSO
H_2O
DMF
^{i}PrOH
Chemical Shift (ppm)

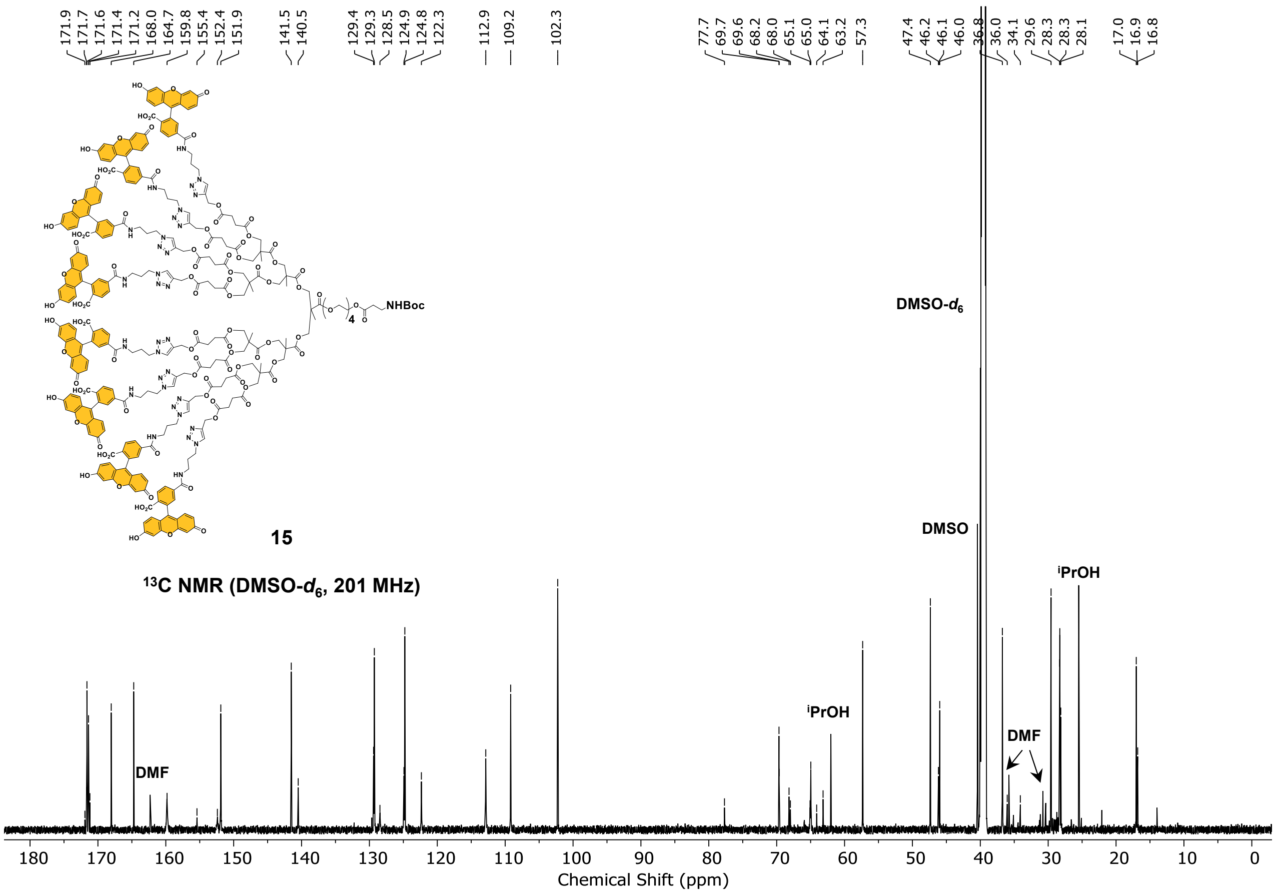
15
^{13}C NMR (DMSO-d_6, 201 MHz)
DMSO-d_6
DMSO
^{i}PrOH
DMF
Chemical Shift (ppm)

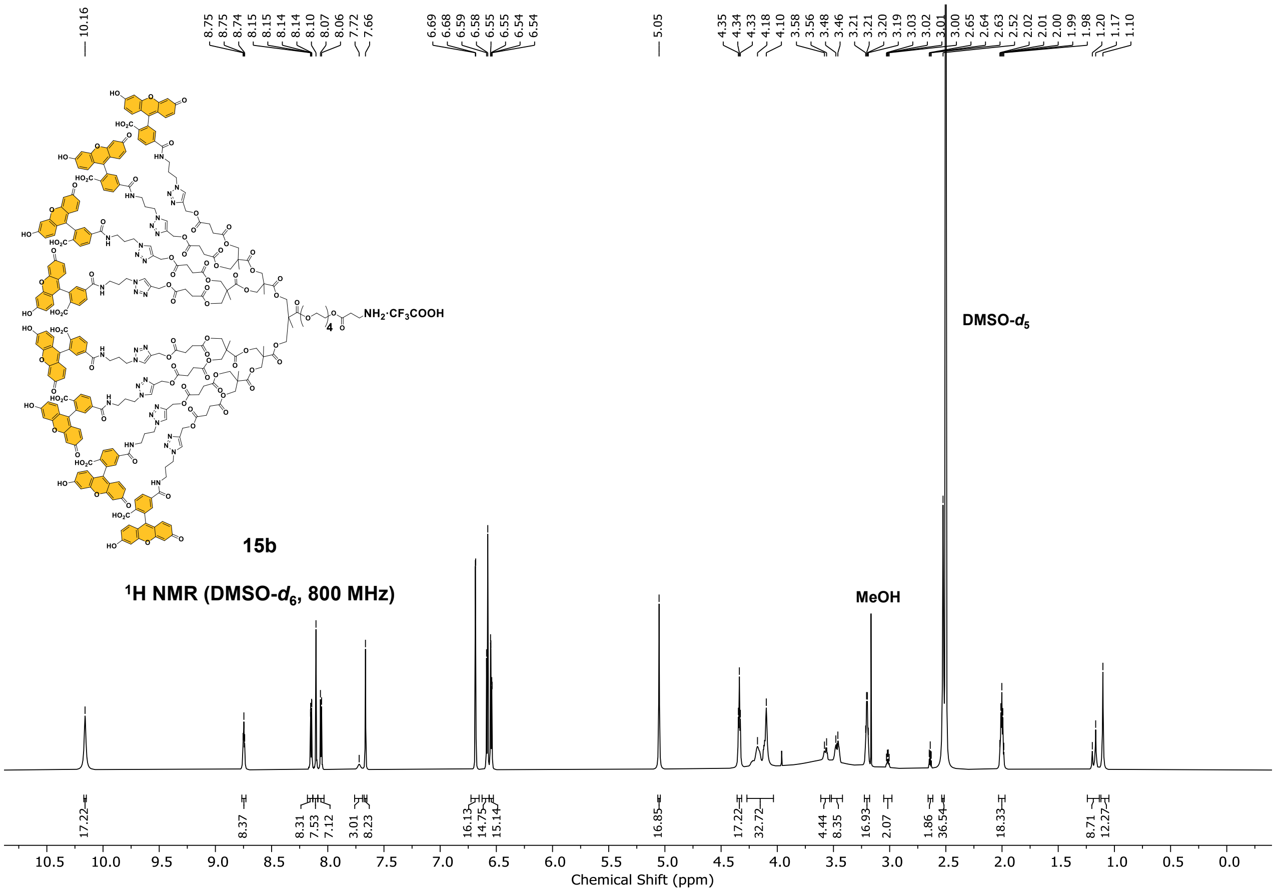
10.16
8.75 8.75 8.74 8.15 8.15 8.14 8.14 8.10 8.07 8.06 7.72 7.66
6.69 6.68 6.59 6.58 6.55 6.55 6.54 6.54
5.05
4.35 4.34 4.33 4.18 4.10 3.58 3.56 3.48 3.46 3.21 3.20 3.19 3.03 3.02 3.01 3.00 2.65 2.64 2.63 2.52 2.02 2.01 2.00 1.99 1.98 1.20 1.17 1.10
NH2·CF3COOH
15b
1H NMR (DMSO-d6, 800 MHz)
DMSO-d5
MeOH
17.22 8.37 8.31 7.53 7.12 3.01 8.23 16.13 14.75 15.14 16.85 17.22 32.72 4.44 8.35 16.93 2.07 1.86 36.54 18.33 8.71 12.27
Chemical Shift (ppm)

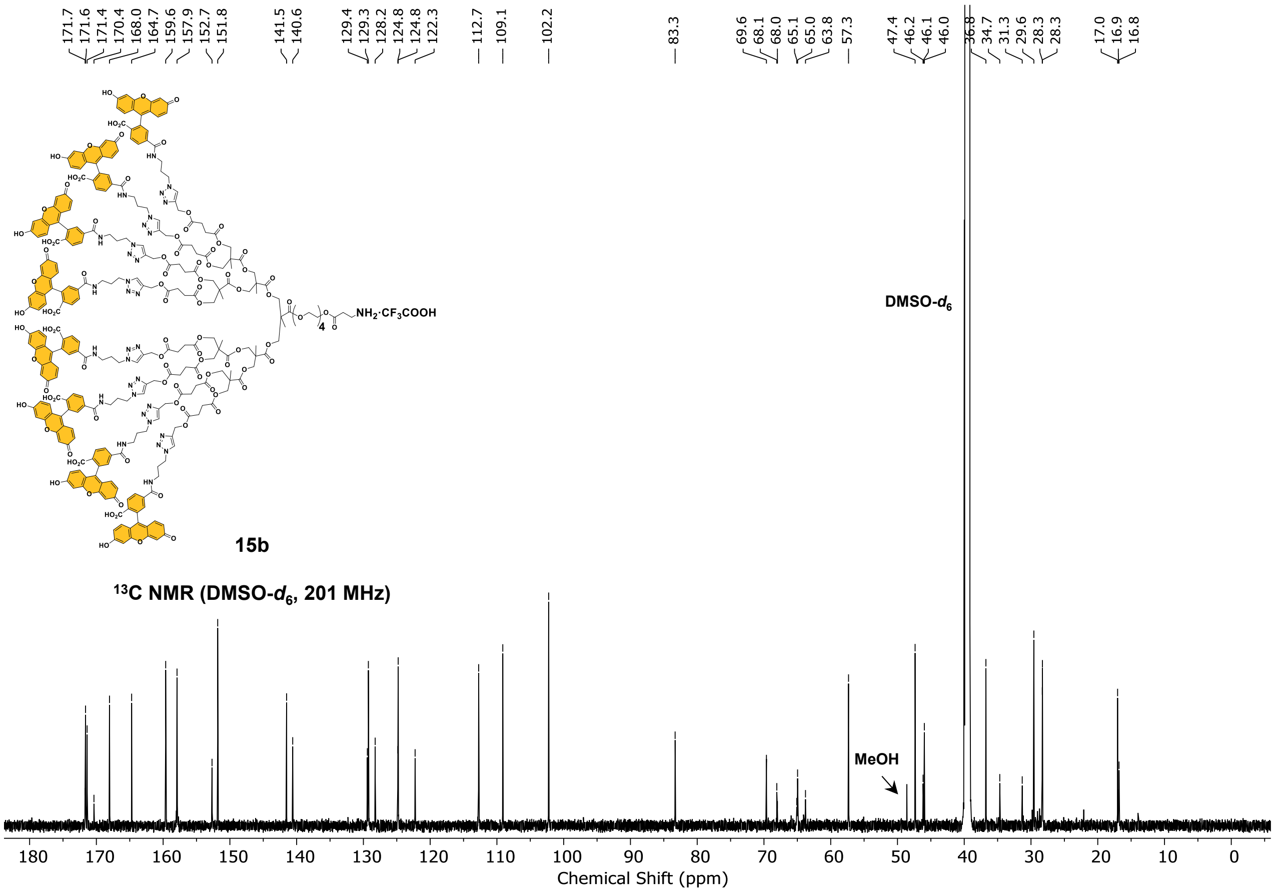
171.7 171.6 171.4 170.4 168.0 164.7 159.6 157.9 152.7 151.8
141.5 140.6
129.4 129.3 128.2 124.8 124.8 122.3
112.7 109.1 102.2
83.3
69.6 68.1 68.0 65.1 65.0 63.8 57.3
47.4 46.2 46.1 46.0 36.8 34.7 31.3 29.6 28.3 28.3
17.0 16.9 16.8
NH2·CF3COOH
15b
13C NMR (DMSO-d6, 201 MHz)
DMSO-d6
MeOH
Chemical Shift (ppm)

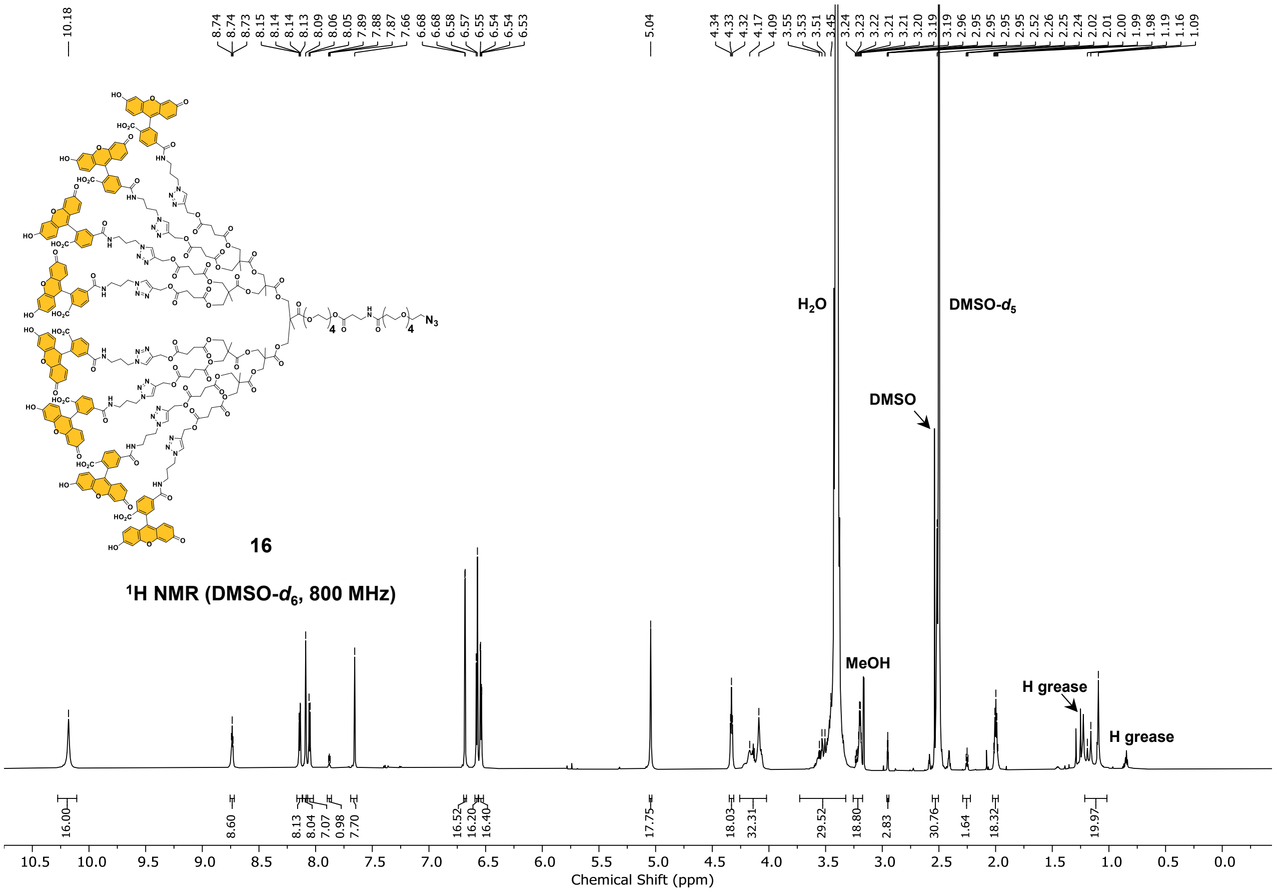

16

^{1}H NMR (DMSO-d_6, 800 MHz)

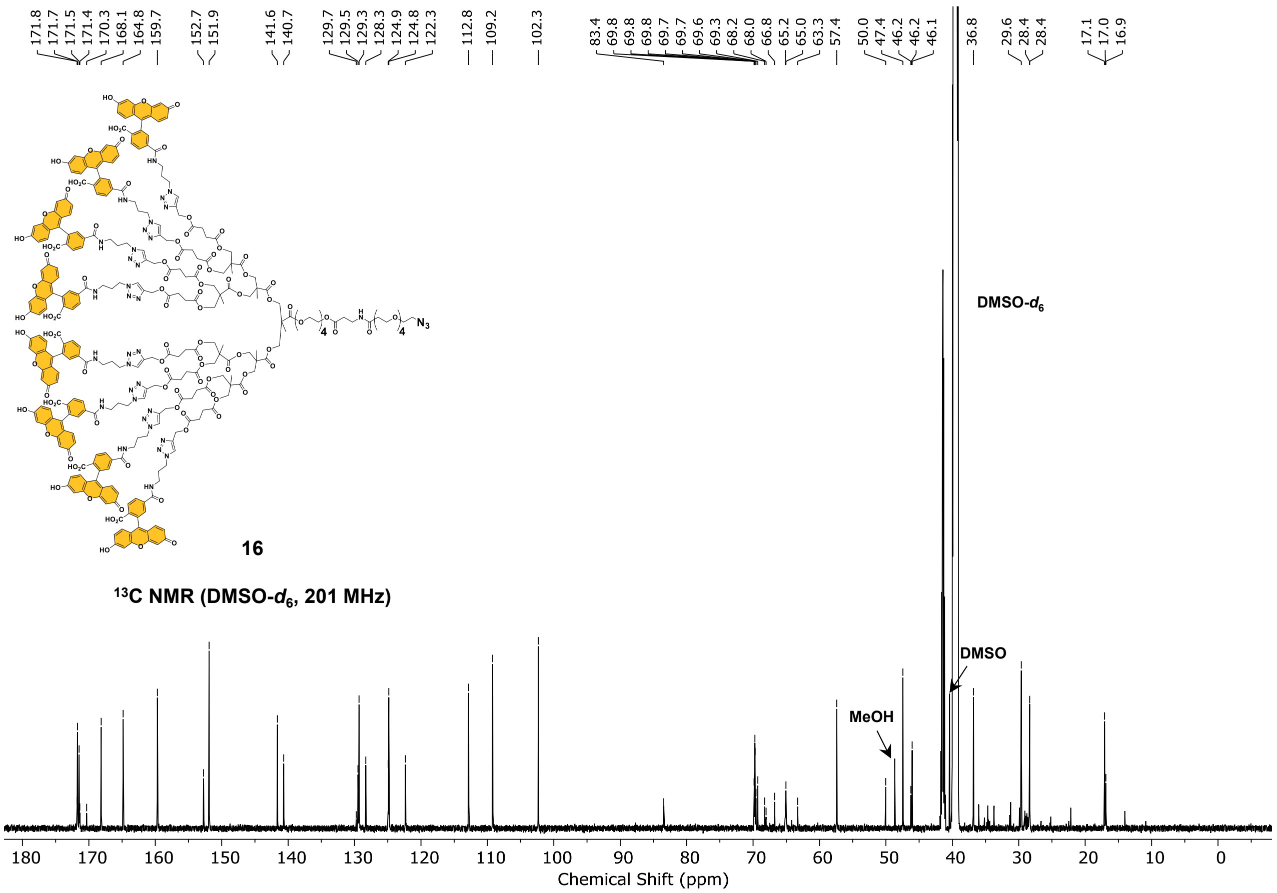

16

^{13}C NMR (DMSO-d_6, 201 MHz)

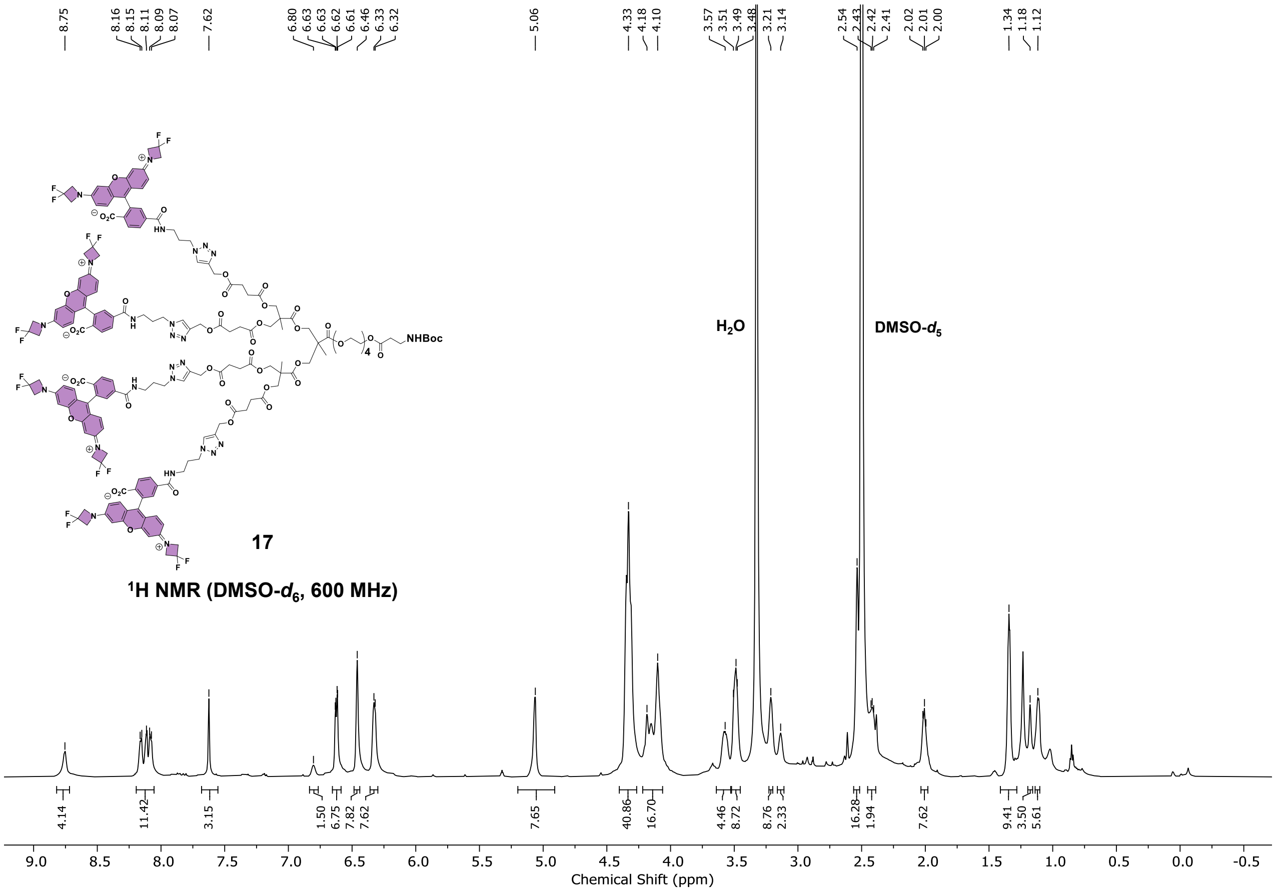

17
^{1}H NMR (DMSO-d_6, 600 MHz)
H_2O
DMSO-d_5
Chemical Shift (ppm)

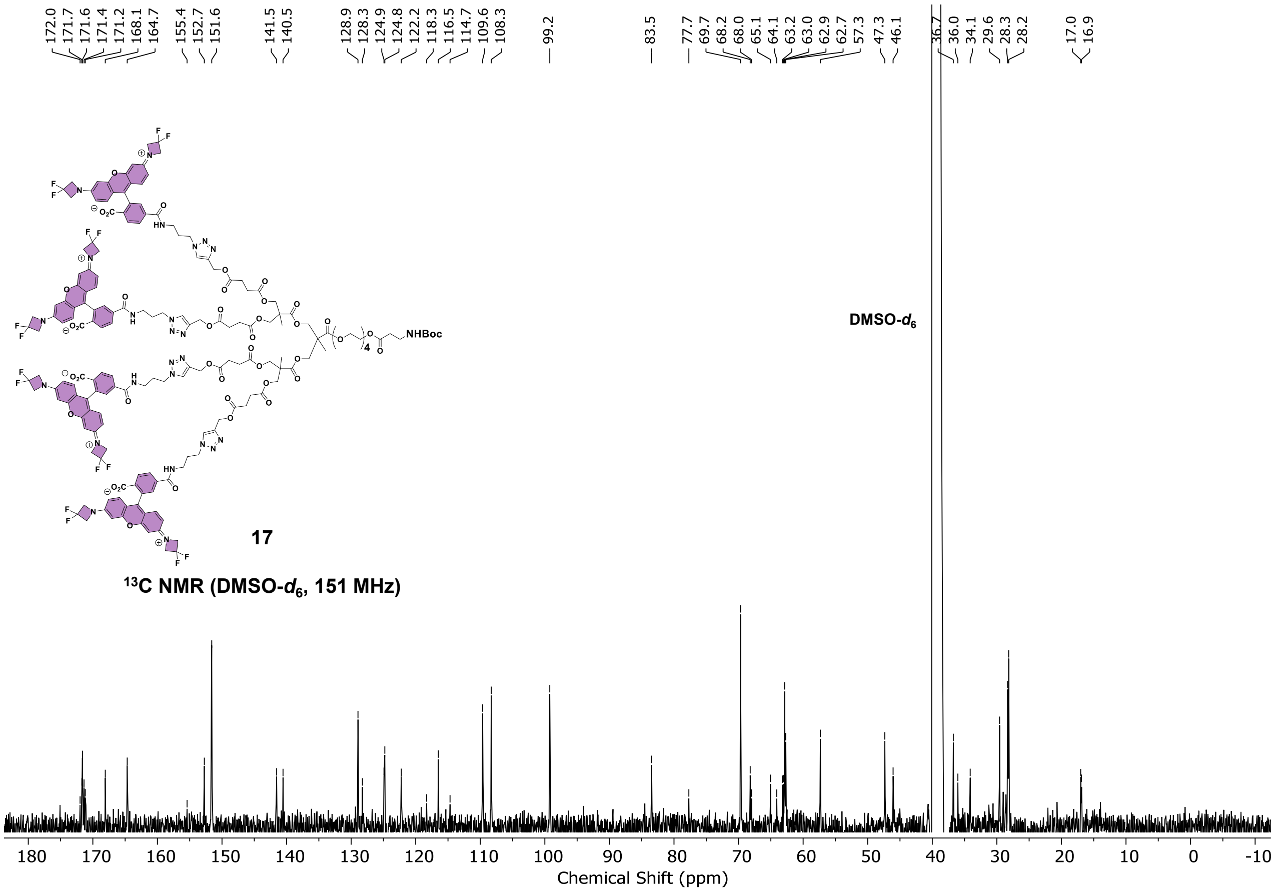

17
^{13}C NMR (DMSO-d_6, 151 MHz)
DMSO-d_6
Chemical Shift (ppm)

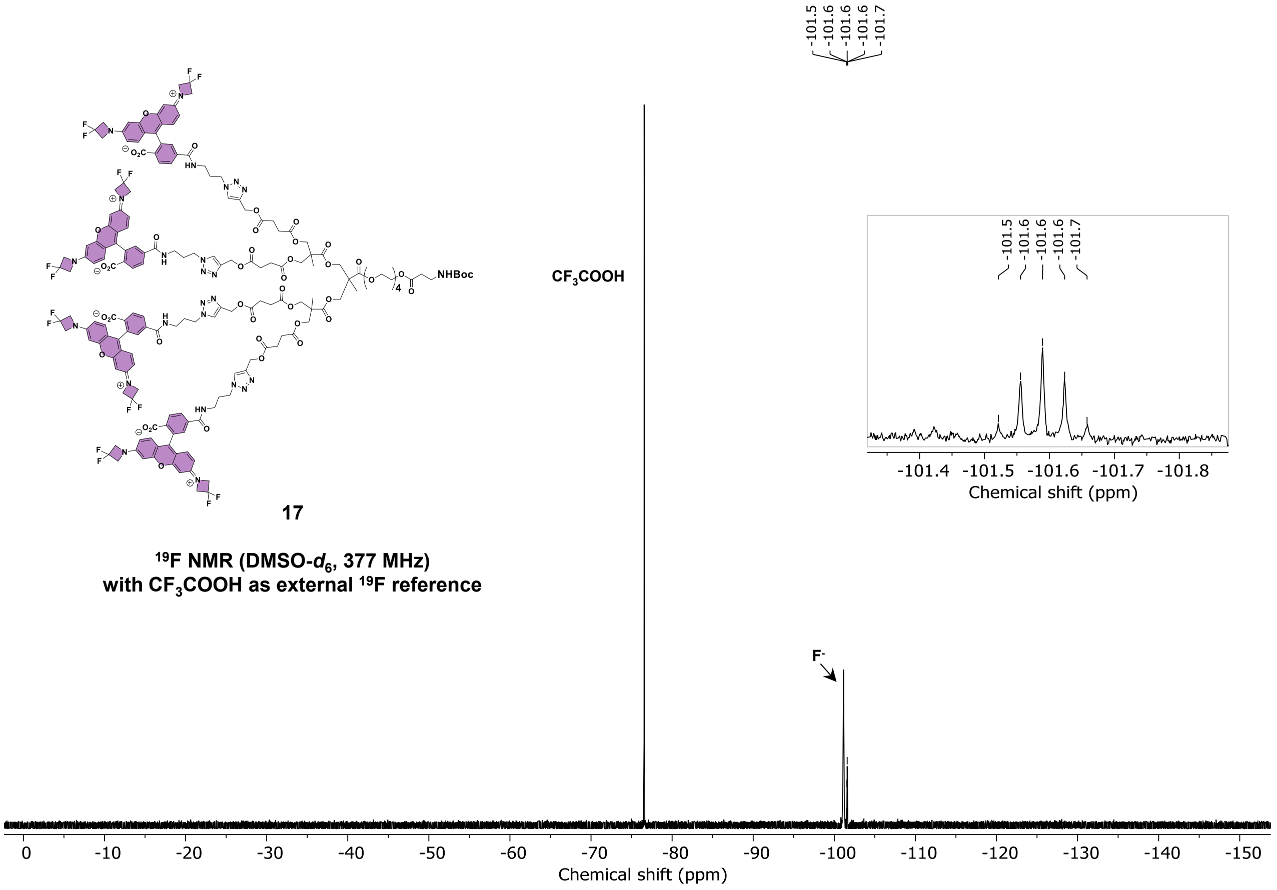

NOTE: The resonance at -101.1 ppm was tentatively assigned as soluble ionic fluoride (F^-).[4,5]

9. References

[1] F. Parenti, F. Tassinari, E. Libertini, M. Lanzi, A. Mucci, *ACS Omega* **2017**, *2*, 5775–5784.

[2] G. Y. Wiederschain, *The Molecular Probes Handbook. A Guide to Fluorescent Probes and Labeling Technologies*, Springer, **2011**.

[3] M. Beija, C. A. M. Afonso, J. M. G. Martinho, *Chem. Soc. Rev.* **2009**, *38*, 2410–2433.

[4] A. Sadoc, M. Body, C. Legein, M. Biswal, F. Fayon, X. Rocquefelte, F. Boucher, *Phys. Chem. Chem. Phys.* **2011**, *13*, 18539–18550.

[5] A. Rehmer, K. Scheurell, E. Kemnitz, *J. Mater. Chem. C* **2015**, *3*, 1716–1723.

10. Author Contributions

L. M. C. Conceptualization **(Equal)**; Investigation and Methodology (**Lead**); Formal Analysis **(Lead)**; Data Curation and Validation **(Lead)**; Preparation of the Manuscript Draft **(Lead)**.

F. S. Investigation and Methodology **(Supporting)**; Formal Analysis **(Supporting)**; Data Curation and Validation **(Supporting)**; Preparation of the Manuscript Draft **(Supporting)**; Supervision **(Supporting)**.

A. C. Data Curation and Validation **(Supporting)**; Preparation of the Manuscript Draft **(Supporting)**; Supervision **(Supporting)**.

S. S. Conceptualization **(Equal)**; Supervision **(Supporting)**.

T. C. Conceptualization **(Supporting)**; Funding Acquisition **(Supporting)**; Project Administration **(Supporting)**.

T. F. Conceptualization **(Supporting)**; Funding Acquisition **(Lead)**; Project Administration **(Lead)**; Supervision **(Lead)**.
